# Determinants of generalized trust: Economic, Institutional, and personal factors

**DOI:** 10.12688/openreseurope.22835.1

**Published:** 2026-04-17

**Authors:** Jorge Soria Ruiz-Ogarrio, Jorge Moya Velasco, Carlos Estevez Mendoza, Josep Navarro-Ortiz

**Affiliations:** 1 Facultad de Económicas, Universidad de Navarra, Pamplona, Navarre, Spain; 2 Facultad de Derecho, Empresa y Ciencias Políticas, Universidad Cardenal Herrera-CEU, Universidades CEU, Alfara del Patriarca, Valencian Community, Spain; 3 Facultad de Ciencias Económicas y Empresariales, Universidad Complutense de Madrid, Madrid, Spain and Facultad de Empresa, Economía y Derecho, CUNEF, Universidad, Madrid, Spain; 4 Analsisi Economic, Universitat de Valencia - Campus dels Tarongers, Valencia, Valencian Community, Spain

**Keywords:** trust, generalized trust, particularized trust, social capital, institutions, economic development, education, probit models.

## Abstract

**Background:**

Generalized trust is a central component of social cohesion and development, yet its determinants remain diverse and contested. While much of the literature examines trust as a driver of growth, this study explores the structural, institutional, and behavioral conditions that enable trust to emerge.

**Methods:**

This study examines three domains of generalized trust—trust in most people (TMP), trust in people met for the first time (TFT), and trust in neighbors (TN)—using an integrative framework that combines macro- and micro-level variables from the World Values Survey (2018–2022). The analysis covers 97,200 respondents across a diverse set of countries. Applying probit and ordered probit models, we incorporate structural factors such as national wealth, inequality, and institutional stability, alongside individual characteristics related to income, education, religiosity, and tolerance.

**Results:**

Findings show that higher GDP per capita, national educational development, and personal income consistently increase trust across all domains. Conversely, income inequality and urbanization tend to erode trust. Tolerance emerges as a consistently positive predictor. A nuanced “religiosity paradox” is observed: higher religious importance shifts trust toward closer social circles (TN) while reducing openness to strangers (TFT). Furthermore, while national educational development fosters trust, individual schooling often fosters more cautious attitudes.

**Conclusions:**

Trust is a multicausal construct shaped by the intersection of economic security, institutional legitimacy, and inclusive social norms. Strengthening generalized trust requires public policies that foster inclusive growth, equitable education, and civic values of openness and tolerance. These results offer one of the most comprehensive cross-national examinations of generalized trust to date, highlighting the importance of extending trust beyond familiar networks.

## Plain Language summary

This research investigates the complex foundations of social trust by examining how 97,200 individuals across the globe perceive most people, strangers, and their own neighbors. The study reveals that trust is not a fixed cultural trait but is heavily influenced by a person’s economic and social surroundings. On a broad level, people living in wealthy countries and those with higher personal incomes are significantly more likely to trust others, as financial security provides a “safety net” that allows for greater openness. Conversely, high levels of income inequality and the anonymity of dense urban living act as “trust-killers,” consistently weakening social ties and making people more cautious toward those around them.

Beyond money and geography, personal values and education play surprisingly nuanced roles in shaping our social outlook. While strong national education systems foster more trusting societies overall, individuals with many years of formal schooling often develop a more critical and skeptical mindset, leading to increased caution in their personal interactions. A similar “paradox” exists with religion: deeply religious individuals often report high trust in their neighbors but are more likely to be wary of strangers. Ultimately, the study highlights that tolerance of different beliefs is one of the most powerful and consistent predictors of high trust across all categories. These findings suggest that to build more cooperative communities, policies must focus on promoting fairness, inclusive growth, and the civic values of openness.

## 1. Introduction

The aim of this research is to identify the factors that contribute to the formation of trust, a central component of social capital and a key resource for economic and social development, long recognized in the literature for its association with long-term growth ( Bjørnskov 2012; Knack, 2002; Zak and Knack, 2001). It supports human capital formation by encouraging cooperative behaviour ( Coleman, 1988; Papagapitos and Riley, 2009), reduces transaction costs ( Arrow, 1972; Zak and Knack, 2001), and enhances institutional quality by promoting accountability and institutional legitimacy ( Arrow, 1972; Fukuyama 1995; Knack and Zak, 2002; Putnam, 1993; Rothstein and Stolle, 2003; Zak and Knack, 2001).

These mechanisms are embedded in theoretical frameworks such as the Solow model and its extensions incorporating human capital ( Mankiw, Romer, and Weil, 1992; Solow, 1956), where trust functions as an intangible element of social capital that supports steady-state accumulation. This framework is adopted solely as a conceptual reference to situate trust within broader development dynamics, not as the empirical focus of the study. While the literature has extensively examined trust as a mechanism that enhances economic performance, this study adopts the reverse perspective, exploring the structural, institutional, and behavioural conditions that foster interpersonal trust. Accordingly, this research focuses on the determinants of trust rather than on its consequences for economic growth.

Although most of the literature has examined trust as a driver of growth, a complementary line of research has sought to understand the reverse relationship: the economic, institutional, and social conditions that enable trust to emerge. This study follows that tradition, investigating the structural and behavioural foundations that make trust possible ( Berggren and Jordahl, 2006; Brandt, Wetherell, and Henry, 2015; Delhey and Newton, 2005; Glatz and Schwerdtfeger, 2022; Zmerli and Newton, 2008). In doing so, it responds to ongoing calls in the literature to disentangle the contextual and micro-level mechanisms shaping trust across diverse societies ( Beugelsdijk, de Groot, and van Schaik, 2004; Mikucka, Sarracino, and Dubrow, 2017). Recent contributions highlight that institutional quality and equality serve as enabling environments for trust formation ( Delhey and Newton, 2005; Knack, 2021; Rothstein and Uslaner, 2005), while historical legacies, religion, and perceptions of democracy leave enduring imprints on societal trust ( Tabellini, 2008). At the micro level, education, income, and lived experiences influence expectations about others’ trustworthiness ( Alesina and Ferrara, 2002; Brandt, Wetherell, and Henry, 2015; Green and Nelson, 2019).

Empirical studies have further demonstrated how inequality ( Bjørnskov, 2007; Mikucka, Sarracino, and Dubrow, 2017), social context ( Delhey and Dragolov, 2016; Newton, 2004; Putnam, 2001; Putnam, Leonardi, and Nanetti, 1994), and demographic characteristics, such as age or life expectancy ( Berggren and Jordahl, 2006), affect trust formation. Religious affiliation and tolerance toward other beliefs also play a role, as hierarchical religious traditions may correlate negatively with generalized trust ( La Porta et al., 1996; Uslaner, 2002). These findings suggest that trust is shaped simultaneously by macro-structural environments and micro-level attitudes and experiences.

Methodological challenges persist in measuring and interpreting these relationships. Sample composition and unobserved heterogeneity can influence results ( Beugelsdijk, de Groot, and van Schaik, 2004; Mikucka, Sarracino, and Dubrow, 2017), and endogeneity between trust and development remains a central concern. To address these limitations, this study com- bines structural national-level indicators with individual-level variables drawn from the latest wave of the World Values Survey (WVS-2022) ( Ahlerup, Olsson, and Yanagizawa, 2009; Berggren and Jordahl, 2006; Bjørnskov, 2012, 2013; James, 2015; Mikucka, Sarracino, and Dubrow, 2017; Whiteley, 2000, Zak and Knack, 2001). This dataset offers a unique opportunity to analyse the joint effects of economic conditions, institutional stability, and personal attitudes across a large and diverse set of countries.

This study focuses on generalized trust, understood as trust in most people (TMP) and trust in people met for the first time (TFT). For completeness, we also include trust in neighbors (TN) as a bridging category. Although neighbors are not strangers, TN is commonly treated as an intermediate form of trust that lies between generalized and particularized trust. Including this category makes it possible to test whether the drivers of generalized trust also apply to socially proximate but non-kin relationships, without entering the domain of strictly particularized ties.

It is important to note that, despite its intermediate character, trust in neighbors (TN) should not be interpreted as a form of particularized trust. Particularized trust involves close, intimate, and kin-based relationships, such as family members or very close acquaintances, that rely on strong affective bonds and obligations. By contrast, TN reflects expectations toward proximate but socially heterogeneous others and therefore belongs to the broader domain of bridging trust. The empirical analysis treats TN accordingly, avoiding its conflation with family-based or kinship trust.

Probit and ordered probit models are estimated to identify the determinants of different trust levels and to compute average marginal effects for each domain. The results indicate that institutional quality, education, and life expectancy foster generalized trust, while income inequality and religiosity reduce openness toward generalized trust. Urbanization erodes all forms of trust, although tolerance mitigates these effects. By bridging macro- and micro-level analyses, this study contributes to a more integrated understanding of how trust is generated within complex social and institutional environments, offering insights into how inclusive policies and social cohesion can extend trust beyond familiar networks.

Building on this framework, the study contributes in three key ways. First, it adopts a *determinants* perspective, treating trust as the dependent variable and situating it within specific economic, institutional, and socio-demographic contexts ( Delhey and Newton, 2005; Knack, 2021; Rothstein and Uslaner, 2005). Second, it integrates macro and micro level predictors to explore how structural conditions and individual orientations jointly shape trust. Third, it employs ordered models and reports average marginal effects across *levels* of trust in multiple domains, enabling an assessment of whether aggregate associations reflect uniform shifts or redistributive patterns in trust. In doing so, the review of prior literature informs the empirical strategy, moving beyond broad cross-country correlations toward a level-specific, multi-domain analysis that clarifies when, and for whom, generalized trust is most likely to emerge.

The scope of this article is limited to generalized forms of trust; therefore, the determinants of particularized trust, such as those involving family and close interpersonal relationships, are better examined in a separate study.

The remainder of this paper is structured as follows: Section 2 presents the literature review; Section 3 describes the methodology, variables, and model specification; Section 4 reports the main findings; Section 5 explores the marginal effects of the variables on the principal types of trust; and Section 6 offers conclusions and policy implications.

## 2. Literature review

To situate our contribution within existing scholarship, this section reviews the main structural and micro-level determinants identified in the literature.

Trust is commonly defined as the willingness to accept vulnerability in situations of risk, grounded in the expectation that others will refrain from opportunistic behaviour ( Chiles and McMackin, 1996; MacCrimmon and Wehrung, 1986; Rousseau et al., 1998). It is typically conceptualized across three interrelated yet distinct dimensions: generalized trust, referring to trust among strangers ( Bjørnskov, 2007; Fukuyama, 1995; Misztal, 1996); particularized trust, embedded within cohesive and familiar networks ( Newton, 1997; Williams, 1988); and institutional trust, denoting confidence in political institutions, legal systems, and large organizations ( Luhmann, 2000). These distinctions relate to the idea of a radius of trust ( Delhey and Newton, 2005), which describes how far trust extends beyond intimate circles. Generalized trust represents the widest radius, directed toward strangers and abstract others, whereas particularized trust is embedded in close and homogeneous networks.

In this study, interpersonal trust is modelled as the outcome of interest, whereas institutional features are treated as antecedents and contextual enablers rather than dependent variables. Foundational studies have demonstrated a positive association between generalized trust and economic performance ( Aghion et al., 2010; Ahlerup, Olsson, and Yanagizawa, 2009; Berggren and Jordahl, 2006; Knack and Keefer, 1997; La Porta et al., 1996; Whiteley, 2000; Zak and Knack, 2001). Several mechanisms underpin this relationship. First, trust reduces transaction and verification costs, thereby facilitating exchanges and the enforcement of contracts ( Arrow, 1972; Bjørnskov, 2015; Dearmon and Grier, 2009; Fukuyama, 1995; Meyerson, Weick, and Kramer, 1996; North, 1990; Putnam, 1993; Whiteley, 2000). Second, it supports the formation of human capital by reinforcing cooperative norms and prosocial behaviour ( Bjørnskov, 2009; Coleman, 1988). Third, trust contributes to institutional quality by enhancing accountability and perceived legitimacy ( Knack, 2002; Putnam, 1993; Rothstein and Stolle, 2003). Empirical evidence further connects trust to productivity gains ( Ahmad and Hall, 2017; Bjørnskov, 2012), improved managerial and organizational practices ( Bloom and van Reenen, 2010), and positive firm-level outcomes ( Vanneste and Gulati, 2022).

Nonetheless, the trust–growth relationship presents both conceptual and empirical challenges. The direction of causality remains contested ( Delhey and Newton, 2005; Helliwell, 1996; Mikucka, Sarracino, and Dubrow, 2017). Excessive social organization may foster rent-seeking behaviour ( Olson, 1982; Putnam, 1995); and in high-trust societies, further in- creases in trust may yield diminishing or even adverse effects ( Roth, 2009; Zingales, 2011). Measurement issues further complicate the analysis: cross-country samples and unobserved heterogeneity can distort findings ( Beugelsdijk, de Groot, and van Schaik, 2004; Bjørnskov, 2007, 2018); proxies such as property rights enforcement may capture adjacent but distinct constructs ( Williamson and Kerekes, 2011); and alternative operationalizations, including fairness-based survey items, raise concerns about comparability ( Bauer and Freitag, 2018; Kanitsar, 2022; Uslaner, 1999). In response, recent scholarship has increasingly focused on intermediate mechanisms, particularly investment ( Bjørnskov, 2012, 2013; Dearmon and Grier, 2009), inequality ( Mikucka, Sarracino, and Dubrow, 2017), and institutional quality ( Ahlerup, Olsson, and Yanagizawa, 2009; Bjørnskov, 2013), and how these jointly shape the macroeconomic returns to trust.

A general review of the existing academic literature focused on the factors inducing trust reveals essentially two major complementary lines of research. The first employs structural and institutional factors to explain the generation of trust. Within this approach, and going beyond studies that use economic development as the basis of their analysis ( Mikucka, Sarracino, and Dubrow, 2017), there stand out studies that integrate the role of inequality and wealth with the quality of institutions ( Delhey and Newton, 2005; Rothstein and Stolle, 2003; Zmerli and Newton, 2008), analysing the connections between economic well-being, governance, and institutional security that foster the development of social capital and trust ( Berggren and Jordahl, 2006; Glatz and Schwerdtfeger, 2022; Muringani, Fitjar, and Rodríguez-Pose, 2024).

The second strand of work focuses on micro and contextual influences, exploring how certain individual characteristics shape trust. From this perspective, Brandt, Wetherell, and Henry (2015) analyse the effect on trust of personal income inequality, education, employment, and age. In turn, Dinesen and Sønderskov (2015) highlight the importance of exposure to the local environment for generating trust, while other authors examine the role of religiosity and community networks in the formation of trust ( Welch, Sikkink, and Loveland, 2007). Taken together, these studies reveal that education, personal economic prosperity, and positive social interactions are associated with higher levels of trust, whereas inequality and social isolation tend to erode it.

In general terms, the research does not simultaneously examine generalized and particularized trust and uses a fairly limited number of variables ( Delhey and Newton, 2005; Freitag and Traunmüller, 2009). Likewise, the studies do not sufficiently disaggregate the different forms of trust into subgroups of relationships (family members, neighbors, acquaintances) ( Glatz and Schwerdtfeger, 2022).

In this context, the present study proposes a broader synthesis that focuses specifically on generalized trust, drawing on a wide range of variables that combine macro-structural and individual-level factors. This integrated perspective makes it possible to assess how economic conditions, institutional quality, and personal orientations jointly shape trust toward strangers and socially heterogeneous others. In doing so, it becomes possible to assess whether the same determinants that strengthen generalized trust also operate in more im- mediate interpersonal contexts. Thus, this study extends the scope of previous research by offering a more complete, multivariable, and multiscalar perspective on the genesis of trust in contemporary societies.

## 3. Methodology

### 3.1 Data

In order to test the model, this research retrieves data from the World Values Survey ( Inglehart et al., 2014) for the wave covering the years 2018 to 2022. This survey is an international research program that analyses the social, political, economic, religious, and cultural values of people around the world. It covers a broad range of topics from the fields of Sociology, Political Science, International Relations, Economics, Public Health, Demography, Anthropology, and Social Psychology. The survey also includes macro-level country variables obtained from World Bank data, as well as individual-level information based on respondents’ answers to various questions. The total number of respondents was 97,200. The distribution by country is shown in Table 1.

**Table 1.** Sample distribution by country.

Country	Freq.	%	Cum.	Country	Freq.	%	Cum.
AND	1,004	1.03	1.03	MAC	1,023	1.05	54.18
ARG	1,003	1.03	2.06	MAR	1,200	1.23	55.41
ARM	1,223	1.26	3.32	MDV	1,039	1.07	56.48
AUS	1,813	1.86	5.19	MEX	1,741	1.79	58.27
BGD	1,200	1.23	6.42	MMR	1,200	1.23	59.50
BOL	2,067	2.13	8.55	MNG	1,638	1.68	61.19
BRA	1,762	1.81	10.36	MYS	1,313	1.35	62.54
CAN	4,018	4.13	14.49	NGA	1,237	1.27	63.81
CHL	1,000	1.03	15.52	NIC	1,200	1.23	65.05
CHN	3,036	3.12	18.64	NLD	2,145	2.21	67.71
COL	1,520	1.56	20.21	NZL	1,057	1.09	68.80
CYP	1,000	1.03	21.24	PAK	1,995	2.05	70.85
CZE	1,200	1.23	22.47	PER	1,400	1.44	72.29
DEU	1,528	1.57	24.04	PHL	1,200	1.23	73.53
ECU	1,200	1.23	25.28	PRI	1,127	1.16	74.69
EGY	1,200	1.23	26.51	ROU	1,257	1.29	75.98
ETH	1,230	1.27	27.78	RUS	1,810	1.86	77.84
GBR	2,609	2.68	30.46	SGP	2,012	2.07	79.91
GRC	1,200	1.23	31.69	SRB	1,046	1.08	80.99
GTM	1,229	1.26	32.96	SVK	1,200	1.23	82.22
HKG	2,075	2.13	35.09	THA	1,500	1.54	83.76
IDN	3,200	3.29	38.38	TJK	1,200	1.23	85.00
IND	1,692	1.74	40.12	TUN	1,208	1.24	86.24
IRN	1,499	1.54	41.67	TUR	2,415	2.48	88.72
IRQ	1,200	1.23	42.90	TWN	1,223	1.26	89.98
JOR	1,203	1.24	44.14	UKR	1,289	1.33	91.31
JPN	1,353	1.39	45.53	URY	1,000	1.03	92.34
KAZ	1,276	1.31	46.84	USA	2,596	2.67	95.01
KEN	1,266	1.30	48.14	UZB	1,250	1.29	96.29
KGZ	1,200	1.23	49.38	VEN	1,190	1.22	97.52
KOR	1,245	1.28	50.66	VNM	1,200	1.23	98.75
LBN	1,200	1.23	51.89	ZWE	1,215	1.25	100.00
LBY	1,196	1.23	53.12				

This research adopts a novel approach. Although many studies using data from the World Values Survey (WVS) measure trust with question v23 - *“Do you generally feel that most people can be trusted, or do you need to be very careful?”* - which yields a binary response, this study uses the question v128: “ *How much do you trust people you meet for the first time?*’. This latter question more directly captures the concept of generalized trust, understood as trust in strangers, and offers a four-point scale ranging from “trust completely” to “do not trust at all.” Furthermore, this paper tests the model over various types of trust and analyse the marginal effects of the variables on each of them.

### 3.2 Variables

**3.2.1 Dependent variables**

In methodological terms, the analysis begins with generalized trust (TMP), understood as an abstract disposition toward others as a whole. From there, it is particularized into trust in people one meets for the first time (TFT), which allows us to assess the extent to which the same determinants that sustain generalized trust are transferred to more concrete social interactions. Finally, the analysis includes the trust in neighbors (TN), as a general form of approximation to the trust between strangers but people who are closer.

To capture the concept of *trust*, we employed different variables. First, we created the variable *TMP* using valid answers to the question “Most people can be trusted”. If the answer to the question was “Most people can be trusted”, we coded it as 1, and 0 otherwise. In this way, we measure an overall perception of the concept. Since the concept admits a certain degree of adhesion that may appear at the initial moment of establishing contact, we created the variable *TFT* based on responses to question 61: “Do you trust people you meet for the first time?” We coded 4 if the respondent answered “Trust completely”, 3 for “Trust somewhat”, 2 for “Do not trust very much” and 1 for “Do not trust at all”. Missing data and “Don’t know” answers were not considered. We also created variables corresponding to trust in neighborhood ( *TN*) where we have coded the answers in the same way as for the variable *TFT.*

**3.2.2 Independent variables**

We created two sets of independent variables: one to capture elements that might affect trust at the country level, and another to capture factors that operate at the individual level.

*Country-level factors*

First, we considered the wealth of the country. To adjust it for population and scale, we created the variable *Wpc*, which represents the natural logarithm of GDP per capita. We also considered the distribution of wealth, for which we created the variable *Gini*, using the Gini Index as calculated by the World Bank. In the political domain, we used *IDn* to measure the degree of institutional development. To account for political stability, we created the variable *BTIstability*, based on the BTI stability index. To account for demographic density, we created the variable *Urb*, representing the percentage of the country’s population living in urban areas. And we have used *EduHDI* based on the Education Human Development Index of UN. We created the variable *MYS* to capture the average years of schooling. In addition, *RelFree* represents the country’s index of religious freedom. *MR* represents the national migration rate, and *LifeExp* indicates life expectancy.

*Individual factors*

We created several variables to capture factors that may influence trust at the individual level. To measure the personal importance of religion, we used the variable *RelImp*, which takes four possible values: 1 if religion is not at all important in life; 2 if not very important; 3 if rather important; and 4 if very important. The values were normalized using the Maxmin method. Similarly, we created *RelTol* to reflect tolerance toward other religions, using data obtained from the survey.

To measure educational attainment, we created the variable *EduLevN*, a transformed version of an 8-point scale ranging from no education to doctoral level. We also calculated its squared value to detect potential U-shaped effects. A similar transformation was applied to perceptions of democracy ( *DemPerN*).

Finally, we included the age ( *Age*) as an additional factor associated with different conceptions of trust, as well as the importance of self-perceived income level ( *ISPn*), based on a self-classification scale.

All data used in this study are publicly available and come from recognized institutional repositories, as described in the Source Data section.

The mapping of definitions, computations, and sources is shown in Table 2 and Table 3 shows the basic summary statistics.

**Table 2.** Variable definition.

Group	Variable	Definition	Computation	Source
Dependent	TMP TFT	Trust: most people Trust in the people you meet for the first time	(0 – No, 1 – Yes) (1 – Do not trust at all, 2 – Do not trust very much, 3 – Trust somewhat, 4 – Trust completely)	Survey Survey
	TN	Trust in the neighbor	(1 – Do not trust at all, 2 – Do not trust very much, 3 – Trust somewhat, – Trust completely)	Survey
Independent – Country	Wpc Gini IDn BTIstability MYS Urb EduHDI RelFree MR LifeExp EduLevN	Wealth per capita measured by GDP Gini Institutionalized democracy BTI stability of democratic institutions score Mean years of schooling Urban population rate Education Index Religious Freedom Migration rate Life expectancy at birth Educational level	LN of GDP per capita Gini coefficient Ln Institutional democracy BTI stability of democratic institutions score (1–10) Ln of mean years of schooling Urban population/Total population Education Human Development Index (0–1) Freedom of religion index (3–2) Net migration rate per 1,000 people Years Maxmin transformation of Educational level (highest) (0–8)	World Bank + own elaboration Survey + World Bank Survey+own elaboration Survey + Bertelsmann Survey+UNESCO+ own elaboration Survey + World Bank UNDP Survey World Bank Survey+own elaboration
Independent – Individual	EduLevNSQ DemPerN DemPerNSQ RelImp	Educational level (squared) Democratic perception score Democratic perception score (squared) Importance of religion in life	Maxmin transformation of Educational level (highest) (0–8) (squared) Maxmin transformation of democratic perception scores Maxmin transformation of democratic perception scores (squared) Maxmin transformation of scale on the question “important in life: religion”	Survey+own elaboration Survey+own elaboration Survey+own elaboration Survey+own elaboration
	RelTol Age ISPn	Tolerance to other religions Age Personal Income	Maxmin transformation of the inverse of the scale on the question “the only acceptable religion is my religion” Age Maxmin transformation of scale of income	Survey+own elaboration Survey Survey+own elaboration

**Table 3.** Basic summary statistics.

	N	Mean	SD	Min	Max
TMP	95883	0.24	0	0	1
TFT	95859	1.99	1	1	4
TN	96393	2.82	1	1	4
Wpc	91857	9.84	1	8	12
Gini	79976	37.13	6	25	54
IDn	89309	0.60	0	-0	1
BTIstability	71186	4.77	3	1	10
MYS	93400	0.61	0	0	1
Urb	95550	68.50	20	21	100
EduHDI	93400	0.72	0	0	1
RelFree	93619	0.50	1	-3	2
MR	92396	0.81	4	−22	23
LifeExp	94546	75.69	6	54	85
EduLevN	96149	0.45	0	0	1
EduLevNSQ	96149	0.26	0	0	1
DemPerN	95420	0.82	0	0	1
DemPerNSQ	95420	0.73	0	0	1
RelImp	96294	0.67	0	0	1
RelTol	89901	0.51	0	0	1
Age	95636	49.36	17	18	109
ISPn	94259	0.43	0	0	1

Source: Own elaboration from the Survey.

### 3.3 Model specification

To study the determinants of various types of trust, we employ both binary probit and ordered probit models, depending on the nature of the dependent variable. When the outcome variable is dichotomous, as in the case of generalized trust in most people ( *TMP*), we use a probit model. When the outcome variable is ordinal with more than two categories (e.g., trust in family, neighbors, or strangers), we apply an ordered probit specification.

**Binary Probit Model**

Let $y_{i}^{*}$ be the latent variable representing the unobserved propensity to trust:

$${y_{i}}^{*}=X_{i}\beta +\varepsilon_{i},\;\;\varepsilon_{i}∼N\;\left(0,1\right)$$

We observe the binary variable *y _i_* such that:

$$y_{i}=\left\{ \begin{array}{cc} 1 & \text{if}\;y_{i}^{*}>0\\ 0 & \text{otherwise} \end{array}\right.$$

The probability of trusting (i.e., y _i_ = 1) is then given by:

$$\mathrm{Pr}\;\left(y_{i}=1|X_{i}\right)=\Phi \;\left(X_{i}\beta \right)$$

where Φ (·) denotes the cumulative distribution function of the standard normal distribution.

**Ordered Probit Model**

For ordinal outcomes with four trust categories (1, 2, 3, 4), we define a latent variable *y _i_^∗^* as:

$${y_{i}}^{*}=X_{i}\beta +\varepsilon_{i},\varepsilon_{i}∼N\;\left(0,1\right)$$

and the observed variable *y _i_* is determined by threshold crossings:

$$y_{i}=\left\{ \begin{array}{cc} 1 & \text{if}\;y_{i}^{*}\leq \tau_{1}\\ 2 & \;\text{if}\;\tau_{1}<y_{i}^{*}\leq \tau_{2}\\ 3 & \text{if}\;\tau_{2}<y_{i}^{*}\leq \tau_{3}\\ 4 & \text{if}\;y_{i}^{*}\leq \tau_{3} \end{array}\right.$$

where τ1 < τ2 < τ3 are threshold parameters to be estimated.

In both models, the estimated coefficients β indicate the direction of the relationship between each predictor and the outcome, but they do not convey the magnitude of the effect in probability terms. For this reason, we compute and report average marginal effects (AMEs), which quantify the change in predicted probability associated with a one-unit change in each explanatory variable. This step is essential for the meaningful interpretation of substantive effects, especially in nonlinear models such as the probit and ordered probit. In probit models, the coefficients cannot be directly interpreted as changes in probability because they represent changes in the latent (unobserved) variable underlying the binary outcome. Therefore, marginal effects, usually with particularly average marginal effects (AMEs), are computed to provide a more intuitive interpretation. These indicate how a one-unit change in a predictor affects the probability of the outcome, holding other variables constant.

## 4. Results and discussion

We run the model for each of the dependent variables to test our hypothesis. Table 4 summarizes the results of the model and presents a comparative overview of the impact of the factors across the various probit and ordered probit models, each corresponding to a different dimension of trust. This synthesis allows to identify consistent predictors across trust types, as well as dimensions that are particularly sensitive to specific socioeconomic, institutional, or cultural factors.

**Table 4.** Models summary.

Model variables	(1) TMP	(2) TFT	(3) TN
Wpc Gini	0.248 *** (0.023) −0.029 *** (0.001)	0.164 *** (0.016) −0.025 *** (0.001)	0.147 *** (0.016) −0.032 *** (0.001)
IDn	0.079 **	0.032	−0.323 ***
BTIstability MYS Urb	(0.040) −0.026 *** (0.006) −0.878 *** (0.160) −0.007 *** (0.001)	(0.028) −0.011 *** (0.004) −1.779 *** (0.114) −0.005 *** (0.000)	(0.028) 0.021 *** (0.004) −0.304 *** (0.113) −0.005 *** (0.000)
EduHDI	0.864 ***	2.013 ***	−0.011
RelFree MR LifeExp EduLevN EduLevNSQ	(0.323) −0.156 *** (0.007) 0.009 ** (0.004) 0.022 *** (0.003) −0.332 *** (0.103) 0.580 *** (0.105)	(0.228) 0.023 *** (0.005) 0.009 *** (0.003) −0.014 *** (0.002) −0.456 *** (0.074) 0.601 *** (0.077)	(0.226) −0.039 *** (0.005) 0.021 *** (0.003) −0.020 *** (0.002) −0.459 *** (0.073) 0.458 *** (0.076)
DemPerN	0.129	0.273 ***	0.084
	(0.122)	(0.086)	(0.084)
DemPerNSQ	−0.205 **	−0.332 ***	0.018
RelImp RelTol Age ISPn	(0.093) −0.487 *** (0.023) 0.191 *** (0.022) 0.001 ** (0.000) 0.201 *** (0.031)	(0.066) −0.082 *** (0.017) 0.041 *** (0.016) 0.003 *** (0.000) 0.191 *** (0.022)	(0.065) 0.126 *** (0.017) 0.040 ** (0.016) 0.006 *** (0.000) 0.051 ** (0.021)
Observations	53,291	53,452	53,578

Standard errors in parentheses.

Dependent variables: TMP = Trust in most people; TFT = Trust in people you meet for the first time; TN = Trust in Neighbor.

Independent variables: Wpc: Log of GDP per capita; Gini: GINI elaborated by the WB; IDn: Institutional development index; BTIstability: institutional stability; MYS: mean years of schooling; Urb: proportion of urban population; EduHDI: Human Development Index Education; RelFree: index of religious freedom; MR: migration rate; LifeExp: life expectancy; EduLevN: level of in- dividual education; EduLevNSQ: level of individual education squared; Dem- PerN: level of demo perception; DemPerNSQ: level of demo perception squared; RelImp: individual perception of religion; RelTol: individual level of tolerance; Age: age; ISPn: income level.

*** p *<* 0.01,

** p *<* 0.05,

* p *<* 0.1.

Model 1 (as shown in Table 4) displays the results of the probit regression when the dependent variable is the response to the question “Most people can be trusted” (TMP). Model 2 and Model 3 (also shown in Table 4) present the results of ordinal regressions. The second column (Model 2 TFT) shows the results of trust in people met for the first time (TFT). The third column (Model 3 TN) refers to trust in neighbors (TN). We split the analysis into two major groups of variables: those representing country traits and those representing individual characteristics.

For the interpretation of these initial results, we consider that, in addition to the significance level of each predictor, the sign of the coefficients indicates the direction of the relationship between variables, but not the magnitude of the effect. Therefore, the data marked in the text is intended to reference the variable in the table, not to indicate the magnitude of the effect.

The statistical significance levels indicated by the asterisks in Table 4 highlight which associations are robust within each model, while the direction of the coefficients reveals whether those relationships are positive or negative. However, given the non-linear nature of the probit and ordered probit estimations, the actual magnitude of these effects will be more accurately captured through the marginal effects presented in the following section.

Table 4 summarizes the regression results for the different trust dimensions analysed. The discussion below highlights the most statistically robust associations, focusing on the direction and consistency of effects rather than the numerical magnitude of coefficients, as the latter will be further examined through marginal effects.

Certain variables exhibit a robust and homogeneous influence across all facets of trust. Per capita GDP (Wpc) is consistently and highly significantly associated with greater trust across all domains, ranging from the most general form TMP (0.248***), to trust in people met for the first time TFT (0.164***) and trust in neighbors TN (0.147***). This finding is consistent with the approach linking material prosperity to increased social capital ( Bjørnskov, 2017; Putnam, 2000). Conversely, the Gini index, as an indicator of economic inequality, shows a negative and highly significant association with generalized trust (−0.029***). This negative effect also appears in all other forms of trust (−0,025*** in TFT and − 0.032*** in TN), suggesting that inequality weakens social cohesion and generates dis- trust at almost all levels ( Knack, 2021; Rothstein and Uslaner, 2005; Uslaner, 2002). Similarly, the proportion of urban population (Urb) demonstrates a consistently negative and highly significant effect across all dimensions of generalized trust. Increased urbanization is linked to lower generalized trust (−0.007***), with this pattern replicating across all other forms of generalized trust (−0,005*** in TFT and − 0,005*** in TN). This result is supported by evidence that anonymity increases with city growth, reducing social ties and interpersonal trust.

At the individual level, we find that the so-called tolerance level (RelTol) correlates positively and highly significantly with almost all forms of generalized trust (0.191*** in TMP, 0,041*** in TFT and 0,040** in TN). This finding is intuitive, as tolerance is a value that fosters interaction, facilitating trust in diverse and pluralistic societies.

The same applies to age (Age), which also shows a positive effect on generating generalized trust (0.001** in TMP, 0,003*** in TFT and 0,006*** in TN), suggesting that the accumulation of life experiences promotes greater trust. Related to this, life expectancy (LifeExp) also shows a strong and significant positive association with generalized trust TMP (0.022***). However, the sign reverses for the other two forms of trust (−0.014*** in TFT and − 0.020*** in TN), suggesting that in contexts of greater longevity, the perception of vulnerability may foster increased caution and a reduced willingness to trust in broader social circles or in more immediate, everyday interactions ( Coleman, 1990). Although both variables relate to aging processes, they capture distinct mechanisms: age reflects an individual-level experiential trajectory that consistently increases trust, whereas life expectancy operates at the macro level, signalling welfare-state environments and population aging that may strengthen abstract beliefs about societal trustworthiness (TMP) while simultaneously reducing reliance on interpersonal interactions in everyday contexts, thereby lowering situational trust (TFT, TN).

Income level (ISPn) is also consistently and positively associated with generalized trust (0.201***) and with other trust models (0,191*** in TFT and 0,051** in TN). This result aligns with the idea that individuals with greater resources possess a sense of security that allows them to increase their trust in others ( Delhey and Newton, 2005; Putnam, 2000), feeling protected.

From this point, we find variables that have a more heterogeneous influence when considering the different forms of trust analysed, as we have done. Firstly, in the educational sphere, a country’s educational development (EduHDI) has a positive and significant relationship with generalized trust (0.864***) and with other forms of trust. However, while this occurs at a general level ( Green and Nelson, 2019), the micro-level analysis focused on the number of years of schooling (MYS) is negatively and highly significantly associated with most forms of trust, including generalized trust (−0.878***). This result can be explained by understanding that more years of formal education can foster critical thinking, which tends to reduce naive public trust ( Paxton, 1999), particularly when educational expansion is not accompanied by other factors such as social and institutional development ( Huang, Chen, and Chan, 2023). Nevertheless, these large negative coefficients should be interpreted with care: mean years of schooling may reflect cohort composition, selection effects, or unobserved heterogeneity across countries. The marginal effects confirm that the substantive impact is more moderate and reflects shifts across trust categories rather than a linear decline in trust. At the individual level, both the linear and squared terms of education (EduLevN and EduLevNSQ) show the expected convex relationship: trust first decreases and then increases with higher educational attainment. This pattern, consistent with prior research, suggests that education initially promotes critical distance but eventually fosters civic engagement and generalized trust among more highly educated individuals.

The institutional development index (IDn) exhibits a complex relationship. It is positive for generalized trust TMP (0.079**) but negative and highly significant for trust in neighbors TN (−0.323***). This pattern suggests a substitution effect whereby stronger formal institutions reinforce abstract, society-wide trust while reducing the reliance on localized interpersonal trust. As institutions become more robust, individuals may depend less on informal neighborhood-based networks and more on institutional guarantees, shifting trust upward from proximate interactions toward broader, impersonal expectations ( Coleman, 1990). In this way, solid and transparent institutions promote generalized trust, even if they attenuate trust in closer social circles.

Institutional stability (BTIstability), for its part, shows a heterogeneous pattern. It displays a negative and highly significant effect for TMP and TFT, yet a small positive effect for trust in neighbors (0.021***). This suggests that institutional stability does not uniformly foster generalized trust. Stability may reinforce perceptions of order, but when it reflects institutional immobility, often linked to low accountability, limited openness, or entrenched governance structures, it can hinder the development of generalized interpersonal trust. In such contexts, stability signals rigidity rather than institutional quality, thereby reducing trust in broader or more immediate social interactions. Thus, not all forms of stability promote trust; their effect depends on whether institutions are experienced as inclusive and responsive or as closed and inflexible.

The results reveal a nuanced pattern for religion-related variables. Religious freedom at the country level (RelFree) is strongly and negatively associated with TMP (−0.156***) and TN (−0.039***), while it shows a small but statistically significant positive effect on TFT (0.023***). Likewise, the individual importance attributed to religion (RelImp) is strongly and negatively related to TMP (−0.487***) and TFT (−0.082***), while it exhibits a positive and significant association with TN (0.126***). Although these variables operate through distinct mechanisms, their combined effects suggest that in societies where spiritual or moral frameworks are more salient, individuals tend to apply stricter normative criteria when assessing the trustworthiness of others. This tends to limit the extension of generalized trust both in abstract form (TMP) and in socially proximate contexts (TN), even if greater religious freedom may slightly facilitate openness in first-encounter situations (TFT) ( Smidt, 2018; Zmerli and Newton, 2008). By contrast, religious tolerance (RelTol) displays a positive and highly significant effect across all forms of generalized trust (0.191*** in TMP, 0.041*** in TFT, and 0.040** in TN), indicating that inclusive moral orientations promote openness toward others even in unfamiliar contexts. Overall, these results suggest that the influence of religion on generalized trust depends less on religiosity per se and more on whether spiritual norms narrow or broaden the evaluative scope through which individuals interpret the social world.

A similar pattern arises with perceptions of democracy. The squared term (DemPerNSQ) shows a negative and significant association with generalized trust (−0.205**), indicating that extreme evaluations, either highly positive or highly negative, may erode generalized expectations of trustworthiness. This result aligns with the idea that unmet normative expectations about democratic performance can weaken diffuse interpersonal trust ( Dalton, 2020; Putnam, 2000). By contrast, the linear component (DemPerN) shows only a weak pattern: it is not significant for TMP or TN, and although positive for TFT (0.273***), its substantive contribution remains limited. Overall, only the non-linear component appears relevant for understanding how democratic perceptions shape generalized trust.

Finally, the migration rate (MR) exhibits mixed results, showing a small but positive association with generalized trust (0.009**). This suggests that societies with higher migration flows may not necessarily experience declines in trust and, in some cases, may even foster attitudes of openness toward others.

In conclusion, we highlight that, although complex patterns appear at some points, it seems clear that socioeconomic, institutional, and individual factors intervene in the generation of generalized trust. This suggests that a multi-causal approach should be undertaken when studying the inducers that promote different types of trust.

## 5. Marginal effects

As noted in the methodology section, the previously estimated coefficients provided valuable insights into the direction and significance of relationships between variables. However, these coefficients do not directly convey the magnitude of effects, a crucial aspect for a comprehensive understanding of substantive relationships, particularly in non-linear models such as probit and ordered probit.

To address this limitation, we turn to the analysis of Average Marginal Effects (AMEs). AMEs quantify the change in predicted probability associated with a one-unit change in each explanatory variable, holding all other variables at their observed values. This allows for a more meaningful interpretation of substantive effects, complementing the directional insights obtained from coefficient estimates.

While Table 4 reports a single coefficient per predictor for each ordered probit model, capturing the average directional association across the full distribution of the dependent variable, the AME-based graphical analysis disaggregates these effects by trust level. Ordered outcomes such as TFT and TN require marginal effects for meaningful interpretation, since coefficients cannot be read as probabilities nor as marginal changes. As a result, the magnitude, sign, and statistical significance observed in the global model may not be replicated uniformly across all categories.

In particular, a variable with a significant overall coefficient may display positive marginal effects in some trust levels and negative or insignificant effects in others, reflecting the ordered model’s nature as a weighted average across thresholds. Conversely, small or non-significant AMEs at each category may still yield a significant global coefficient if the effects, though modest, are consistent in direction across levels. Thus, the two approaches are complementary: ordered probit coefficients summarize general tendencies, whereas disaggregated AMEs reveal how these tendencies vary across specific levels of trust.

In the AME framework, a positive value indicates that an increase in the explanatory variable raises the probability of being in a specific trust level, holding other variables constant. Positive AMEs at low trust levels suggest that the variable is associated with reduced trust, whereas positive effects at high trust levels indicate higher trust; negative AMEs im- ply the opposite. The pattern of signs and magnitudes across the response scale allows us to identify variables that strengthen or weaken trust (see Table 5). Detailed quantitative results for each trust type and level-specific AMEs are provided in the appendices available in the repository cited at the end of the manuscript.

**Table 5.** General interpretation of AME patterns.

Sign pattern	Interpretation
Positive in low levels, negative in high levels	Factor reducing trust
Negative in low levels, positive in high levels	Factor increasing trust
Positive only in intermediate levels	Associated with moderate trust
Negative only in intermediate levels	Shifts towards extremes (high or low trust)
Low or near-zero signs across all levels	No substantive effect

The following subsections present graphical summaries of the main results for each type of trust analysed, displaying the effects of all explanatory variables. These results are then examined in detail. Level-specific interpretations of the *average marginal effects* (AMEs) for each trust dimension are provided in the appendices available in the repository cited at the end of the manuscript. Generalized trust, being measured as a binary outcome, is presented with a distinct graphical style from the other trust types.

### 5.1 Marginal effects on most people can be trusted (TMP)

**Figure 1.**
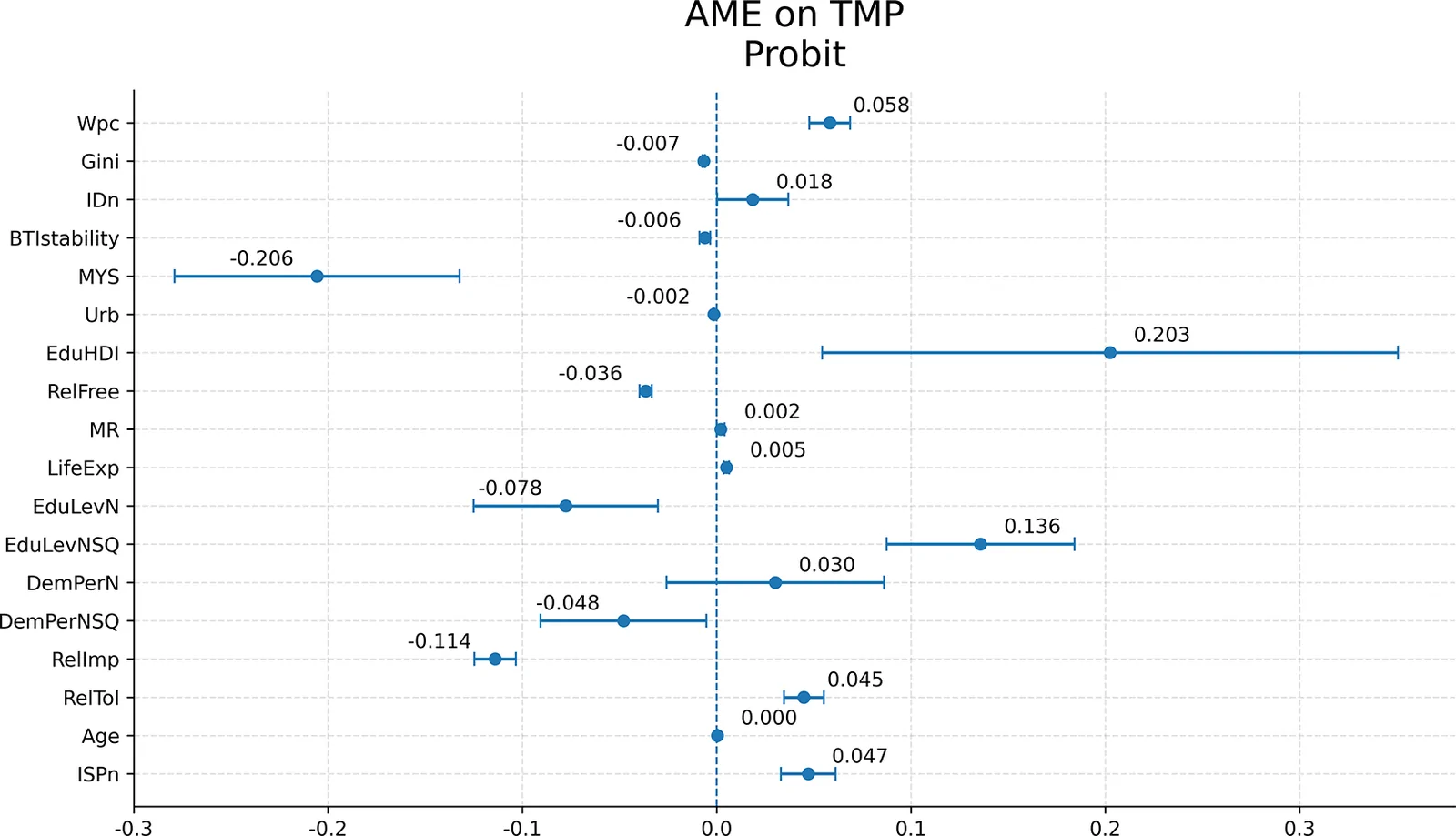
Average marginal effects on TMP trust.

This section examines the individual- and country-level drivers of generalized trust, measured through the probability of agreeing with the statement “Most people can be trusted” (TMP = 1; otherwise, 0). The analysis is based on a probit model, and the reported coefficients correspond to AMEs, indicating how one-unit changes in each predictor alter the likelihood of expressing generalized trust. Figure 1 presents the AMEs for TMP across all variables considered.

*Country-level factors*

The Average Marginal Effects (AMEs) for TMP are disaggregated in Appendix B available in the repository cited at the end of the manuscript. The main findings are as follows.

*Wealth per capita (Wpc) shows a positive and highly significant effect (+5.8 p.p., p < 0.01), consistent with the notion that economic prosperity supports generalized trust. Income inequality (Gini) exhibits a significant negative effect (−0.67 p.p., p < 0.01), reinforcing the idea that inequality undermines social cohesion.*

Institutional variables display mixed patterns. Institutionalized democracy (IDn) has a positive and statistically significant effect (+1 *.*85 p.p., *p <* 0 *.*05), while institutional stability (BTIstability) shows a significant negative effect (−0 *.*61 p.p., *p <* 0 *.*01), suggesting that stability perceived as institutional rigidity may weaken generalized trust. These AMEs closely mirror the patterns in Table 4.

Education-related indicators also diverge. Mean years of schooling (MYS) shows a large negative effect (−20 *.*6 p.p., *p <* 0 *.*01), whereas the Educational Human Development Index (EduHDI) exhibits an equally large positive effect (+20 *.*2 p.p., *p <* 0 *.*01). This supports the idea that national educational development promotes trust, while individual schooling may initially foster critical distance rather than naive trust.

Urbanization (Urb) has a significant negative impact (−0 *.*15 p.p., *p <* 0 *.*01), likely reflecting weaker informal ties and greater anonymity in urban contexts.

*Individual-level and cultural factors*

Educational attainment (EduLevN) and its squared term (EduLevNSQ) display the expected U-shaped pattern: the linear coefficient is negative (−7.77 p.p., p < 0.01) and the quadratic term is positive (+13.58 p.p., p < 0.01). Trust declines at lower and intermediate education levels but rises at higher levels, where education may promote civic engagement. Religious freedom (RelFree) has a negative marginal effect on TMP (−3.65 p.p., p < 0.01). Greater religious pluralism may expose individuals to a wider array of normative frameworks, encouraging more cautious assessments of anonymous others and thereby reducing generalized trust.

Life expectancy (LifeExp), by contrast, has a positive and significant effect (+0.50 p.p., p < 0.01). Longer-lived societies, often associated with stronger welfare systems and more stable living conditions, tend to foster more optimistic abstract beliefs about the trust worthiness of others.

Perceptions of democracy also shape generalized trust. The linear component of democratic perception (DemPerN) is not significant, while the squared term (DemPerNSQ) is negative and significant (−4 *.*8 p.p., *p <* 0 *.*05). This indicates a non-linear pattern: extreme evaluations of democracy, either highly positive or highly negative, correspond to lower generalized trust, whereas moderate assessments are associated with higher trust.

At the individual level, the importance attributed to religion (RelImp) shows a strong negative effect (−11 *.*4 p.p., *p <* 0 *.*01), while religious tolerance (RelTol) has a positive and significant effect (+4 *.*48 p.p., *p <* 0 *.*01). Thus, stronger personal religiosity appears to reduce generalized trust, whereas inclusive moral orientations tend to strengthen it.

Socio-demographic and economic controls behave as expected. Age has a very small but positive effect (+0 *.*03 p.p., *p <* 0 *.*05), consistent with the notion that accumulated life experience modestly increases trust. Personal income (ISPn) shows a positive effect (+4 *.*71 p.p., *p <* 0 *.*01), with a stronger impact at higher trust levels, reinforcing the idea that financial security facilitates interpersonal trust.

While generalized trust (TMP) reflects an abstract belief in the reliability of others, it remains somewhat detached from concrete social interactions. To capture this dimension more precisely, the next subsection analyses trust in people one meets for the first time (TFT). This variable can be interpreted as a behavioural counterpart of generalized trust in everyday encounters, allowing us to assess whether the same mechanisms that sustain abstract trust also operate in unfamiliar, situational interactions.

### 5.2 From Generalized Trust (TMP) to Trust in People You Meet for the First Time (TFT)

While generalized trust (TMP) provides an abstract measure of confidence in others, it remains to some extent detached from concrete social interactions. To capture this dimension more clearly, without losing the general character of trust in others, we extend the analysis to the specific case of Trust in People You Meet for the First Time (TFT). This form of trust can be considered a more concrete application of generalized trust, as it reflects how individuals approach Trust in People You Meet for the First Time (TFT) in their everyday lives, based on their broader idea of trust in others. Later on, as mentioned, we extend this analysis to closer forms of trust such as trust in neighbors.

Before presenting the results, it is worth noting that for several variables the average marginal effects (AMEs) across the four trust levels (TFT = 1–4) are very close to zero. In such cases, we refrain from establishing direct comparisons with the aggregate coefficients in Table 4, since the near-zero AMEs indicate that the estimated global effect likely reflects small and distributed changes rather than a consistent directional pattern at any specific level. References to Table 4 are therefore made only when the level-based results display a clear and coherent sign consistent with the overall model.

In substantive terms, the comparison between TMP and TFT allows us to examine whether both types of trust respond to the same mechanisms, as would be expected. Some of these patterns of possible similarity were already detected in the general analysis of trust inducing factors shown in Table 4. In particular, TMP and TFT share a solid economic foundation: both increase with national wealth (Wpc, 0.248*** vs. 0.164***) and individual income (ISPn, 0.201** vs. 0.191***), while inequality marginally reduces them (Gini, −0.029*** vs. –0.025***. Nor are there notable differences in relation to educational factors: mean years of schooling significantly reduce both forms of trust (MYS, −0.878*** vs. –1.779***), while education as measured through the HDI (EduHDI) enhances both (0.172*** vs. 0.186***).

At the institutional level, institutional development increases TMP positively though moderately (IDn, 0.079**), while showing no significant effect on TFT (0.032). The clearest differences are found in life expectancy, which increases TMP (LifeExp, 0.022***) but reduces TFT (−0.014***), and in religious freedom, which decreases TMP (RelFree, −0.156***) but slightly increases TFT (+0.023***). Finally, democratic perceptions are of little relevance for TMP (DemPerN = 0.129; DemPerNSQ = −0.205**), but significant for TFT (0.273*** and − 0.332***, respectively).

Taken together, TMP and TFT display very similar patterns, but the study of marginal effects help to sharpen the analysis, allowing for a clearer understanding of the differences already observed in relation to life expectancy and religious freedom.

Therefore, analysing the marginal effects of TFT immediately after TMP enables us to refine the interpretation of generalized trust and to identify whether its determinants operate uniformly between abstract attitudes and situational behaviours.

**Figure 2.**
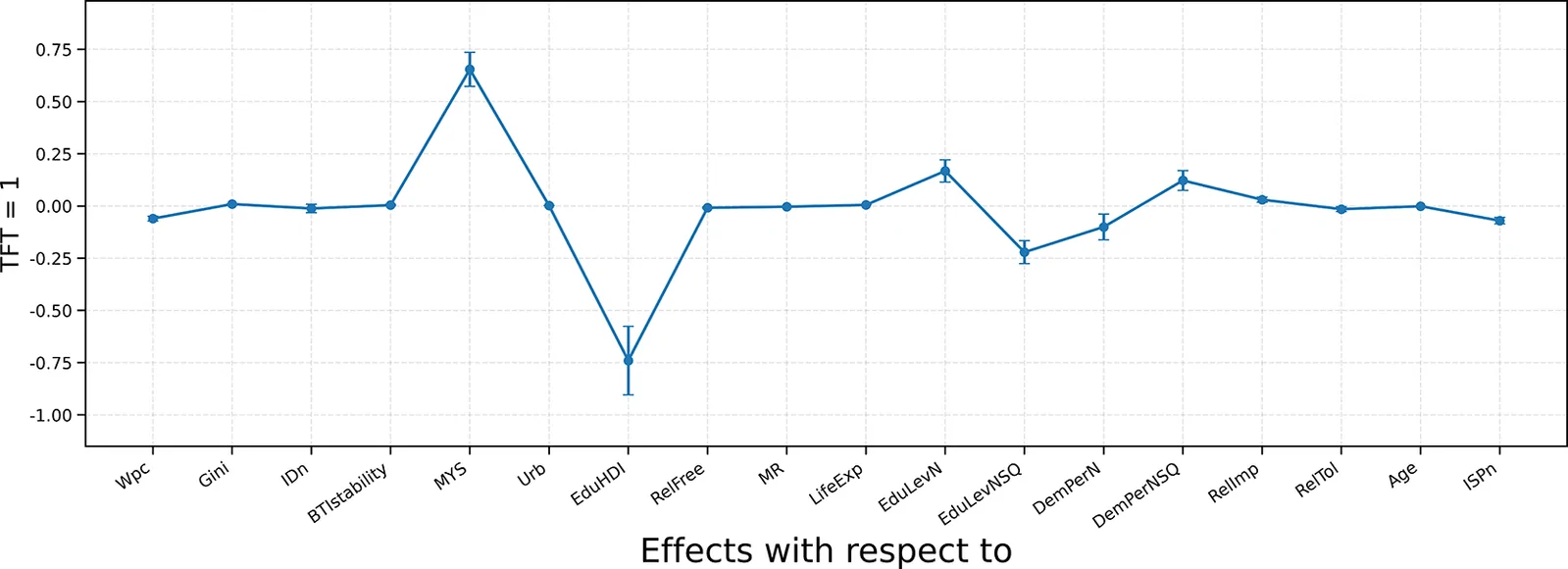
Average marginal effects on Trust in People You Meet for the First Time (TFT) TFT = 1.

**Figure 3.**
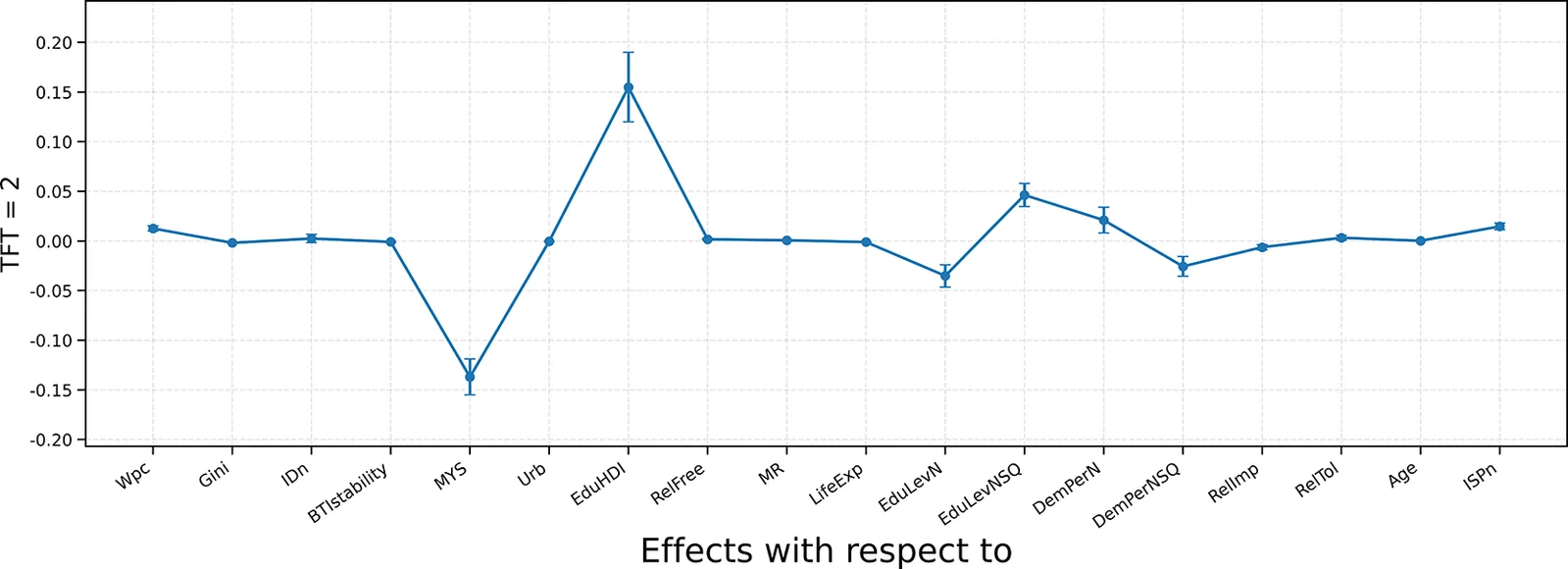
Average marginal effects on Trust in People You Meet for the First Time (TFT) TFT = 2.

**Figure 4.**
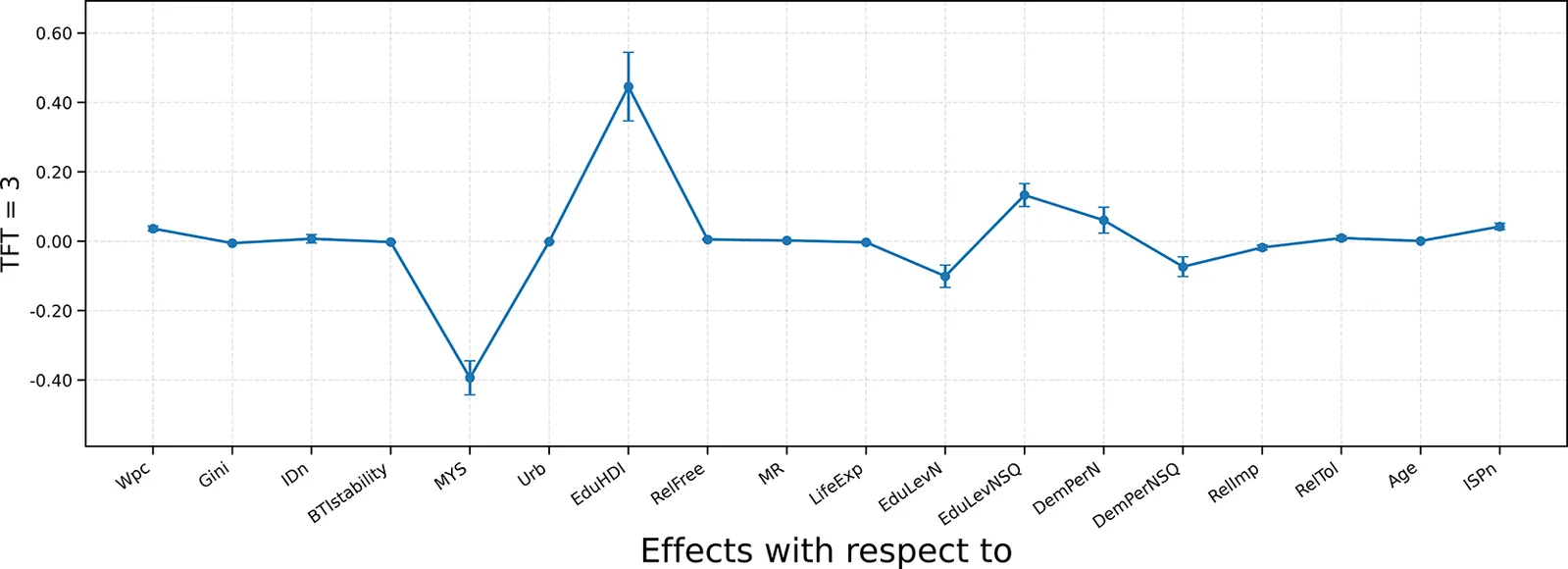
Average marginal effects on Trust in People You Meet for the First Time (TFT) TFT = 3.

**Figure 5.**
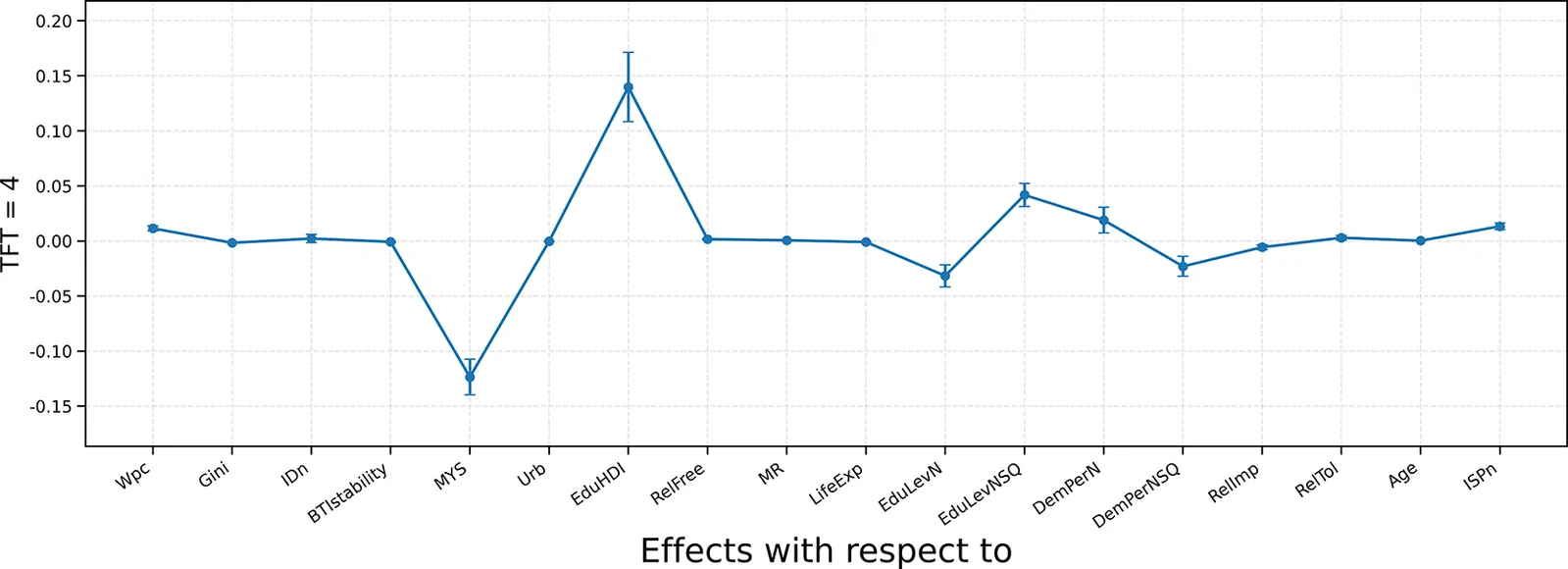
Average marginal effects on Trust in People You Meet for the First Time (TFT) TFT = 4.

We computed the average marginal effects (AMEs) derived from an ordered probit model predicting the level of Trust in People You Meet for the First Time (TFT), as summarized in Figures 2 to 5 for all variables included in the model.

This dependent variable is ordered from low (1) to high (4). For each explanatory variable, the AMEs represent the change in the probability of belonging to each trust level associated with a marginal change in the predictor, holding other variables constant.

*Country-level factors*

Regarding wealth per capita (Wpc), the pattern is clear: in the lowest trust levels (TFT = 1) the effect is negative and significant, while in the higher levels (TFT = 2, TFT = 3 and TFT = 4) it is positive. This finding, consistent with the positive sign in Table 4, indicates that greater economic prosperity reduces the probability of very low trust in Trust in People You Meet for the First Time (TFT) and simultaneously increases the probability of reporting moderate or high trust, suggesting that economic security fosters openness in Trust in People You Meet for the First Time (TFT). Income inequality (Gini) shows effects close to zero across the four levels, without a clear directional pattern. The small negative tendency in the higher levels (TFT = 3 and TFT = 4) is consistent with the overall negative association in Table 4, but the magnitude of these AMEs suggests that inequality affects TFT only marginally.

The role of mean years of schooling (MYS) departs from expectations. It is positive at the lowest level (TFT = 1) and negative in the higher ones (TFT = 2, TFT = 3 and TFT = 4). This counterintuitive result suggests that greater educational attainment at the country level may reduce reliance on generalized trust, possibly reflecting greater caution or selectivity in Trust in People You Meet for the First Time (TFT). This pattern mirrors the negative coefficient reported in Table 4.

In contrast, the Education Index (EduHDI) follows a pattern of reduction in the lowest level (TFT = 1) and increases in higher levels (TFT = 2, TFT = 3 and TFT = 4). This is consistent with the positive sign in Table 4, supporting the idea that national educational development fosters a more favourable context for openness in Trust in People You Meet for the First Time (TFT).

Institutionalized democracy (IDn) does not show significant effects across any level, which suggests that formal democratic institutions do not directly influence Trust in People You Meet for the First Time (TFT). Institutional stability (BTIstability) also displays effects close to zero in all levels, without a clear positive or negative pattern. Hence, although the aggregate coefficient in Table 4 is slightly negative, these near-zero AMEs indicate that stability alone has limited substantive impact on this form of trust.

The urbanization factor (Urb) presents effects near zero at all levels (TFT = 1 and TFT = 4). While the general model reports a negative sign, the marginal effects suggest that this association is weak and diffuse, with no systematic pattern by level.

Migration rate (MR) shows near-zero effects across all levels, implying that the positive aggregate coefficient in Table 4 likely results from small cumulative shifts rather than a distinct influence on any specific trust level.

Life expectancy displays AMEs extremely close to zero across all levels, with only a mild negative tendency in the higher categories (TFT = 3 and TFT = 4). Therefore, the negative coefficient in Table 4 likely reflects a very small average effect rather than a strong level specific pattern.

*Individual-level factors*

Education level (EduLevN) and its squared term (EduLevNSQ) show a U-shaped pattern, consistent with Table 4: the linear coefficient is positive in the lowest levels (TFT = 1 and TFT = 2) and negative in the higher ones (TFT = 3 and TFT = 4), while the squared term is negative in the lower levels and positive in the higher ones. This indicates that only at very high education levels is part of high trust recovered, while intermediate education tends to be associated with more cautious attitudes toward strangers.

Perception of democracy (DemPerN) is negative at the lower trust levels (TFT = 1 and TFT = 2) and positive at the higher ones (TFT = 3 and TFT = 4), indicating that optimistic democratic evaluations disproportionately support higher interpersonal trust. The quadratic term (DemPerNSQ) shows small, near-zero effects across levels. The overall pattern remains compatible with a curvilinear relationship between democratic perceptions and TFT, though the strength of this pattern is limited.

Religious freedom shows near-zero effects at TFT = 1, followed by consistently positive AMEs at TFT = 2 and TFT = 4, with the strongest value at TFT = 3. This suggests that freedom of belief may modestly favor openness in Trust in People You Meet for the First Time (TFT), although the magnitudes are small. Religious tolerance (RelTol) follows a similar profile, with small positive AMEs at higher levels, reinforcing the view that inclusive values support interpersonal openness.

Religious importance (RelImp) displays negative effects across all levels, more pronounced in the higher ones (TFT = 3 and TFT = 4). This indicates that strong personal religiosity tends to reduce openness in Trust in People You Meet for the First Time (TFT), consistent with the negative sign observed in Table 4.

Personal income (ISPn) exerts a robust and consistent pattern: it is strongly negative in the lowest level (TFT = 1) and positive in the higher levels (TFT = 2, TFT = 3 and TFT = 4), fully consistent with the positive coefficient in Table 4. This confirms that individual economic security fosters openness in Trust in People You Meet for the First Time (TFT).

Finally, age (Age) shows effects very close to zero across levels, with a slight positive tendency in higher categories. Hence, while the aggregate coefficient in Table 4 is positive, individual age does not substantially shift the distribution of trust toward strangers.

Taken together, the results show that for TFT, the most consistent level-based effects correspond to economic prosperity (Wpc, ISPn), educational development (EduHDI), and schooling structure (MYS, EduLevN, EduLevNSQ). These variables align well with the overall model in Table 4. In contrast, institutional, demographic, and religious context variables display weak or near-zero AMEs, suggesting that their aggregate associations mainly capture modest average shifts rather than substantive within-level variations. The pattern confirms that the mechanisms sustaining openness toward strangers are primarily economic and educational, whereas structural and demographic factors play a secondary, stabilizing role.

### 5.3 Marginal effects on Trust in Neighbors (TN)

This section examines the factors shaping trust in neighbors, a component of localized social capital that reflects expectations of cooperation within immediate social environments. The dependent variable (TN = 1–4) is modelled through an ordered probit, and the aver- age marginal effects (AMEs) represent how a one-unit change in each predictor alters the probability of belonging to each trust level.

**Figure 6.**
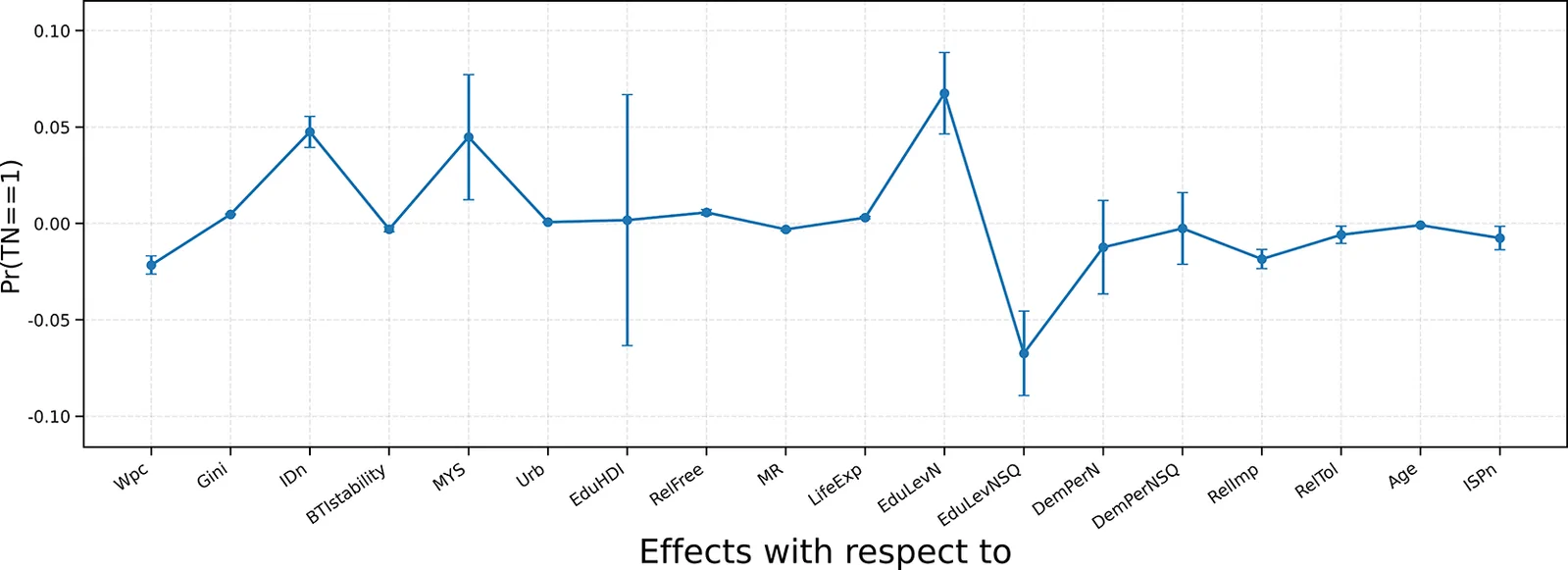
Average marginal effects on trust in neighbors (TN) TN = 1.

**Figure 7.**
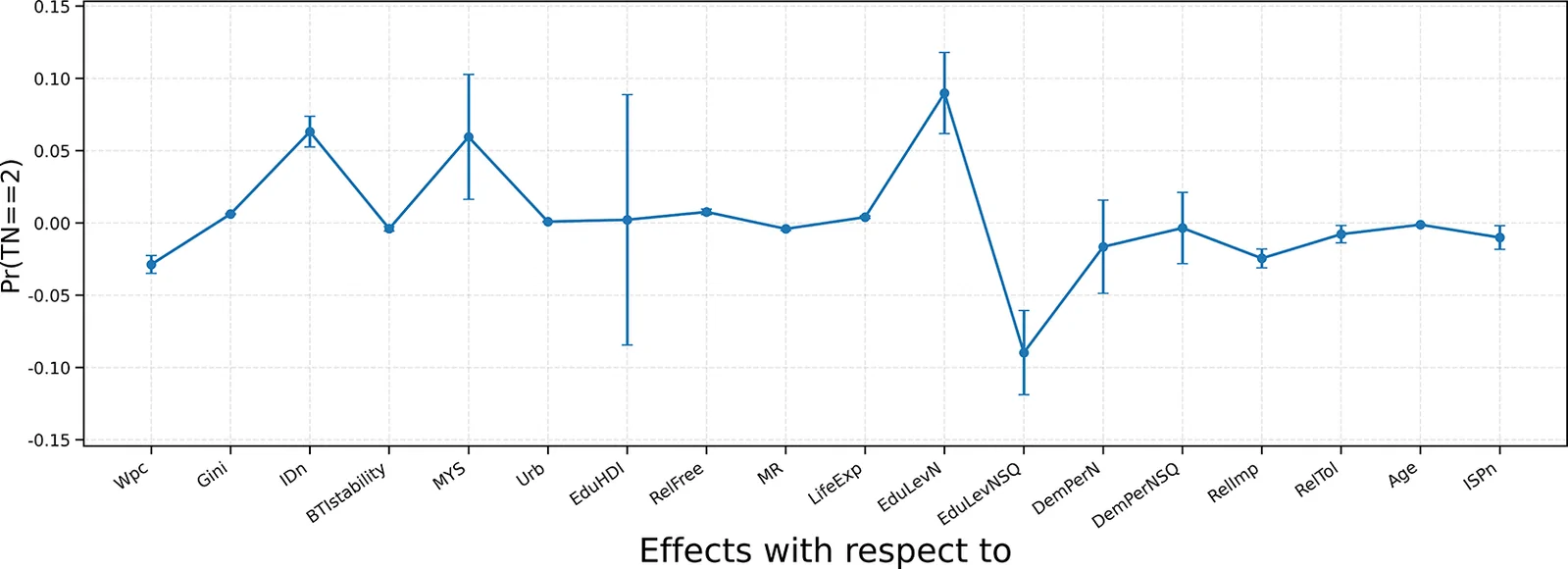
Average marginal effects on trust in neighbors (TN) TN = 2.

**Figure 8.**
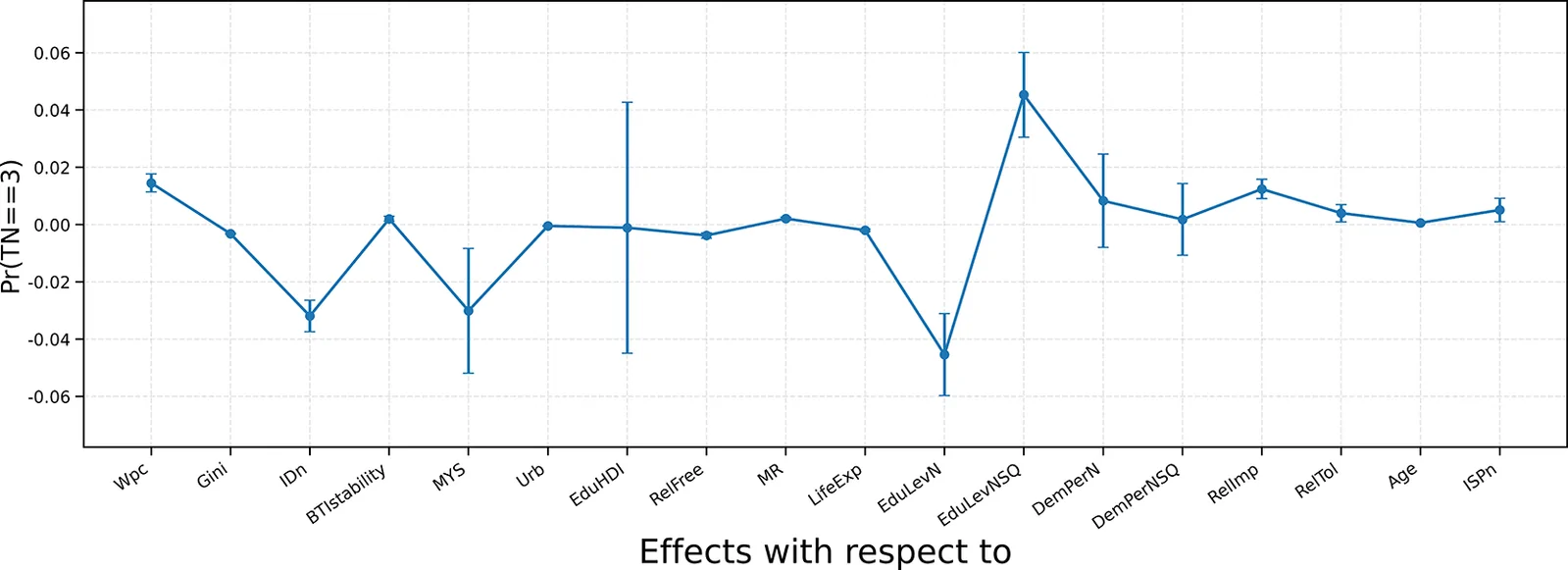
Average marginal effects on trust in neighbors (TN) TN = 3.

**Figure 9.**
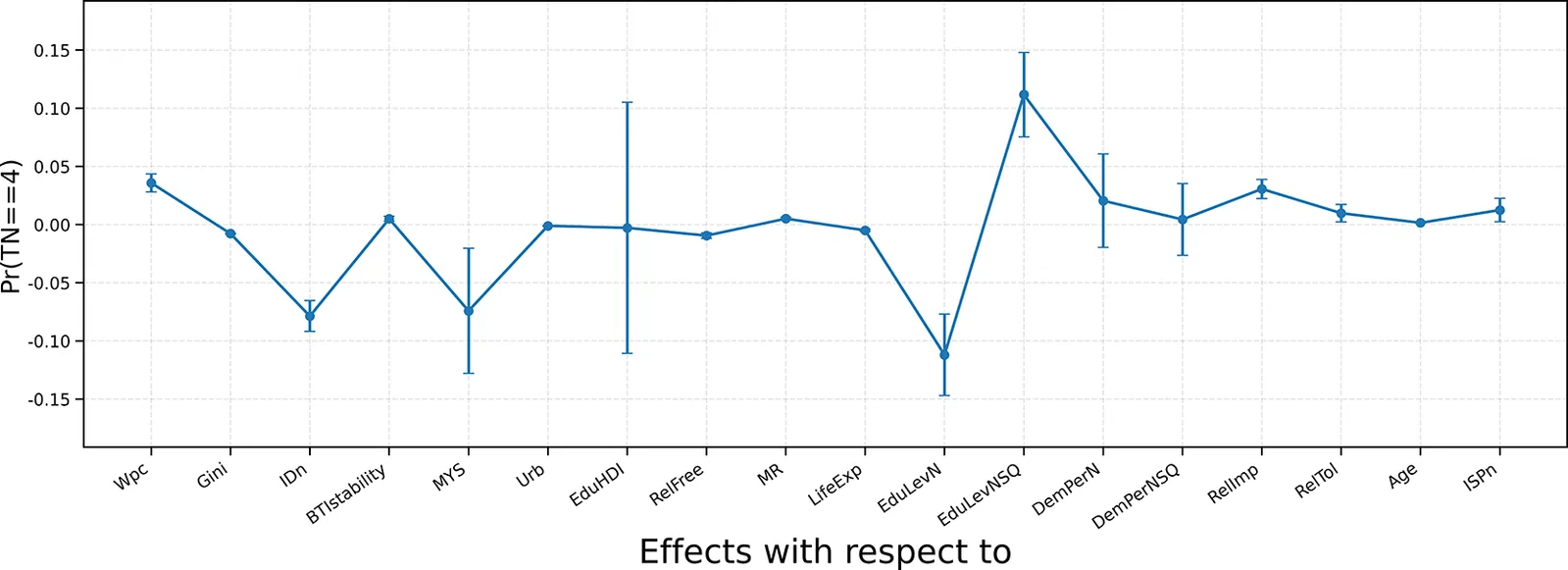
Average marginal effects on trust in neighbors (TN) TN = 4.

Figures 6 to 9 display the AMEs for all predictors, disaggregated by trust level. In variables where AMEs remain extremely close to zero across levels, we refrain from drawing substantive comparisons with the global coefficients in Table 4, since such cases indicate diffuse, low impact effects rather than meaningful shifts in specific categories.

*Country-level factors*

Wealth per capita (Wpc) shows a coherent pattern: AMEs are negative in the lowest trust levels (TN = 1 and TN = 2) and positive in the highest ones (TN = 3 and TN = 4). This matches the positive global coefficient in Table 4 and indicates that economic prosperity reduces very low trust while increasing high neighborhood trust.

Income inequality (Gini) exhibits small negative effects in TN = 3 and TN = 4, consistent with the negative coefficient in Table 4, though the level-specific magnitudes suggest that inequality’s influence is modest and concentrated at the top of the trust distribution.

Institutionalized democracy (IDn) presents slight positive AMEs in TN = 1 and TN = 2 and slight negative AMEs in TN = 3 and TN = 4. These effects are small and statistically weak, implying that formal democratic structures do not systematically shift trust in neighbors.

Institutional stability (BTIstability) yields AMEs very close to zero across TN = 1–4, with only mild sign variations. The slightly negative aggregate coefficient likely reflects small, diffuse average shifts rather than meaningful effects at any specific trust level.

Mean years of schooling (MYS) reproduces a consistent pattern: AMEs are positive in TN = 1 and TN = 2 and negative in TN = 3 and TN = 4. This aligns with the negative global sign and suggests that higher schooling tends to shift probability away from high trust neighborhoods, possibly reflecting broader social horizons that dilute localized ties.

The Educational Human Development Index (EduHDI) shows effects close to zero across TN = 1–4, consistent with its non-significant coefficient in Table 4. National educational development does not appear to translate into systematic differences in neighbor trust.

Urbanization (Urb) displays near-zero effects in TN = 1 and TN = 2, and a clearer negative effect in TN = 4. This aligns with the negative aggregate coefficient and supports the hypothesis that dense urban environments may erode high trust in neighbors without notably affecting lower trust levels.

Life expectancy (LifeExp) exhibits a crossed pattern: mildly positive AMEs in TN = 1 and TN = 2 and negative AMEs in TN = 3 and TN = 4. This explains the negative aggregate coefficient: longer national longevity corresponds to a reduced probability of high neighborhood trust, while exerting little influence at lower trust levels.

*Individual-level factors*

Education level (EduLevN) and its quadratic term (EduLevNSQ) again produce a clear U-shaped pattern: the linear term is positive at TN = 1 and TN = 2 and negative at TN = 3 and TN = 4, while the quadratic term reverses sign. This mirrors Table 4 and suggests that intermediate education reduces high neighborhood trust, whereas very high education partially restores it.

Democratic perceptions (DemPerN and DemPerNSQ) display AMEs very close to zero across TN = 1–4, echoing their non-significance in Table 4. Citizens’ subjective evaluations of democracy do not systematically influence trust in neighbors.

Religious freedom (RelFree) shows AMEs near zero in TN = 1 and TN = 2 and small positive AMEs in TN = 3 and TN = 4. Religious tolerance (RelTol) follows a similar profile, with small but positive effects in higher trust categories. Although magnitudes remain modest, these results are compatible with the idea that inclusive value climates favour interpersonal openness.

Religious importance (RelImp) shows negative AMEs in TN = 1 and TN = 2 and positive AMEs in TN = 3 and TN = 4, fully matching the positive and significant global coefficient in Table 4. This indicates that personal religiosity shifts probability away from low trust and toward higher neighborhood trust.

Personal income (ISPn) shows positive AMEs in TN = 3 and TN = 4 while remaining near zero in TN = 1 and TN = 2. This is consistent with the positive global coefficient and confirms that individual economic security strengthens higher neighborhood trust.

Age (Age) shows AMEs extremely close to zero across TN = 1–4. Although the global coefficient is slightly positive, age does not meaningfully shift the probability distribution across trust categories.

For this trust in neighbors, several variables exhibit clear, meaningful level-based effects, particularly wealth per capita, personal income, mean years of schooling, the structure of individual education, life expectancy, and religious importance. Others display weak or near-zero AMEs (Gini, IDn, BTIstability, Urb, MR, RelFree, RelTol, Age, EduHDI, DemPerN, DemPerNSQ), indicating that their global coefficients mainly reflect small aver- age shifts rather than substantive within-level changes. Neighborhood trust is thus driven principally by economic resources, educational structure, and certain cultural orientations, whereas institutional and demographic conditions exert secondary or negligible influence.

## 6. Conclusions and policy and corporate implications

This study provides a comprehensive analysis of the determinants of trust across multiple domains—from generalized trust (TMP) to trust in people met for the first time (TFT) and trust in neighbors (TN). By combining probit and ordered probit estimates with an analysis of average marginal effects (AMEs), we reveal both consistent regularities and domain- specific patterns. A central contribution of this work is the empirical differentiation between generalized, bridging, and localized trust: rather than reflecting a single underlying dimension, each responds to distinct economic, institutional, and personal mechanisms. Certain drivers—most notably economic prosperity and personal economic security—consistently strengthen trust across domains, whereas inequality and urban fragmentation weaken it. Other determinants, such as education, religiosity, and perceptions of democracy, operate in opposite directions depending on the trust domain, revealing compensatory and substitution effects that shape the structure of trust in modern societies.

Our results highlight that scenarios of economic stability, at both the country and individual level, are consistently associated with greater trust of all kinds. Tolerance, older age, and higher life expectancy contribute to environments in which generalized trust can flourish. Conversely, inequality and large, anonymous urban settings systematically erode trust. Localized trust of the type captured by TN is more resilient in some respects, particularly to demographic pressures, but remains sensitive to economic factors.

The analysis also uncovers heterogeneous and domain-specific influences. At the institutional level, a mild substitution effect is observed: institutional development strengthens generalized trust (TMP) but has little or no effect on trust in neighbors (TN). Institutional stability shows very small AMEs across domains, suggesting that its aggregate associations reflect diffuse, low-intensity shifts. Perceptions of democracy likewise have limited explanatory power for TFT and TN, though extreme evaluations reduce TMP.

Religious variables show domain-specific profiles. Personal religiosity reduces generalized trust and trust in strangers (TMP and TFT), but increases trust in neighbors (TN), reflecting a bonding rather than bridging effect. Religious freedom and tolerance, by contrast, display small but positive associations with higher trust categories, especially in TFT and TN. This suggests that inclusive moral environments broaden the trust horizon, even if the magnitude of these effects remains modest.

In the educational sphere, greater educational development at the country level boosts general trust, but more years of individual schooling are associated with critical and cautious behaviours regarding others. Country-level education (EduHDI) builds trust; individual schooling (MYS) induces cautiousness, reducing trust beyond close circles. This educational paradox highlights how cognitive sophistication and social mobility may dilute bonding ties while reinforcing bridging and generalized forms of trust. A notable contribution of this study is the clarification of what initially appears to be a counterintuitive pattern in the relation- ship between educational development and family trust. While the ordered probit coefficients suggest a strong negative association between national educational development (EduHDI) and the highest category of family trust, the marginal effects reveal a more nuanced reality: family trust does not decline. Rather, in more educationally developed societies, trust remains high but becomes less absolute, shifting from unconditional reliance toward intermediate, but still strong, levels of confidence. This pattern suggests that broader educational development reduces exclusive dependence on kinship networks in favour of more diversified social support structures, without eroding the underlying trust placed in family members.

Migration and urbanization influence trust selectively. Migration has small and inconsistent effects at the level of AMEs, indicating that it does not substantively shift trust distributions in the domains studied. Urbanization reduces the probability of high neighborhood trust (TN), consistent with the idea that dense environments weaken localized social bonds. Over time, however, integration policies may attenuate these effects.

Overall, trust emerges not as a cultural residue or a psychological trait, but as a multicausal construct shaped simultaneously by economic, institutional, and personal factors. Policies aimed at strengthening trust must therefore address each of these layers, attending to both structural conditions and individual orientations. Promoting trust requires aligning social and economic initiatives with principles of fairness, inclusion, tolerance, and mutual recognition.

### 6.1 Public policy priorities

Based on the results obtained, several guidelines can be proposed to support public policies aimed at fostering more cohesive and trusting societies. First, policymakers must recognize that trust is multidimensional and multifactorial: it is shaped by aggregated economic conditions, institutional frameworks, and personal attributes. Consequently, interventions should not remain confined to the macroeconomic or institutional spheres, but must also create spaces for personal development, civic inclusion, and intercultural interaction.

Economic development, both at the national and individual scale, is essential. Efforts to promote inclusive growth and reduce inequality are central for sustaining generalized trust. Fiscal policies that promote redistribution without undermining economic vitality, together with investments in high-quality education and elder care, are particularly relevant. As shown in the analysis, generalized trust improves in settings with higher life expectancy, older age structures, and strong educational systems.

Institutional regeneration is also crucial. Policies should aim to strengthen the legitimacy, responsiveness, and dynamism of public institutions. While institutional development increases generalized trust, stability without inclusiveness may weaken it. Improving citizens’ perceptions of democracy, fostering transparency, and promoting tolerance toward diverse beliefs strengthen social cohesion.

The educational system can also play a decisive role. Beyond improving access and quality, curricula should emphasize civic competence, empathy, constructive critical thinking, and intercultural coexistence. These capacities help mitigate the cautiousness observed among individuals with intermediate educational levels and support the development of bridging trust.

Religious dynamics present a manageable paradox. Personal religiosity correlates with lower generalized trust but higher neighborhood trust, while religious freedom and tolerance increase bridging connections. Public policies should therefore protect religious freedom, promote pluralism, and avoid any appearance of doctrinal imposition. Interreligious dialogue and inclusive public spaces contribute to strengthening community ties.

Urbanization and migration further condition trust patterns. Dense, anonymous urban environments reduce localized trust, while migration may initially weaken trust unless integration is actively promoted. Urban planning should therefore encourage community interaction through mixed housing, community centres, accessible public services, and shared spaces. Migration policies must facilitate gradual integration and avoid segregation, which is strongly associated with distrust.

In summary, public policy must distinguish between promoting generalized trust, through inclusive growth, equitable education, and institutional transparency, and preserving localized trust, which requires support for community networks, social care, and interpersonal interaction. Trust cannot be mandated; it arises where citizens perceive fairness, openness, and mutual recognition in both institutions and interpersonal relations.

### 6.2 Corporate strategy

As indicated in the literature review, trust is also a key factor in corporate dynamics. Among other advantages, trust influences productivity, organizational efficiency, and labour relations ( Ahmad and Hall, 2017; Bjørnskov, 2012; Bloom and van Reenen, 2010; Vanneste and Gulati, 2022), as well as consumer loyalty, commercial agreements, and market expansion ( Guiso, Sapienza, and Zingales, 2003; Knack and Keefer, 1997; Meyerson, Weick, and Kramer, 1996; North, 1990; Van Hoorn, 2017). Furthermore, trust reduces transaction and verification costs ( Arrow, 1972; Bjørnskov, 2015; Dearmon and Grier, 2009; Fukuyama, 1995; Putnam, 1993; Whiteley, 2000).

In this sense, businesses can implement diverse strategies ranging from improving the work environment and labour relations to promoting wage equity. All such initiatives should align with the personal and structural drivers of trust identified in this study linked to inclusion, economic and personal progress, and the embrace of diversity.

Many of these initiatives, including broader corporate social responsibility (CSR) policies addressing ethical obligations toward individuals and society, also generate measurable returns in business outcomes. Investments in internal educational programs, leadership development, and genuine corporate community-building complement public efforts to strengthen social trust. Likewise, companies should respect employees’ religious freedom, support their social responsibilities, and promote inclusive policies toward migrants. Economic security within the company—through comprehensive benefits, protection in adversity, stable employment, and internal career development, also plays a central role in fostering trust.

In emerging democracies, companies can further enhance institutional legitimacy by developing robust ethical governance systems and strengthening channels for community participation. These strategies align with the institutional variables and inclusivity conditions that our results identify as essential for trust formation.

In commercial relations, trust is reinforced when companies implement ethical and trans- parent supply chains and ensure fair payment terms. Transparency and procedural justice in contractual relationships extend trust beyond the firm toward business partners and consumers.

At the individual level, tolerance, experience gained with age, economic security, openness toward others, and adequate educational development are factors that help create comfortable and beneficial spaces of trust within corporate environments.

Ultimately, corporate settings replicate the same dynamics observed at the societal level: economic security and inclusion foster trust, whereas inequality and rigidity undermine it. Companies therefore share with public institutions the responsibility of creating transparent, fair, and plural environments in which trust can flourish.

A summary of the key results and their implications for policy and corporate strategy is presented in Table 6.

**Table 6.** Determinants of trust and policy/corporate implications.

Trust dimension	Key determinants	Public policy implications	Corporate implications
Generalized Trust (TMP)	+ Wealth, + EduHDI, + Age, − Gini, − Urbaniza- tion, − MYS, − RelImp, + RelTol, + LifeExp, + ISPn	Promote inclusive eco- nomic growth; invest in education quality; strengthen democratic institutions; reduce spatial inequality	Ensure wage equity; promote civic education and transparency; integrate trust-building practices in CSR initiatives
Trust in Strangers (TFT)	+ Wealth, + EduHDI, + ISPn, + RelTol, − Gini, − Urbanization, − RelImp, − MYS	Support integration policies; reduce inequality; promote bridging social capital	Foster multicultural interaction; train for inclusive communication; sup- port diversity and equal treatment
Trust in Neighbors (TN)	+ Wealth, + RelTol, + Re- lImp, ± Age (small), + ISPn, − Gini, − Urbaniza- tion, − MYS, − LifeExp	Reduce urban fragmentation; design inclusive public spaces; support community networks	Encourage local engagement; invest in employee community participation; support neighborhood level cohesion

### 6.3 Spillover of trust and directions for future research

The interaction between specific and generalized trust reveals important nuances. Trust among friends, family, and neighbors contributes significantly to generalized trust in environments featuring inclusive economic development, equality, high educational levels, and humanized urban settings. This suggests that policies aimed at improving specific trust can generate positive spillover effects on generalized trust. Likewise, tolerance and greater life expectancy act as sources of particularized trust that consistently translate into higher generalized trust. In addition, age exerts a small but consistently positive effect across all trust domains, suggesting that accumulated life experience provides a gradual, life–cycle based reinforcement of both localized and generalized trust. Net migration rates display only very small effects, but their generally positive direction is compatible with the idea that more diverse societies need not experience declines in trust, particularly when inclusive norms and integration policies are in place.

However, in the case of institutional stability, perceptions of democracy, the importance attributed to religion, and years of individual schooling, particularized trust does not easily transfer to generalized trust. This finding underscores the importance of legitimacy as a catalyst for facilitating such transfers. Similarly, our data reveal that the deficiencies detected in generalized trust for certain variables—including institutional stability, religious freedom, or perceptions of migration—are buffered and neutralized by finding acceptance at the particularized and group levels of trust. At the same time, high levels of urbanization systematically reduce the probability of reporting high neighbourhood trust, limiting the extent to which localized confidence can scale up to broader social spheres. Individual education also displays a nuanced pattern: intermediate educational attainment is often associated with greater critical distance and lower trust, whereas very high levels of education partially restore confidence, especially under favourable structural and institutional conditions.

Paradoxically, our research has found that in societies characterized by a strong appreciation for democratic values, greater religious freedom, higher educational attainment, and notable institutional stability, the results point to lower generalized trust. This pattern suggests that a deeper valuing of these societal pillars fosters a sharper awareness of the inherent complexity and imperfections within the system and social organization. This perspective can generate greater distrust because expectations become excessively idealized, leading to the development of critical awareness. Consequently, trust recalibrates toward closer, more specific social circles, rather than extending to the general social sphere.

To mitigate this effect of democratic, social, or religious fatigue, it is beneficial to support initiatives that help build bridges between critical awareness and civic engagement. Promoting transparency, creating inclusive platforms for deliberative participation, and fostering communication from religious and political leaders toward the citizenry are actions that can channel informed criticism toward building communities where trust is greater.

Finally, some ideas regarding possible methodological and theoretical improvements that could be implemented in future research can be highlighted. While the present work represents an extension of trust theory toward forms of particularized trust not sufficiently explored previously, the theory of trust remains underdeveloped. First, we emphasize the critical need for richer datasets. These must include a greater diversity of personal characteristics and socioeconomic factors to enhance the results. Although the World Values Survey (WVS) is an invaluable tool, which we have followed to maintain continuity with most previous studies that have used it, it lacks certain indicators that could significantly enrich trust research. Future work should, therefore, refine trust typologies and aim to obtain data that can help deepen understanding of these other forms of trust.

Furthermore, the findings across the various dimensions of trust reveal the necessity of working on very distinct contexts, including economic, structural, cultural, and individual ones. At a theoretical level, it would be of great interest to have interdisciplinary elements capable of evaluating the interaction of all these factors, and to have studies capable of combining perspectives from Economics, Sociology, and Political Science, given that in the construction of trust, as we have demonstrated, elements from all these fields are involved.

Our findings indicate that trust is strongest at the intersection of economic security, institutional legitimacy, and inclusive social norms. However, we believe that future research must expand on the variables identified here, addressing trust from a multidimensional perspective. This will enable the development of comprehensive scenarios, policies, and structural determinants essential for fostering greater trust in contemporary pluralistic societies.

## Ethical considerations

This study relies exclusively on anonymised secondary data. No experiments with humans were conducted, and according to Open Research Europe guidelines, no ethical approval is required for the use of publicly available data.

## Software availability

All statistical analyses were performed using Stata.

## Ethics and consent

Ethical approval and consent were not required for this study.

## Data Availability

All data underlying the results presented in this article are publicly available under open licences permitting reuse. Navarro-Ortiz, J., Moya Velasco, J., Soria Ruiz-Ogarrio, J., & Estévez-Mendoza, C. (2026). Database for “Determinants of Generalized Trust: Economic, Institutional, and Personal Factors” [Data set]. Zenodo. https://zenodo.org/records/18770379 This project contains the following underlying data:
-

WVS_Time_Series_1981-2022_stata_v5_0.dta Data are available under the terms of the Creative Commons Attribution 4.0 International. The appendix of this article is available in our repository. https://zenodo.org/records/18770379 All datasets are freely available and distributed under open licenses permitting reuse (CC-BY or equivalent, depending on the source). These datasets can be used to replicate the analyses and findings presented in this article.
