# Determinants of generalized trust: Economic, Institutional, and personal factors

**DOI:** 10.12688/openreseurope.22835.2

**Published:** 2026-09-01

**Authors:** Jorge Soria Ruiz-Ogarrio, Jorge Moya Velasco, Carlos Estevez Mendoza, Josep Navarro-Ortiz

**Affiliations:** 1 Facultad de Económicas, Universidad de Navarra, Pamplona, Navarre, Spain; 2 Facultad de Derecho, Empresa y Ciencias Políticas, Universidad Cardenal Herrera-CEU, Universidades CEU, Alfara del Patriarca, Valencian Community, Spain; 3 Facultad de Ciencias Económicas y Empresariales, Universidad Complutense de Madrid, Madrid, Spain and Facultad de Empresa, Economía y Derecho, CUNEF, Universidad, Madrid, Spain; 4 Analsisi Economic, Universitat de Valencia - Campus dels Tarongers, Valencia, Valencian Community, Spain

**Keywords:** trust, generalized trust, particularized trust, social capital, institutions, economic development, education, probit models.

## Abstract

**Background:**

Generalised trust matters for social cohesion, economic exchange and institutional life, but there is still disagreement about the conditions under which it emerges. This article studies whether the same economic, institutional and personal factors are linked to three forms of trust, namely trust in most people, trust in people met for the first time and trust in neighbours.

**Methods:**

The analysis uses individual data from the seventh wave of the World Values Survey, collected from 2018 to 2022, together with national economic and institutional indicators. The final models cover 49 countries. Trust in most people is estimated with a binary probit model, while trust in people met for the first time and trust in neighbours are estimated with ordered probit models. The main estimates use standard errors clustered by country and are compared with mixed-effects models, correlated random effects models and linear specifications.

**Results:**

Income inequality is the country-level factor most consistently linked to lower trust, and this association appears in the three trust domains. At the individual level, personal income, age, gender and education are also associated with trust. Education follows a curved pattern, with lower trust at intermediate levels and higher trust again among those with the highest education levels. National income is positively associated with trust in most people and in people met for the first time, but not clearly with trust in neighbours. Institutionalised democracy shows some evidence of a possible substitution between formal institutions and neighbourhood trust, although this result is not stable in all models. Religious importance is associated with lower trust in most people but higher trust in neighbours. Religious tolerance is positively related to trust, mainly through differences between countries rather than differences between individuals within the same country.

**Conclusions:**

Trust should not be treated as a single attitude with the same determinants in every context. Some factors, especially inequality and personal income, are linked to trust across several domains, while others depend on the social distance between the people involved. Policies aimed at strengthening trust therefore need to be clear about which type of trust they seek to build.

## 1. Introduction

Interpersonal trust is a cornerstone of social capital and a key resource for economic and institutional life. A large literature links trust to long-run growth ( Bjørnskov, 2012; Knack and Keefer, 1997; Zak and Knack, 2001), to the formation of human capital through cooperative behaviour ( Coleman, 1988; Papagapitos and Riley, 2009), to lower transaction costs ( Arrow, 1972), and to institutional quality and legitimacy ( Fukuyama, 1995; Knack, 2002; Putnam, 1993; Rothstein and Stolle, 2003). This paper inverts the question. Rather than asking what trust does for economies, it asks where trust comes from: the structural, institutional, and individual conditions under which people extend confidence to others ( Berggren and Jordahl, 2006; Bjørnskov, 2007; Delhey and Newton, 2005). Trust is therefore the dependent variable throughout; its consequences for growth enter only as motivation.

The paper’s point of departure is that’trust in others’ is not a single object. Trust in an abstract, anonymous humanity is conceptually and behaviourally distinct from trust extended to a concrete stranger at first contact, and from trust in neighbours — proximate, non-kin others about whom limited reputational information exists ( Delhey and Newton, 2005; Newton, 1997; Uslaner, 2002). Yet the empirical literature on trust formation overwhelmingly models a single outcome, usually the binary’trust in others’ item, and rarely examines whether the same determinants operate across different relational targets ( Freitag and Traunmüller, 2009; Glatz and Schwerdtfeger, 2022). This study analyses three trust domains jointly, namely trust in most people (TMP), trust in people met for the first time (TFT), and trust in neighbours (TN), which together span the spectrum from generalised to proximate trust. It examines which determinants operate uniformly across this range and which do so in a more selective way. This joint, domain-comparative design constitutes the central empirical innovation of the paper.

The analysis draws on the seventh wave of the World Values Survey (2018–2022) ( Haerpfer et al., 2022), combining individual-level responses with country-level structural indicators for roughly 67,000 respondents in 49 countries. A binary probit (TMP) and ordered probit models (TFT, TN) are estimated with standard errors clustered at the country level, and average marginal effects are reported for each level of each trust outcome. Because individuals are nested within countries, inference that ignores this structure overstates precision for country-level regressors ( Beugelsdijk, Groot, and van Schaik, 2004; Mikucka, Sarracino and Dubrow, 2017); the clustering correction, together with mixed-effects, correlated random effects, and linear robustness specifications, allows the analysis to distinguish associations that are genuinely robust from those that are model-sensitive.

The study contributes in three main ways. To begin with, it offers a joint analysis of generalised, first-encounter, and neighbourhood trust within a single empirical framework, showing that determinants differ systematically depending on the relational target, with some variables anchoring trust across the entire radius while institutions and religiosity instead redistribute it between proximate and distant others. At the same time, this comparison is grounded in an explicit theoretical framework, as it builds on six *ex-ante* hypotheses organised around three mechanism domains, covering economic security and distributional fairness, formal institutions and substitution, and community structure alongside cultural openness ( Section 3), rather than emerging from an unstructured collection of available covariates. Finally, the study applies a disciplined inference strategy in which each central result is evaluated in light of the multilevel structure of the data, so that the conclusions rely only on associations that remain robust after clustering, correlated random effects, and linear re-estimation.

The results yield a clear hierarchy. Income inequality is the most robust structural correlate of trust, negatively associated with all three domains in every specification, while personal income, a U-shaped education gradient, gender, and age operate as individual-level anchors across the full radius of trust. Institutionalised democracy is negatively associated with neighbourhood trust while leaving abstract generalised trust unaffected, consistent with a domain-specific institutional substitution mechanism, and personal religiosity strengthens neighbourhood solidarity while restricting generalised trust. Religious tolerance correlates positively with trust, although much of this association operates at the between-country level. Urbanisation, country-level schooling, and democratic perceptions do not survive the inference corrections and are reported transparently as model-sensitive.

Two clarifications delimit the scope. Although TN occupies an intermediate position on the radius of trust, it should not be read as particularised trust: particularised trust involves kin and intimate ties sustained by strong affective bonds, whereas neighbours are socially heterogeneous non-kin others, so TN belongs to the domain of bridging rather than bonding relations ( Delhey and Newton, 2005; Putnam, 2001). The determinants of strictly particularised trust (family and close personal relationships) fall outside the scope of this article and are left for future research.

The remainder of the paper is structured as follows. Section 2 reviews the literature on trust and its determinants. Section 3 develops the theoretical framework and derives six testable hypotheses. Section 4 describes the data, variables, and econometric specification. Section 5 reports the main results, organised by robustness tier. Section 6 examines average marginal effects across trust levels. Section 7 presents the theoretical contributions, policy implications, and directions for future research.

## 2. Literature review

Trust is defined as the willingness to accept vulnerability grounded in the expectation that others will refrain from opportunistic behaviour ( Chiles and McMackin, 1996; MacCrimmon and Wehrung, 1986; Rousseau et al., 1998). The literature distinguishes three interrelated forms: *generalised trust*, directed at strangers and abstract others ( Bjørnskov, 2007; Fukuyama, 1995); *particularised trust*, embedded in close and homogeneous networks of kin and acquaintances ( Newton, 1997; Uslaner, 2002); and *institutional trust*, reflecting confidence in formal political and legal structures ( Luhmann, 2000). These distinctions are organised around the concept of a *radius of trust* ( Delhey and Newton, 2005): generalised trust represents the widest radius, extending to socially heterogeneous others, while particularised trust is confined to the inner circle of known and related individuals.

The bulk of empirical research on trust has examined it as a determinant of economic performance. Higher trust reduces transaction and monitoring costs ( Arrow, 1972; Bjørnskov, 2015; North, 1990), supports human capital accumulation through cooperative norms ( Coleman, 1988), and reinforces institutional legitimacy ( Putnam, 1993; Rothstein and Stolle 2003). These mechanisms have been linked to productivity gains ( Ahmad and Hall, 2017; Bjørnskov, 2012), firm-level outcomes ( Vanneste and Gulati, 2022), and long-run economic development ( Knack and Keefer, 1997; Zak and Knack, 2001). However, causality is contested ( Delhey and Newton, 2005; Helliwell, 1996; Mikucka, Sarracino, and Dubrow, 2017), and measurement challenges — including response-bias across cultures and unobserved heterogeneity across countries — complicate cross-national inference ( Beugelsdijk, Groot and van Schaik, 2004; Bjørnskov, 2018; Kanitsar, 2022).

This study follows the complementary research tradition that inverts the causal arrow, treating trust as the *outcome* to be explained by structural, institutional, and individual conditions ( Berggren and Jordahl, 2006; Delhey and Newton, 2005; Glatz and Schwerdtfeger, 2022). Two broad explanatory approaches have emerged in this literature.

The first emphasises macro-structural and institutional conditions. Economic prosperity is repeatedly found to facilitate trust by reducing the stakes of misplaced confidence and lengthening the shadow of the future ( Berggren and Jordahl, 2006; Delhey and Newton, 2005). Income inequality, by contrast, is one of the most robust negative correlates of generalised trust: where disparities are wide, individuals perceive society as divided into winners and losers with conflicting interests, eroding the common ground on which cooperative expectations rest ( Bjørnskov 2007; Mikucka, Sarracino and Dubrow, 2017; Uslaner, 2002). Institutional quality also matters: impartial and effective institutions reduce uncertainty and signal that cooperative behaviour will be protected by formal rules, thereby substituting for or complementing informal trust norms ( Knack, 2021; Rothstein and Stolle, 2003; Rothstein and Uslaner, 2005). Urbanisation and demographic structure shape the local social environment by altering the density and stability of interpersonal ties ( Newton, 2004; Putnam, 2001).

The second strand focuses on individual and cultural characteristics. Education has received particular attention: while higher attainment is broadly associated with greater civic engagement and openness to diverse social environments ( Alesina and Ferrara, 2002; Green and Nelson, 2019), some evidence suggests a non-monotonic relationship in which intermediate levels of education promote critical distance while the highest levels restore confidence in others ( Huang, Chen and Chan, 2023; Paxton, 1999). Personal income and economic security consistently predict higher trust, as individuals with greater resources face lower perceived risks in social interaction ( Brandt, Wetherell and Henry, 2015; Delhey and Newton, 2005). Age tends to increase trust through accumulated social experience ( Berggren and Jordahl, 2006). Religious attitudes display a more complex pattern: personal religiosity may reinforce in-group solidarity while limiting openness toward out-groups, whereas tolerance toward other faiths tends to broaden the trust horizon ( Uslaner, 2002; Welch, Sikkink and Loveland, 2007; Zmerli and Newton, 2008). Gender and residential context, in particular the distinction between rural and urban settings, also shape trust formation, as rural embeddedness tends to be associated with denser local networks and stronger reputational incentives for cooperation ( Dinesen and Sønderskov, 2015; Putnam, 2001).

Despite this rich body of evidence, several limitations persist. Most studies focus exclusively on generalised trust, leaving the determinants of intermediate trust forms — such as trust in neighbours or trust in people met for the first time — comparatively underexplored ( Freitag and Traunmüller, 2009; Glatz and Schwerdtfeger, 2022). Few studies examine multiple trust domains simultaneously or report level-specific marginal effects that reveal how structural and individual factors redistribute the probability of different trust levels. This study addresses both gaps by combining a probit model for binary generalised trust (TMP) with ordered probit models for trust in people met for the first time (TFT) and trust in neighbours (TN), reporting clustered standard errors to account for within-country dependence, and interpreting results through level-specific average marginal effects.

## 3. Theoretical Framework

The literature reviewed above identifies a wide range of correlates of generalised trust, but it does not provide a systematic account of why those factors should operate differently across the three trust domains examined here. This section develops such an account around three domains drawn from institutional economics and social capital theory: (i) economic security and distributional fairness; (ii) formal institutions and their relationship to the architecture of trust; and (iii) community structure, cultural openness, and the social production of trust. From these domains we derive six testable hypotheses.

Two conceptual distinctions are necessary. First, following Uslaner (2002) and Newton (1997), we treat TMP, TFT, and TN as distinct relational outcomes. TMP is an abstract moral disposition toward humanity in general and does not depend on any specific interaction context. TFT refers to the extension of trust to concrete strangers at the moment of first contact, when no reputational information is available. TN occupies an intermediate position, since neighbours are not kin but share a residential environment that generates repeated exposure together with limited but meaningful reputational information. Against this background, we expect some factors to operate uniformly across all three domains, while others will vary depending on the degree of social distance involved. A natural question concerns whether TMP and TFT are sufficiently distinct to be treated as separate core outcomes, given that both refer to trust beyond the circle of known others. We retain both for two reasons. Conceptually, TMP captures a standing moral disposition toward an abstract humanity, whereas TFT captures the situational extension of trust to a concrete stranger; these two orientations can diverge, as individuals may hold an optimistic general view of others while remaining cautious at first contact. Empirically, the results support this distinction, since several determinants display different patterns across the two outcomes. Religious importance is strongly negative for TMP but indeterminate for TFT, and the patterns associated with institutions and perceptions of democracy also differ, which would be unlikely if both items captured the same underlying construct. Retaining the binary TMP item also preserves comparability with the standard generalised-trust literature, which is almost exclusively based on it ( Bjørnskov, 2007; Nannestad, 2008; Uslaner, 2002), while TFT provides the ordinal, situational counterpart that this literature lacks.

Second, following North (1990), we treat trust as a rational response to the incentive environment: individuals extend trust to the degree that the structural, institutional, and normative environment makes defection costly and cooperation rewarding ( Ostrom, 2000; Williamson, 1993).

### 3.1 Domain I: Economic Security and Distributional Fairness

The *security mechanism* holds that prosperity, both at the national level and at the personal level, raises the expected return from cooperative interaction and lowers the cost of being betrayed. Richer individuals in richer countries face lower stakes when trust is misplaced and benefit from longer time horizons that reward cooperation, which in turn facilitates the extension of trust even to strangers ( Berggren and Jordahl, 2006; Delhey and Newton, 2005). This logic is expected to operate across all three trust domains.

The *fairness mechanism*, developed by Uslaner (2002), holds that inequality signals that distributional norms are unreliable. Where economic outcomes are highly unequal, individuals perceive their fate as uncoupled from that of others, and the common ground on which cooperative expectations are built is eroded. This mechanism applies not only to trust in strangers but also to more proximate trust forms: even in neighbourhood or first-encounter contexts, pervasive inequality signals that the social order is structured by competing rather than shared interests.

Hypothesis 1 **(Economic Prosperity)** *Higher national income per capita and higher individual income are positively associated with trust across all three domains: TMP, TFT, and TN.*

Hypothesis 2 **(Distributive Fairness)** *Higher income inequality is negatively associated with trust in all three domains. The erosion of shared-fate perceptions induced by inequality weakens interpersonal confidence at every level of social distance.*

### 3.2 Domain II: Formal Institutions and the Architecture of Trust

Rothstein and Uslaner (2005) and Knack (2021) argue that formal institutions and interpersonal trust are linked through a structurally differentiated relationship whose effects vary across relational domains. When formal institutions are weak, individuals who need to coordinate tend to rely more heavily on informal networks of known and proximate others. By contrast, when formal institutions are strong, part of this informal reliance is displaced, allowing trust to extend across a wider radius because the institutional environment provides guarantees that informal networks would otherwise have to supply ( La Porta et al., 1996; Putnam, 1993).

This substitution logic generates domain-specific predictions. For abstract generalised trust in most people (TMP), the expansion of formal institutional quality should be associated with *higher* trust, since the institutional environment signals that strangers are unlikely to defect with impunity ( Rothstein and Stolle, 2003). For trust in neighbours (TN), however, stronger formal institutions may *reduce* the functional pressure to rely on residential networks of proximate others, thereby weakening neighbourhood trust — a substitution from localised solidarity toward broader institutional guarantees. Trust in people met for the first time (TFT) occupies an intermediate position where the direction of institutional effects is ambiguous.

We note a qualification: the substitution mechanism depends on the *impartiality* of institutional implementation rather than formal democratic quality alone ( Rothstein and Stolle, 2003; Rothstein and Uslaner, 2005). In transitional contexts, formal institutions may exist without being experienced as impartial, attenuating the predicted positive effect on TMP.

Hypothesis 3 **(Institutional Substitution)** *Stronger democratic institutions are associated with higher abstract generalised trust (TMP) but lower neighbourhood trust (TN), reflecting a substitution from proximity-based social ties toward broader institutional guarantees.*

*The effect on trust in first encounters (TFT) is expected to be attenuated.*

### 3.3 Domain III: Community Structure, Cultural Openness, and Education

*Community structure* affects trust through the opportunities for repeated interaction and reputational monitoring it creates ( Coleman, 1988; Putnam, 2001). Urbanisation disrupts these mechanisms by increasing anonymity and reducing the stability of local ties ( Newton, 2004; Wirth, 1938). The predicted negative effect should be most pronounced for neighbourhood trust and trust in first encounters, where reputational mechanisms are most relevant, and somewhat weaker for abstract generalised trust.

Rural residence at the individual level operationalises community embeddedness: rural residents interact within denser, more stable local networks that sustain strong reputational incentives. This should be associated with higher neighbourhood trust in particular.

*Cultural openness*, proxied by tolerance toward other religions, signals a normative orientation characterised by openness and a willingness to extend cooperative expectations across social boundaries ( Uslaner, 2002; Welch, Sikkink and Loveland, 2007). Normative openness is therefore expected to promote trust across all three domains. By contrast, religious importance may reinforce in-group solidarity, which is particularly relevant to TN, while at the same time reducing trust in strangers, thereby producing opposite effects across domains.

*Education* interacts with these mechanisms in a theoretically non-linear way. At lower levels, education may promote critical distance from naive trust; at higher levels, education expands social networks, increases exposure to diversity, and fosters civic orientations that restore confidence in others ( Green and Nelson, 2019; Paxton, 1999). This generates a U-shaped prediction that should apply across all three trust domains.

Hypothesis 4 **(Community Density)** *Higher urbanisation is negatively associated with all three forms of trust. At the individual level, rural residence is positively associated with neighbourhood trust (TN) through the community embeddedness mechanism.*

Hypothesis 5 **(Normative Openness)** *Religious tolerance is positively associated with trust in all three domains. Religious importance is positively associated with neighbourhood trust (TN) through in-group solidarity, but negatively associated with abstract generalised trust (TMP) and trust in first encounters (TFT) by restricting the normative scope through which others are evaluated.*

Hypothesis 6 **(Nonlinear Education)** *The relationship between individual educational attainment and all three trust forms is non-linear: education reduces trust at lower levels but increases it at higher levels, producing a U-shaped pattern captured by the linear-plussquared-specification.*

Table 1 summarises the six hypotheses and their expected directional predictions across the three trust domains.

**Table 1.** Summary of theoretical hypotheses and *ex ante* predicted signs.

H	Hypothesis	Key variables	TMP TFT		TN
1	Economic Prosperity	Wpc, ISPn	+	+	+
2	Distributive Fairness	Gini	−	−	−
3	Institutional Substitution	IDn	+	ambig.	−
4	Community Density	Urb, rural	−	−	−/+
5	Normative Openness	RelTol, RelImp	+/−	+/−	+/+
6	Nonlinear Education	EduLevN, EduLevNSQ	U	U	U

Predicted signs are stated *ex ante* from the framework of Section 3, prior to estimation.

+: positive.

−: negative.

ambig.: theoretically ambiguous.

U: U-shaped.

For Normative Openness the pair denotes (RelTol/RelImp). H3 predicts + for TMP; whether this prediction is borne out is assessed in Section 5.

TMP = trust in most people; TFT = trust in first encounters; TN = trust in neighbours.

## 4. Methodology

### 4.1 Data

The empirical analysis draws on the seventh wave of the World Values Survey (WVS), which covers the period 2018–2022 ( Haerpfer et al., 2022). The WVS is a large-scale international research programme that examines social, political, economic, and cultural values across a wide range of countries. Individual-level survey responses are combined with country-level structural indicators appended to the WVS-7 data file. These include GDP per capita (PPP), the Gini index, and urban population share from the World Bank, together with mean years of schooling from UNESCO and the institutionalised democracy score (Polity V) from the Center for Systemic Peace ( Marshall and Gurr, 2020). These indicators are provided as country-level snapshots, with source years mostly between 2018 and 2019, while the Gini index corresponds to the latest value available between 2012 and 2019. They are therefore treated as time-invariant national characteristics over the WVS-7 fieldwork window.

The final estimation sample contains 66,665 observations for the TMP model, 66,735 for the TFT model, and 66,989 for the TN model, covering 49 country clusters. Table 2 reports the distribution of observations by country in the estimation sample.

**Table 2.** Sample distribution by country (estimation sample).

Country	TMP	TFT	TN	Country	TMP	TFT	TN
Argentina	809	829	838	Lebanon	1,164	1,163	1,161
Armenia	1,167	1,163	1,152	Malaysia	1,313	1,313	1,313
Australia	1,565	1,575	1,575	Mexico	1,671	1,672	1,671
Bangladesh	1,191	1,188	1,191	Mongolia	1,628	1,633	1,633
Bolivia	1,915	1,930	1,930	Morocco	1,200	1,200	1,200
Brazil	1,419	1,418	1,414	Myanmar	1,198	1,198	1,198
Canada	2,950	2,950	2,950	Netherlands	1,153	1,137	1,154
Chile	862	879	877	Nicaragua	1,199	1,199	1,199
China	2,868	2,882	2,886	Pakistan	1,814	1,806	1,820
Colombia	1,498	1,498	1,498	Peru	1,293	1,291	1,295
Cyprus	758	768	769	Philippines	1,190	1,193	1,193
Czech R.	979	983	985	Romania	949	933	953
Ecuador	1,130	1,149	1,151	Russia	1,360	1,372	1,371
Ethiopia	1,207	1,207	1,210	Serbia	904	902	902
Germany	1,355	1,358	1,379	S. Korea	1,245	1,245	1,245
Greece	1,063	1,063	1,068	Tajikistan	1,189	1,189	1,189
Guatemala	1,117	1,109	1,101	Thailand	1,276	1,329	1,337
India	1,512	1,510	1,529	Tunisia	1,133	1,164	1,164
Indonesia	3,146	3,145	3,145	Turkey	2,198	2,225	2,228
Iraq	1,143	1,144	1,164	Ukraine	849	834	857
Japan	638	545	627	UK	1,444	1,444	1,445
Kazakhstan	942	961	965	US	2,459	2,456	2,457
Kenya	1,186	1,184	1,192	Uruguay	898	888	889
Kyrgyzstan	1,121	1,117	1,125	Vietnam	1,200	1,200	1,200
				Zimbabwe	1,197	1,194	1,194

*Total TMP*: 66,665; *Total TFT*: 66,735; *Total TN*: 66,989. TMP = Trust in most people (binary probit); TFT = Trust in people met for the first time (ordered probit); TN = Trust in neighbours (ordered probit). Observation counts reflect listwise deletion on all regressors.

### 4.2 Variables

***Dependent variables***

Three dependent variables capture distinct positions along the generalised-to-proximate trust spectrum. *TMP* (Trust in Most People) is a binary indicator coded 1 if the respondent agrees that’trust in others’ and 0 otherwise. It measures an abstract, unconditional moral orientation toward humanity as a whole. *TFT* (Trust in people met For the first Time) uses a four-point ordered scale (1 = do not trust at all; 4 = trust completely) and captures trust at the moment of first encounter, where no prior reputational information is available. *TN* (Trust in Neighbours) uses the same four-point scale and operationalises bridging trust among socially proximate but non-kin others who share a residential environment. Missing values and’Don’t know’ responses are excluded.

***Independent variables***

*Country-level variables* capture the structural environment in which respondents live. *Wpc* is the natural logarithm of GDP per capita, reducing skewness and capturing proportional differences in national income ( Delhey and Newton, 2005). The *Gini* coefficient, sourced from the World Bank, measures income inequality. *IDn* is a min–max normalisation of the Polity V institutional democracy score, measuring the quality of formal democratic institutions. The substitution mechanism of Rothstein and Uslaner (2005) turns on the impartiality of institutional implementation rather than on formal democratic quality, therefore IDn should be read as a proxy and it may understate the mechanism in transitional contexts whenever formal democratic institutions coexist with partial implementation. *Urb* is the share of the national population living in urban areas. *MYS* is a min–max normalisation of national mean years of schooling, capturing the historical stock of educational attainment ( Alesina and Ferrara, 2002).

A number of additional country-level variables, including BTI institutional stability, the Education HDI, religious freedom, migration rate and life expectancy, were examined in preliminary specifications. These variables are not included in the preferred model, since they fall outside the three domains outlined in the Theoretical Framework. In addition, none of them survived the clustering correction, and their inclusion substantially reduced effective country-level variation without improving explanatory power. Their exclusion does not alter the sign or significance of the core structural variables.

*Individual-level variables* reflect personal orientations and socioeconomic circumstances. *EduLevN* is a min–max normalisation of an eight-point educational attainment scale; its squared term *EduLevNSQ* captures potential non-linearities. *DemPerN* and *DemPerNSQ* capture respondents’ perceptions of the quality of democracy, also normalised and squared. *RelImp* measures the importance of religion in life; *RelTol* captures tolerance toward other religions. The variable *gender* is a binary indicator (1 = male) included to control for well-documented gender differences in trust formation. The variable *rural* is a binary indicator (1 = rural) that operationalises the community embeddedness argument: rural residents interact within denser, more stable local networks characterised by stronger reputational incentives. *Age* is measured in years. *ISPn* is a min–max normalisation of the self-reported income scale. Table 3 presents the definitions, coding rules and summary statistics of all variables used in the analysis.

**Table 3.** Variable definitions and summary statistics.

Variable	Definition	Computation	N	Mean	SD	Min	Max
*Dependent variables*							
TMP	Trust: most people	Binary (0 = no; 1 = yes)	95,883	0.24	0.43	0	1
FT	Trust in people met first time	Ordered scale (1–4)	95,859	1.99	0.81	1	4
TN	Trust in neighbours	Ordered scale (1–4)	96,393	2.82	0.80	1	4
*Country-level variables*							
Wpc	Log GDP per capita	NL of GDP per capita	91,857	9.84	0.90	7.75	11.77
Gini	Income inequality	Gini coefficient	79,976	37.13	6.13	24.9	53.9
IDn	Institutionalised democracy	Min–max normalisation of Polity V democracy score	89,309	0.64	0.36	0	1
Urb	Urban population	Urban population/Total population	95,550	68.50	20.34	21.23	100
MYS	Mean years of schooling	Min–max normalisation of years of schooling	93,400	0.61	0.23	0	1
*Individual-level variables*							
EduLevN	Educational attainment	Min–max normalisation of 8-point education scale	96,149	0.45	0.25	0	1
EduLevNSQ	Educationalattainment squared	Squared term of EduLevN	96,149	0.26	0.25	0	1
DemPerN	Democratic perception	Min–max normalisation democratic perception score	95,420	0.82	0.24	0	1
DemPerNSQ	Democraticperception squared	Squared term of DemPerN	95,420	0.73	0.32	0	1
RelImp	Importance of religion	Min–max normalisation of importance-of-religion scale	96,294	0.67	0.37	0	1
RelTol	Religious tolerance	Min–max normalisation of inverse of *only my religion is acceptable*	89,901	0.51	0.36	0	1
gender	Biological sex	Binary (1 = male; 0 = female)	97,125	0.53	0.50	0	1
rural	Rural residence	Binary (1 = rural; 0 = urban)	97,183	0.32	0.47	0	1
Age	Respondent age	Age in years	95,636	49.36	16.67	18	109
ISPn	Personal income level	Min–max normalisation of self-reported income scale	94,259	0.43	0.23	0	1

Source: Own elaboration from WVS Wave 7 (Haerpfer et al. 2022) and sources listed above.

### 4.3 Model Specification

Because the dependent variables differ in their measurement scale, two distinct models are estimated. The choice of model follows directly from the nature of each outcome variable.

***Binary Probit Model (TMP)***

TMP is a binary indicator (0/1). Defining the latent propensity to express generalised trust as *y _i_* ^∗^ = X *_i_β* + *ε _i_*, with *ε _i_* ∼ *N*(0 *, *1), the observed variable satisfies *y _i_* = 1 if *y _i_* ^∗^ *>* 0 and *y _i_* = 0 otherwise. The probability of trusting is:

$$\mathrm{Pr}\;\left(y_{i}=1|X_{i}\right)=\Phi \;\left(X_{i}\beta \right)$$

where Φ(·) is the standard normal CDF. Average marginal effects from the probit model report the change in the probability of *y _i_* = 1 associated with a unit change in each predictor.

***Ordered Probit Model (TFT and TN)***

TFT and TN take four ordered values. The latent variable *y _i_* ^∗^ is defined as above, and the observed outcome is generated by threshold crossings:

$$\;y_{i}=\left\{ \begin{array}{l} 1\;\text{if}\;{y_{i}}^{*}\leq \tau_{1}\\ 2\;\text{if}\;\tau_{1}<{y_{i}}^{*}\leq \tau_{2}\\ 3\;\text{if}\;\tau_{2}<{y_{i}}^{*}\leq \tau_{3}\\ 4\;\text{if}\;{y_{i}}^{*}>\tau_{3} \end{array}\right.$$

where *τ*_1_ *< τ*_2_ *< τ*_3_ are threshold parameters estimated jointly with *β.* Because coefficients represent shifts in the latent variable rather than probability changes, we compute level-specific average marginal effects (AMEs) for each trust category, enabling direct comparison across models.

### 4.4 Inference Strategy and Robustness

Because the data nest individual respondents within countries, a pooled model that treats observations as independent will understate standard errors for country-level variables. All primary models are therefore estimated with standard errors clustered at the country level, yielding heteroskedasticity- and autocorrelation-consistent standard errors that allow for arbitrary within-cluster correlation ( Cameron and Miller, 2015). With 49 country clusters, this correction is well-founded under standard asymptotic theory.

We implement three robustness checks. First, we estimate a mixed-effects probit (meprobit) for TMP and mixed-effects ordered probit models (meoprobit) for TFT and TN, each with a country-level random intercept. These specifications explicitly model the country-level variance component and estimate the intraclass correlation coefficient (ICC) — the share of total outcome variance attributable to country-level differences. Numerical stability of the mixed-effects estimates is verified by confirming identical results at 7, 15, and 30 quadrature integration points.

Two further robustness exercises deal with concerns that neither clustering nor a random intercept can resolve on their own. The random-intercept specification assumes that the country-level effect is uncorrelated with the regressors, so if unobserved national characteristics such as historical legacies, welfare-state traditions or cultural norms are correlated with individual-level covariates, both the pooled and the random-effects estimates may be biased. To address this, we estimate correlated random effects (CRE) models following the Mundlak–Chamberlain approach ( Chamberlain, 1980; Mundlak, 1978; Wooldridge, 2010), augmenting the mixed-effects models with the country-specific means of all individual-level regressors. This device allows the country effect to depend on the within-country composition of the covariates while avoiding the incidental parameters problem that direct country dummies would create in a probit framework. To verify that the findings are not an artefact of the probit link or of treating the four-point trust scales as ordinal, we re-estimate all models using ordinary least squares (OLS), with a linear probability model for TMP and linear regressions that treat TFT and TN as continuous, all with country-clustered standard errors ( Angrist and Pischke, 2009). As a final check on whether the number of clusters is adequate, we compute wild cluster bootstrap *p*-values for the key country-level coefficients ( Roodman et al., 2019). The mixed-effects estimates are reported in Appendix B, while the correlated random effects and linear specifications are reported in Appendices C and D, all in the Extended Data repository associated with this article.

## 5. Results and discussion

Table 4 presents the estimated coefficients from the primary models: a binary probit for TMP and ordered probit models for TFT and TN, all with standard errors clustered at the country level. A preliminary observation is essential. The clustering correction substantially inflates the standard errors of country-level regressors relative to a naive pooled specification, because respondents from the same country share common structural conditions. Several variables that appeared highly significant under unclustered standard errors, including institutional stability, the educational development index, religious freedom, migration rates and life expectancy, do not survive this correction and are therefore excluded from the preferred specification, as discussed in Section 4. The discussion that follows focuses only on those associations that can be distinguished from zero with confidence after applying this correction.

**Table 4.** Estimated coefficients (clustered standard errors).

		(1)	(2)	(3)
Variable	Description	TMP	TFT	TN
*Country-level variables*				
Wpc	Log GDP per capita	0.516***	0.345***	0.206
		(0.132)	(0.116)	(0.127)
Gini	Income inequality	−0.029***	−0.024***	−0.034***
		(0.008)	(0.007)	(0.006)
IDn	Institutionalised democracy	−0.381	0.099	−0.235**
		(0.252)	(0.132)	(0.117)
Urb	Urban population share	−0.008*	−0.007*	−0.006
		(0.004)	(0.004)	(0.004)
MYS	Mean years of schooling	−0.487	−0.608*	−0.349
		(0.411)	(0.320)	(0.268)
*Individual-level variables*				
EduLevN	Educational attainment	−0.551*	−0.601***	−0.558***
		(0.304)	(0.159)	(0.150)
EduLevNSQ	Education squared	0.946***	0.803***	0.628***
		(0.292)	(0.148)	(0.136)
DemPerN	Democratic perception	−0.194	−0.010	−0.050
		(0.244)	(0.189)	(0.178)
DemPerNSQ	Dem. perception squared	0.227	−0.015	0.178
		(0.201)	(0.158)	(0.152)
RelImp	Importance of religion	−0.433***	−0.079	0.102**
		(0.135)	(0.053)	(0.045)
RelTol	Religious tolerance	0.274***	0.134**	0.089**
		(0.081)	(0.065)	(0.043)
gender	Biological sex (male=1)	−0.045**	−0.094***	−0.089***
		(0.021)	(0.017)	(0.018)
rural	Rural residence (rural=1)	0.008	0.017	0.174***
		(0.035)	(0.039)	(0.029)
Age	Respondent age	0.003***	0.004***	0.007***
		(0.001)	(0.001)	(0.001)
ISPn	Personal income	0.359***	0.313***	0.203***
		(0.098)	(0.060)	(0.064)
Observations		66,665	66,735	66,989
Clusters		49	49	49
Lpl		−31,696.9	−76,181.0	−75,452.7
Pseudo *R* ^2^		0.115	0.027	0.035

Robust (cluster) standard errors in parentheses. *** *p <* 0 *.*01, ** *p <* 0 *.*05, * *p <* 0 *.*10. TMP binary probit; TFT and TN ordered probit. Pseudo *R* ^2^ is McFadden’s index. Magnitudes assessed through AMEs in Section 6 and Appendix A.

The discussion below organises the predictors according to how their associations behave across the full set of specifications — the clustered models of Table 4, the mixed-effects and correlated random effects models of Appendices B and C, and the linear models of Appendix D, all in the Extended Data repository associated with this article. Three tiers emerge: *cross-domain anchors*, whose associations hold across all trust domains and all estimators; *domain-specific mechanisms*, robust within particular relational targets but absent or indeterminate in others; and *model-dependent associations*, whose significance varies with the treatment of the country-level error component and which we therefore do not interpret substantively. Table 5 summarises the classification.

**Table 5.** Classification of predictors by robustness across domains and specifications.

Tier	Variables	Pattern
1. Cross-domain anchors	Gini; ISPn; EduLevN/EduLevNSQ; gender; Age	Same sign and significance in all three domains and in all specifications (clustered, RE, CRE, linear)
2. Domain-specific mechanisms	Wpc; IDn; RelImp; rural	Robust within particular domains (and, for Wpc and IDn, strongest where identification is cross-country); sign-consistent throughout
3. Model-dependent associations	RelTol; Urb;MYS; Dem- PerN/DemPerNSQ	Significance depends on the specification or operates at the between-country level; not interpreted as individual-level mechanisms

### 5.1 Cross-domain anchors

Income inequality (Gini) is the most robust country-level finding in the analysis, and the only structural variable that qualifies as a cross-domain anchor. It is negative and highly significant for all three outcomes in the clustered models (−0.029 for TMP, −0.024 for TFT, −0.034 for TN; all *p <* 0.01), and this result survives every robustness exercise: the mixed-effects and correlated random effects specifications reported in Appendices B–C, the linear models reported in Appendix D, and wild cluster bootstrap inference ( *p* = 0.001), all in the Extended Data repository associated with this article. This confirms Hypothesis 2 in its strongest form: pervasive inequality is associated with weaker interpersonal confidence at every level of social distance, from the most abstract generalised trust to neighbourhood-level interactions ( Mikucka, Sarracino and Dubrow, 2017; Uslaner, 2002).

Personal income (ISPn) is its individual-level counterpart: positive and highly significant in all three models ( *p* ≤ 0.001) and in every specification, providing robust support for the individual side of Hypothesis 1. Economic security consistently accompanies a greater willingness to extend trust, regardless of the relational target.

The nonlinear education specification delivers a consistent U-shaped pattern across all three trust domains, fully confirming Hypothesis 6. In every model the linear term (EduLevN) is negative — significant at *p <* 0.001 for TFT and TN and marginally so for TMP ( *p* = 0.069) in the clustered specification, and significant at p < 0.01 everywhere once the country effect is modelled, as reported in Appendices B–C in the Extended Data repository associated with this article — while the squared term (EduLevNSQ) is positive and highly significant throughout (all *p* ≤ 0.001). Education is associated first with critical distance from naive trust and then with restored confidence at higher attainment levels, whether the trust target is abstract humanity, a stranger encountered for the first time, or a neighbour. Because the gradient is identified from within-country variation and survives the CRE correction, it can be read as a genuinely individual-level regularity rather than a compositional artefact. To confirm that the quadratic specification implies a genuine U-shape over the observed range of education rather than a convex-but-monotone segment, we apply the test of Lind and Mehlum (2010), which requires the slope to be negative at the lower bound and positive at the upper bound of the data ( Table 6). In all three domains the lower-bound slope is significantly negative (all *p <* 0.001) and the upper-bound slope significantly positive (all *p <* 0.001), and the turning points lie strictly inside the unit interval — at EduLevN = 0.29 (TMP), 0.37 (TFT), and 0.44 (TN), with 95% confidence intervals that exclude both bounds. The test rejects monotonicity in favour of a U-shape at *p <* 0.001 for every outcome, confirming that education first reduces and then restores trust across the full radius.

**Table 6.** U-shape diagnostics for education (Lind–Mehlum test).

Outcome	Slope at lb	Slope at ub	Turning point	Lind–Mehlum *t*	*p*
TMP	−0.551 ^∗∗∗^	1.340 ^∗∗∗^	0.292 [0.140 *, *0.443]	4.43	*<*0.001
TFT	−0.601 ^∗∗∗^	1.005 ^∗∗∗^	0.374 [0.283 *, *0.465]	6.04	*<*0.001
TN	−0.558 ^∗∗∗^	0.699 ^∗∗∗^	0.444 [0.345 *, *0.543]	4.70	*<*0.001

Coefficients from Table 4 (clustered SE, 49 country clusters). “Slope at lb” is the marginal effect of education at the lower bound (EduLevN = 0), equal to the EduLevN coefficient; “slope at ub” is *β* ^ˆ^_EduLevN_ + 2 *β* ^ˆ^_EduLevNSQ_ evaluated at EduLevN = 1. Turning point = − *β* ^ˆ^_EduLevN_ */*(2 *β* ^ˆ^_EduLevNSQ_) with 95% delta-method CI in brackets.

In all three domains the slope is significantly negative at the lower bound and significantly positive at the upper bound, and the turning point lies strictly interior to the observed [0 *, *1] support; the Lind–Mehlum test therefore rejects monotonicity in favour of a U-shape at *p <* 0.001. The reported *t* is the binding (upper-bound) slope test. *** *p <* 0.01.

Gender and age complete the set of anchors. Men report systematically lower trust than women in all three domains ( *p* = 0.036 for TMP; *p <* 0.001 for TFT and TN), and age is positively associated with trust everywhere ( *p* = 0.003 for TMP; *p <* 0.001 for TFT and TN), consistent with the accumulation of cooperative social experience over the life course. Both patterns are stable across every estimator.

### 5.2 Domain-specific mechanisms

National income (Wpc) is positive and highly significant for abstract generalised trust ( *p <* 0.001) and for trust in people met for the first time ( *p* = 0.003), which supports the structural side of Hypothesis 1. By contrast, it does not reach significance for trust in neighbours ( *p* = 0.103). This pattern is theoretically coherent, since neighbourhood trust rests on reputational mechanisms that tend to be less sensitive to aggregate wealth than trust in strangers. The result for TMP is further corroborated by the wild cluster bootstrap ( *p* = 0.003) and by the linear specification. Because Wpc is identified from cross-country variation, its coefficient attenuates mechanically under the CRE specification, where country means absorb most of the between-country variation. For this reason, the clustered estimate remains the preferred one, as discussed in Appendix C in the Extended Data repository associated with this article.

The institutional variable (IDn) reveals the asymmetric pattern predicted by Hypothesis 3, in a domain-specific form. For abstract generalised trust the coefficient is negative and not significant ( *p* = 0.130); for first encounters it is positive and not significant ( *p* = 0.452); for trust in neighbours it is negative and significant ( *p* = 0.045). The neighbourhood result is corroborated by the linear specification (−0.178, *p <* 0.05) and is significant at the 10% level under wild cluster bootstrap inference ( *p* = 0.090), while it attenuates in the random-effects specifications that absorb between-country variation. The association is thus consistent in sign across every specification and significant in the preferred clustered and linear models, but it loses significance once the country effect is modelled (CRE) and is only marginal under the wild cluster bootstrap; we therefore treat it as suggestive evidence for the substitution mechanism rather than a firmly established result. Substantively, the pattern supports a substitution from localised proximity-based trust toward broader institutional guarantees: it is precisely at the neighbourhood level that formal institutions most directly displace the functional reliance on solidarity networks ( Rothstein and Stolle, 2003; Rothstein and Uslaner, 2005). That the predicted generalised-trust expansion does not materialise is consistent with the impartiality qualification discussed in Section 3.

Religious importance (RelImp) shows the expected domain differentiation, with a core pattern that remains robust across all specifications. It is negative and significant for TMP ( *p* = 0.001) and positive and significant for TN ( *p* = 0.024) in the clustered models, and both results are not only retained but, if anything, become more pronounced under mixed effects, CRE and the linear specification. This aligns with the bonding-versus-bridging mechanism proposed in Hypothesis 5, where personal religiosity goes hand in hand with stronger solidarity in proximate communities and weaker trust in abstract others. In the case of TFT, however, the evidence remains inconclusive. The effect is non-significant in the clustered model (p = 0.139) and turns mildly positive under CRE, as reported in Appendix C in the Extended Data repository associated with this article, so no clear conclusion can be drawn for that domain.

Rural residence is the most sharply domain-specific predictor in the analysis: not significant for TMP or TFT, but positive and highly significant for TN ( *p <* 0.001) in every specification, confirming the community embeddedness prediction of Hypothesis 4. Residents of denser, more stable local networks report substantially higher trust in their neighbours, with no comparable association beyond the residential environment.

### 5.3 Model-dependent associations

The remaining variables do not support substantive interpretation, and we report them transparently as model-dependent.

Religious tolerance (RelTol) is positive and significant across all three clustered models, with *p* = 0.001 for TMP, *p* = 0.038 for TFT and *p* = 0.039 for TN, and it also remains significant in the linear specification, which is consistent with the normative openness mechanism proposed in Hypothesis 5. However, this effect weakens substantially under the CRE correction. It becomes only marginal for TMP, with p = 0.070, and non-significant for TFT and TN, as reported in Appendix C in the Extended Data repository associated with this article. This pattern indicates that a large share of the association operates at the between-country level. Tolerant societies tend to display higher levels of trust, but within countries, differences between more and less tolerant individuals add comparatively little explanatory power. Accordingly, we interpret normative openness as a feature of the broader societal climate rather than as a uniform driver at the individual level, a distinction that is developed further in the Theoretical Contributions.

Urbanisation (Urb) carries a negative coefficient in all three models but reaches at most marginal significance (TMP: *p* = 0.054; TFT: *p* = 0.052; TN: *p* = 0.193) and weakens further in the random-effects specifications. The direction is consistent with Hypothesis 4, but the evidence is too fragile to sustain the strong anonymity claims found in earlier literature. Mean years of schooling (MYS) is negative throughout but never robustly significant once within-country dependence is accounted for; the country-level educational stock exerts no reliable independent association with trust in these data.

Perceptions of democracy (DemPerN, DemPerNSQ) are the clearest illustration of model dependence: not significant in any clustered model, yet significant in the mixed-effects and CRE specifications reported in Appendices B–C in the Extended Data repository associated with this article, which absorb country-level confounding through the random intercept and thereby attribute more variation to individual perceptions. Because the result is an artefact of how the country-level error component is treated rather than a stable feature of the data, we do not interpret it substantively.

Taken together, the analysis yields a hierarchy. Inequality, personal income, the education gradient, gender, and age are anchors that operate across the entire radius of trust and across every estimator. National income, institutions, religiosity, and rural embeddedness operate selectively, each within a theoretically interpretable domain. Tolerance, urbanisation, country-level schooling, and democratic perceptions do not survive scrutiny as individual level or specification-stable mechanisms, and the credibility of the remaining findings rests precisely on this transparency.

## 6. Marginal effects

The coefficients in Table 4 indicate the direction of associations with the underlying latent propensity to trust, but do not convey the probability-level magnitude of these relationships. Average marginal effects (AMEs) address this limitation: for the binary probit (TMP) they report the change in the probability of expressing generalised trust associated with a unit change in each predictor; for the ordered probit models (TFT, TN) they decompose this change into level-specific probability shifts across each of the four trust categories.

**Figure 1.**
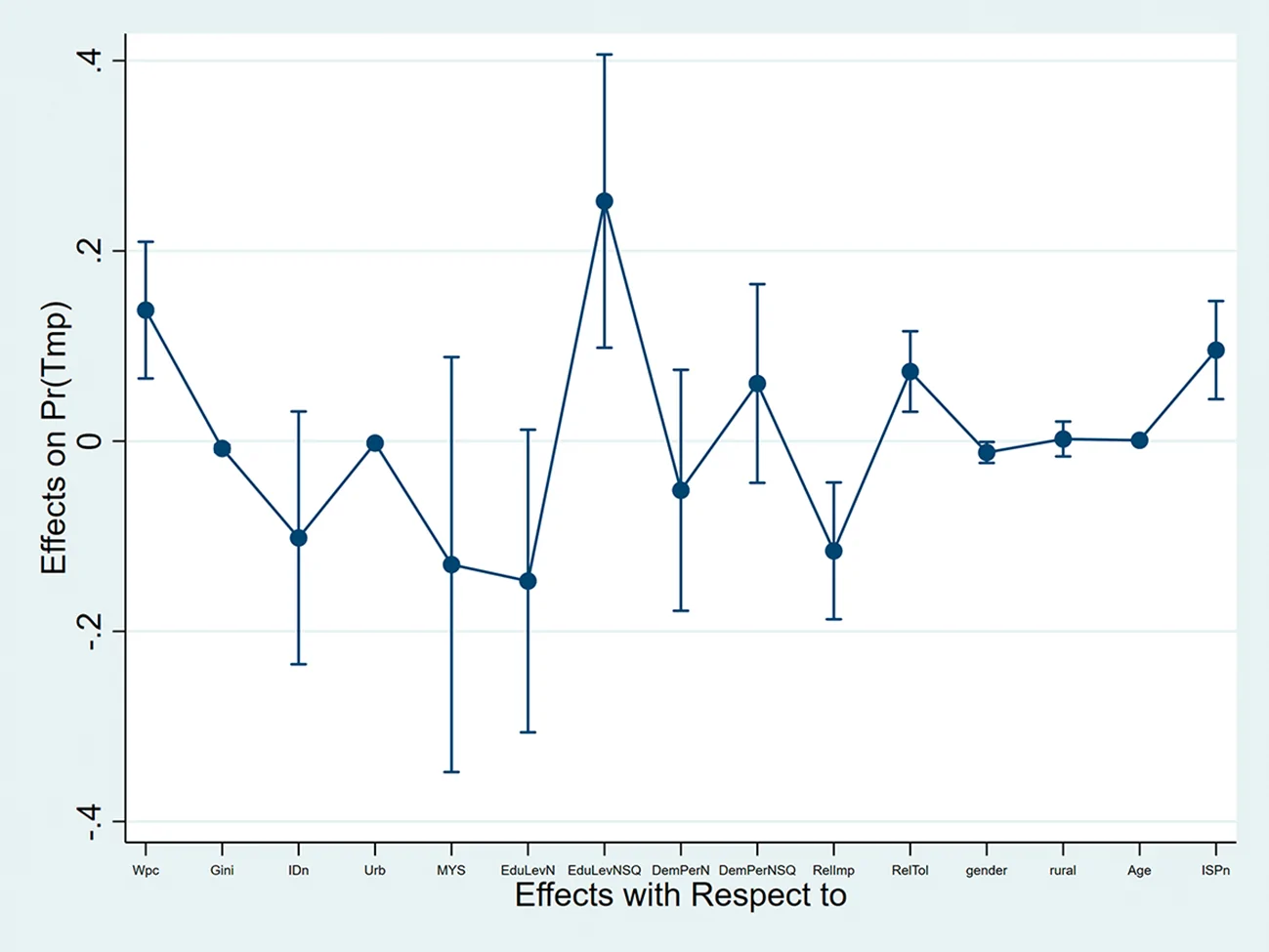
Average marginal effects on probability of expressing generalised trust (TMP = 1).

**Figure 2.**
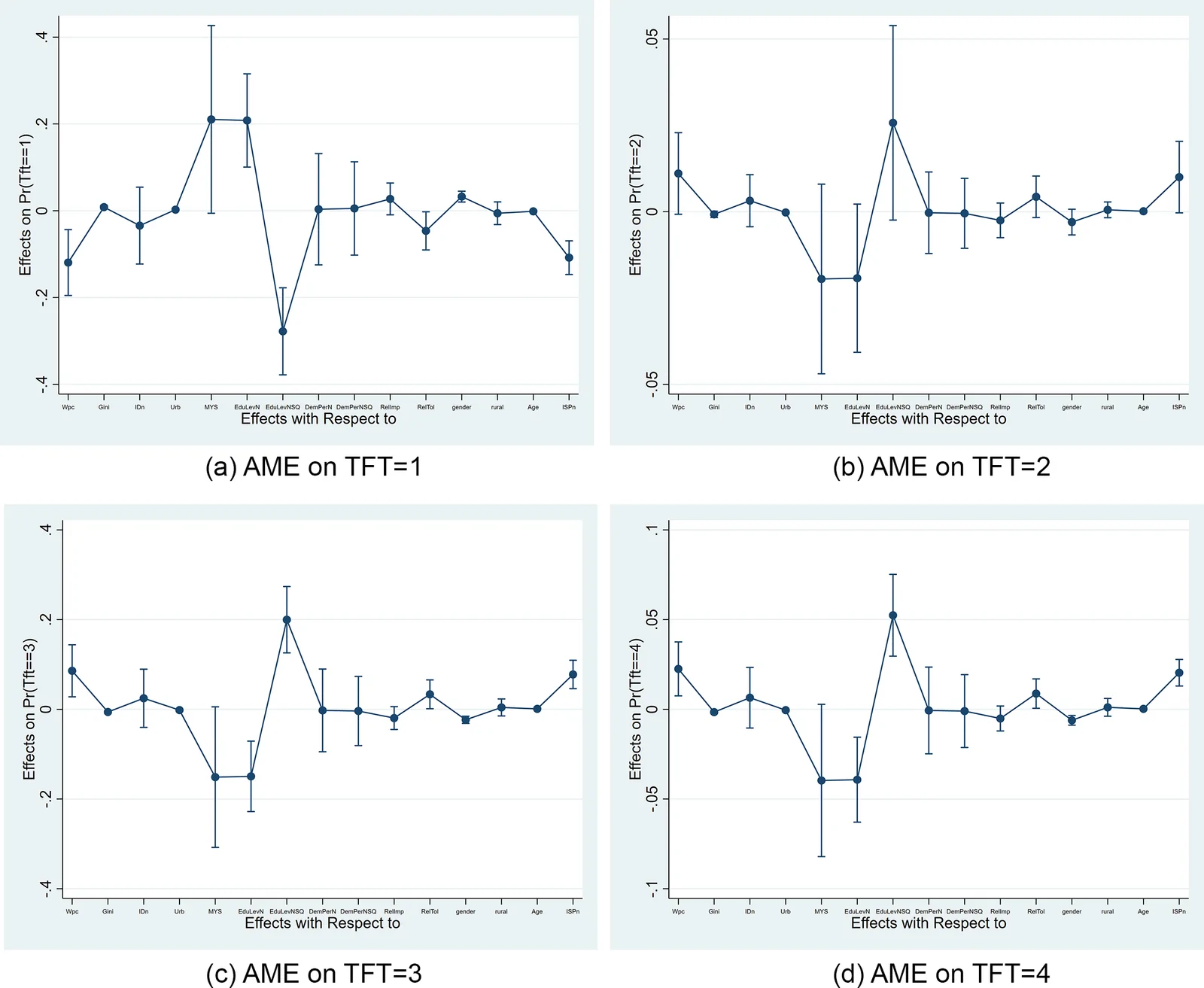
Average marginal effects on trust in people met for the first time (clustered 95% CIs).

**Figure 3.**
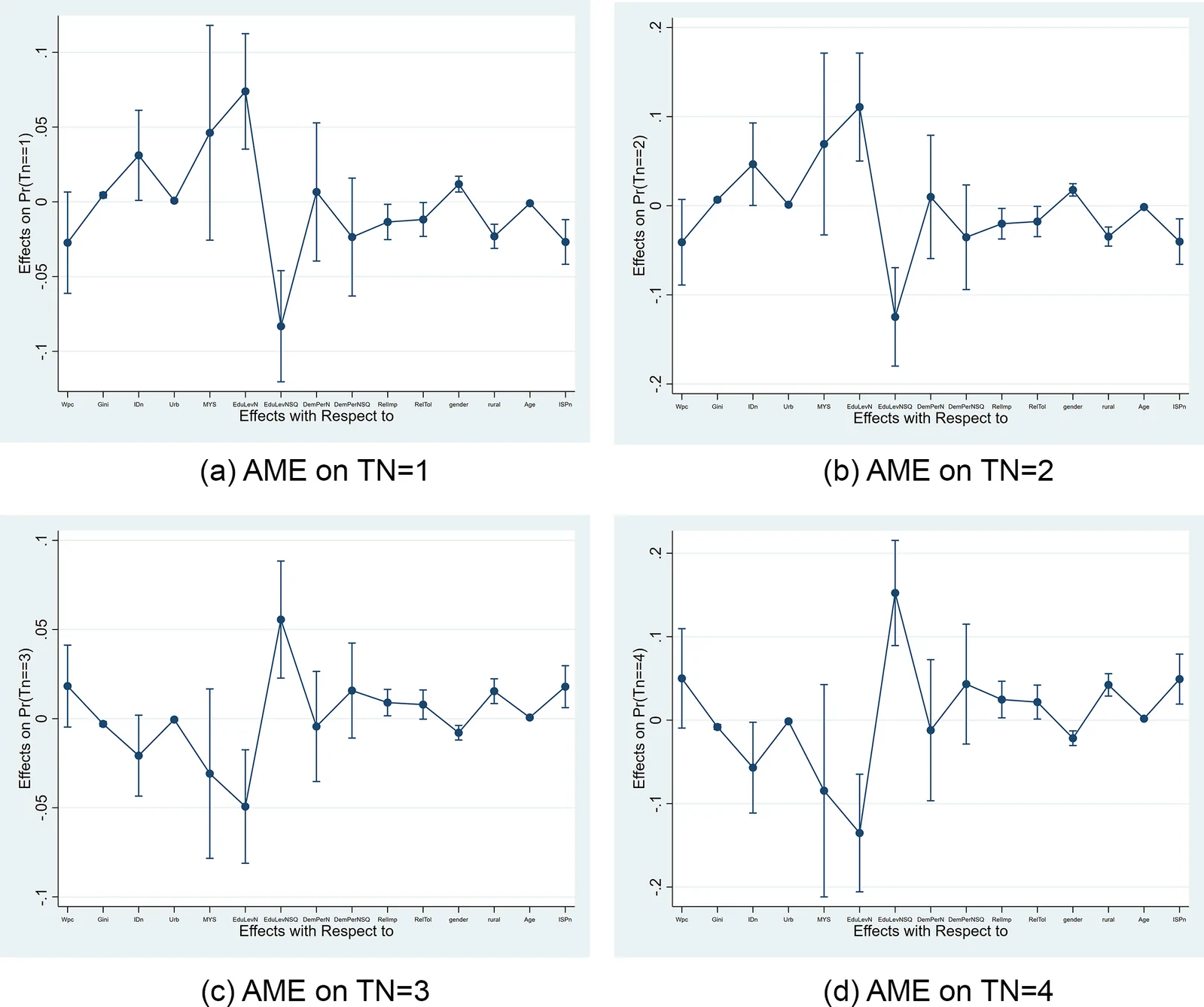
Average marginal effects on trust in neighbours (clustered 95% CIs).

Table 7 summarises the interpretation of AME sign patterns. Detailed numerical AME estimates for each model and level are reported in Appendix A in the Extended Data repository associated with this article; graphical summaries are presented in Figures 1, 2 and 3.

**Table 7.** General interpretation of AME patterns.

Sign pattern	Interpretation
Positive in low levels, negative in high levels	Factor reducing trust
Negative in low levels, positive in high levels	Factor increasing trust
Positive only in intermediate levels	Associated with moderate trust
Negative only in intermediate levels	Shifts towards extremes (high or low trust)
Low or near-zero across all levels	No substantive effect

### 6.1 Marginal Effects on Trust in Most People (TMP)

The AMEs for TMP, reported in full in Appendix A in the Extended Data repository associated with this article, translate the probit coefficients directly into probability-point changes in the likelihood of stating that most people can be trusted.

***Country-level factors***

Per capita income (Wpc) raises the probability of expressing generalised trust by approximately 13.8 percentage points ( *p <* 0.001), a substantively large effect that backs up the security mechanism: material prosperity lowers the stakes of misplaced trust and lengthens cooperative time horizons.

Income inequality (Gini) reduces the probability of generalised trust by 0.77 percentage points per unit increase in the Gini index ( *p <* 0.001). Scaled across the observed range of inequality in the sample (approximately 29 points from minimum to maximum), this amounts to a shift of roughly 22 percentage points, the largest structural predictor in probability terms.

Urbanisation (Urb) carries a small negative AME (−0.002, *p* = 0.058) and IDn a negative but non-significant AME (−0.102, *p* = 0.134), consistent with the coefficient-level results.

MYS and DemPerN show near-zero non-significant AMEs.

***Individual-level factors***

The U-shaped education pattern is clearly visible. EduLevN reduces the probability of generalised trust by 14.7 percentage points ( *p* = 0.070), while EduLevNSQ raises it by 25.2 percentage points ( *p* = 0.001). The net gradient confirms that highly educated individuals display substantially higher generalised trust than those at intermediate attainment levels.

Religious importance (RelImp) reduces the probability of generalised trust by 11.5 percentage points ( *p* = 0.002), the largest individual-level deterrent. Religious tolerance (RelTol) raises it by 7.3 percentage points ( *p* = 0.001). Personal income (ISPn) raises the probability by 9.6 percentage points ( *p <* 0.001). Gender reduces it by 1.2 percentage points ( *p* = 0.034). Age raises it by 0.09 percentage points per year ( *p* = 0.003), accumulating to meaningful differences across the life course.

### 6.2 Marginal Effects on Trust in People Met for the First Time (TFT)

***Country-level factors***

Per capita income (Wpc) shifts probability strongly from the lowest trust category (TFT = 1: −0.119, *p* = 0.002) toward the higher categories (TFT = 3: +0.086, *p* = 0.004; TFT = 4: +0.023, *p* = 0.003), confirming that economic prosperity is associated with first encounter trust across the full distribution.

Income inequality (Gini) produces the opposite redistribution: it increases the probability of TFT = 1 (+0.008, *p <* 0.001) and reduces the probability of TFT = 3 (−0.006, *p <* 0.001) and TFT = 4 (−0.002, *p* = 0.001). IDn and DemPerN show near-zero, non-significant AMEs across all four categories. Urbanisation reaches significance only at the lowest trust category (TFT = 1: +0.0025, *p* = 0.049) and is otherwise marginal, while mean years of schooling is marginal at the extreme categories (TFT = 1: *p* = 0.057); neither sustains a substantive interpretation, consistent with their classification as model-dependent.

***Individual-level factors***

The U-shaped education gradient is the dominant individual-level pattern. EduLevN increases the probability of TFT = 1 by 0.208 ( *p <* 0.001) and reduces TFT = 3 (−0.150, *p <* 0.001) and TFT = 4 (−0.039, *p* = 0.001), while EduLevNSQ reverses this entirely: it reduces TFT = 1 (−0.278, *p <* 0.001) and increases TFT = 3 (+0.200, *p <* 0.001) and TFT = 4 (+0.052, *p <* 0.001).

Religious tolerance (RelTol) shifts probability from TFT = 1 (−0.046, *p* = 0.038) toward TFT = 3 (+0.033, *p* = 0.041) and TFT = 4 (+0.009, *p* = 0.036). Religious importance (RelImp) shows near-zero and non-significant AMEs across all categories, consistent with its non-significant coefficient for TFT.

Gender increases the probability of TFT = 1 (+0.033, *p <* 0.001) and reduces TFT = 3 and TFT = 4 (both *p <* 0.001), indicating that men are substantially less likely to express high trust in first encounters. Personal income (ISPn) shifts probability strongly toward higher trust categories (TFT = 4: +0.020, *p <* 0.001). Age shows a consistent pattern of reducing TFT = 1 (−0.001, *p <* 0.001) and increasing TFT = 3 and TFT = 4 ( *p <* 0.001).

### 6.3 Marginal Effects on Trust in Neighbours (TN)

***Country-level factors***

Income inequality (Gini) is the most robustly signed country-level variable for TN. It increases the probability of TN = 1 (+0.004, *p <* 0.001) and TN = 2 (+0.007, *p <* 0.001) while reducing TN = 3 (−0.003, *p <* 0.001) and TN = 4 (−0.008, *p <* 0.001), confirming that inequality redistributes probability across the full neighbourhood trust distribution.

Institutionalised democracy (IDn) displays its most substantive AME pattern here. It increases the probability of TN = 1 (+0.031, *p* = 0.043) and TN = 2 (+0.047, *p* = 0.048) while reducing TN = 3 (−0.021, *p* = 0.073) and TN = 4 (−0.057, *p* = 0.040). This redistribution — shifting probability mass from the highest to the lowest neighbourhood trust categories — provides direct probability-level confirmation of the institutional substitution mechanism at the neighbourhood level. Stronger democratic institutions reduce the functional pressure to rely on proximity-based solidarity networks.

Wpc and Urb show near-zero or non-significant AMEs for TN, consistent with the coefficient-level results. MYS and DemPerN show non-significant AMEs throughout.

***Individual-level factors***

The U-shaped education gradient is again the dominant individual-level pattern, now with larger magnitudes. EduLevN increases the probability of TN = 1 (+0.074, *p <* 0.001) and TN = 2 (+0.111, *p <* 0.001) while reducing TN = 3 (−0.049, *p* = 0.002) and TN = 4 (−0.135, *p <* 0.001). EduLevNSQ reverses this: −0.083 for TN = 1 ( *p <* 0.001), −0.125 for TN = 2 ( *p <* 0.001), +0.056 for TN = 3 ( *p* = 0.001), and + 0.152 for TN = 4 ( *p <* 0.001).

Religious importance (RelImp) shows the bonding pattern: it reduces TN = 1 (−0.013, *p* = 0.025) and TN = 2 (−0.020, *p* = 0.021) while increasing TN = 3 (+0.009, *p* = 0.017) and TN = 4 (+0.025, *p* = 0.028), confirming that personal religiosity reinforces solidarity within proximate communities while restricting generalised trust. Religious tolerance (RelTol) also shifts probability toward higher neighbour trust (TN = 4: +0.022, *p* = 0.037), confirming that normative openness promotes trust even at the neighbourhood level.

Rural residence displays its strongest effect here: it reduces TN = 1 (−0.023, *p <* 0.001) and TN = 2 (−0.035, *p <* 0.001) while substantially increasing TN = 3 (+0.015, *p <* 0.001) and TN = 4 (+0.042, *p <* 0.001), confirming that community embeddedness in rural settings strongly promotes neighbourhood trust.

Gender reduces neighbour trust (TN = 4: −0.022, *p <* 0.001) and personal income increases it (TN = 4: +0.049, *p* = 0.001). Age shows a consistent positive redistribution toward higher neighbourhood trust across all four categories.

Overall, the AME analysis confirms that neighbour trust is shaped principally by income inequality, the institutional substitution mechanism, individual education, religiosity, rural embeddedness, gender, age, and personal income. The structural variables that dominate abstract generalised trust — particularly national income — are less influential for neighbourhood trust, where local network conditions and individual attitudes play a proportionally larger role.

## 7. Conclusions and policy and corporate implications

This study examined the factors associated with three trust domains — abstract generalised trust (TMP), trust in people met for the first time (TFT), and trust in neighbours (TN) — using data from the seventh wave of the World Values Survey. By combining binary probit and ordered probit models with country-clustered standard errors and average marginal effects, the analysis distinguishes robust structural and individual factors from associations that reflect statistical artefacts of overstated precision.

A primary contribution of the study is methodological: applying the clustering correction substantially narrows the set of significant predictors compared to naive pooled specifications, producing a more parsimonious and credible account of what actually is robustly associated with generalised and bridging trust. Several variables that appeared highly significant in earlier unclustered analyses, including institutional stability, the educational development index, religious freedom, life expectancy, migration rates and perceptions of democracy, do not survive the correction and therefore should not be treated as reliable causal predictors given the available data. The robustness of the remaining associations is further supported by correlated random effects models in the Mundlak–Chamberlain tradition, which allow the unobserved country effect to correlate with the covariates, as reported in Appendix C in the Extended Data repository associated with this article, as well as by linear probability and OLS specifications that relax the distributional assumptions of the probit framework, as reported in Appendix D in the same repository. Additional support comes from wild cluster bootstrap inference applied to the key structural coefficients.

Among the results that do survive all specifications, income inequality emerges as the single most robust country-level factor, negatively and significantly associated with trust across all three domains. This is consistent with the shared-fate mechanism of Uslaner (2002): pervasive inequality signals that distributional norms are unreliable, weakening interpersonal confidence regardless of social distance. The institutional substitution hypothesis receives domain-specific support: democratic institutions are negatively associated with neighbourhood trust (TN) — a result significant in the clustered and linear specifications and consistent in sign throughout — while having no significant effect on abstract generalised trust, precisely because it is at the neighbourhood level that formal institutions most directly displace the functional pressure to rely on proximity-based solidarity networks ( Rothstein and Stolle, 2003; Rothstein and Uslaner, 2005). National income is robustly positive for abstract and first-encounter trust but not for neighbourhood trust, where local conditions dominate.

At the individual level, the most consistent findings concern the U-shaped education gradient, which is confirmed across all three trust domains, along with personal income, religious tolerance, age and gender. Religious importance shows a clear variation across domains, reducing abstract generalised trust while reinforcing neighbourhood solidarity, in line with the bonding-versus-bridging distinction ( Putnam, 2001). In addition, rural residence is strongly associated with higher levels of neighbourhood trust, which fits well with the community embeddedness mechanism.

Several limitations should be acknowledged. The cross-sectional design prevents causal identification, so the observed associations are consistent with the theoretical mechanisms but may still reflect confounding. In addition, several of the regressors are plausibly endogenous to trust itself. Generalised trust, for instance, may shape support for redistribution, the consolidation of democratic institutions or tolerant attitudes, which means that causality may run in both directions. We considered instrumental-variables estimation (e.g., an IV probit); however, credible exclusion restrictions are not available for the broad set of structural and attitudinal covariates examined here, and estimates based on weak or invalid instruments would be less informative than the transparently associational ones we report. The correlated random effects specification reported in Appendix C in the Extended Data repository associated with this article addresses the component of endogeneity that arises from unobserved country-level heterogeneity correlated with the covariates; the remaining simultaneity between trust and its individual-level correlates can only be resolved with longitudinal or experimental designs, as discussed in Section 7.4. The 49-country sample is sufficient for cluster-robust inference under standard asymptotic theory ( Cameron and Miller, 2015) but sits at the lower end of what is recommended for reliable critical values; wild cluster bootstrap inference, reported in Appendix D in the Extended Data repository associated with this article, confirms that the key structural results are not sensitive to this. Survey-based trust measures may be subject to cultural interpretation biases across countries.

### 7.1 Theoretical Contributions

The study makes four contributions to the theory of trust formation.

To begin with, it offers a domain-differentiated account of the determinants of trust. Most theoretical work treats generalised trust as a single latent disposition (a stable moral orientation toward unknown others ( Hardin, 2002; Uslaner, 2002)), and most empirical work accordingly models a single outcome ( Freitag and Traunmüller, 2009; Nannestad, 2008). The framework developed in Section 3 instead treats TMP, TFT, and TN as distinct relational outcomes located at different points of the radius of trust ( Delhey and Newton, 2005), and derives ex-ante predictions about which mechanisms should operate uniformly across that radius and which should operate selectively. The results validate this differentiation: economic security mechanisms (national income, personal income, inequality) operate across domains, whereas institutional, religious, and residential mechanisms are domain-specific. Theories that model trust as a unidimensional disposition will therefore systematically mischaracterise the effects of institutions and culture, which redistribute trust across relational targets rather than raising or lowering it uniformly.

Second, the study refines the institutional substitution argument ( Rothstein and Stolle, 2003; Rothstein and Uslaner, 2005). The canonical formulation predicts a two-sided displacement in which strong formal institutions reduce reliance on proximate networks while, at the same time, enabling trust in distant others. Our evidence aligns only with the first of these mechanisms, and even then only tentatively. Democratic institutional quality shows a negative association with neighbourhood trust; the sign is consistent across all specifications and reaches significance in the clustered and linear models, although not under correlated random effects. By contrast, the expected expansion of abstract generalised trust does not appear. We therefore put forward this asymmetry as a hypothesis that still requires confirmation rather than as a settled result. In this light, the pattern suggests that substitution operates through the displacement of *functional* reliance on proximate ties, whereas the emergence of generalised trust depends on more than the formal presence of democratic institutions. A plausible explanation is the experienced impartiality of their implementation ( Rothstein and Stolle, 2003), something that standard democracy indices do not capture. One theoretical implication is that institutional development may erode localised social capital before its trust-generating counterpart fully takes hold. This points to a sequencing issue that the substitution literature has yet to articulate.

As a third point, the analysis identifies distributive fairness as the only domain-invariant *structural* mechanism. Income inequality is the sole country-level variable that remains negative and significant across all three trust domains and across every specification examined, including clustered, random effects, correlated random effects and linear models, with the robustness specifications reported in Appendices B–D in the Extended Data repository associated with this article. This finding extends Uslaner (2002). The shared-fate mechanism is typically theorised, and empirically tested, in relation to trust in abstract strangers, yet our results indicate that pervasive inequality also erodes interpersonal confidence within residential communities, where reputational information is available. In this sense, inequality appears to undermine not only optimism about anonymous others, but also the perception of a shared normative order on which even proximate cooperation depends.

Additionally, the study makes a fourth contribution by clarifying the level at which cultural and educational mechanisms operate, a distinction made possible by the correlated random effects design. The U-shaped education gradient and the bonding-versus-bridging effect of personal religiosity — negative for generalised trust, positive for neighbourhood trust ( Putnam, 2001; Welch, Sikkink and Lovelandv, 2007) — are robust on within-country variation, identifying them as genuinely individual-level mechanisms. Religious tolerance, by contrast, attenuates once country composition is controlled: much of its association with trust reflects differences between tolerant and intolerant societies rather than between tolerant and intolerant individuals within a society. This reframes normative openness as a property of the social climate closer to a contextual public good than to an individual trait and suggests that theories linking tolerance to trust ( Uslaner, 2002) should specify the societal level as the primary locus of the mechanism. More generally, the within/between decomposition illustrates how cross-national trust research can discipline theoretical claims about micro-level mechanisms that have, to date, rested largely on pooled associations.

### 7.2 Public Policy Priorities

The findings carry several policy implications that are tied to specific empirical results rather than general principles.

The most consistently supported policy lever is reducing income inequality. Gini is the only variable significantly associated with all three trust domains after the clustering correction. Fiscal redistribution and labour market policies that compress the income distribution are likely to have spillover effects on social cohesion beyond their direct economic objectives.

Personal income security at the individual level is equally robust. ISPn is positive and significant across all three models, with substantive marginal effects of 9–10 percentage points for TMP and similar magnitudes for TFT and TN. Policies that expand access to stable employment, social protection, and progressive transfer systems directly address this channel.

Neighbourhood planning and community design need to take the embeddedness mechanism into account. Rural residents tend to show substantially higher levels of neighbourhood trust, with AMEs of around 4 percentage points for the highest TN category. This suggests that urban planning could benefit from recreating some of the social conditions typical of rural settings. Encouraging denser and more stable social ties, for instance through mixed-use housing, accessible public spaces and well-integrated community services, may help offset the anonymity that often characterises large urban environments.

The institutional substitution result has a more nuanced implication. If the suggestive substitution pattern is borne out by future work, strengthening democratic institutions could reduce neighbourhood trust as a side effect of expanding formal guarantees. Policymakers in consolidating democracies should be attentive to this substitution process and invest simultaneously in community-level infrastructure to preserve the social fabric that formal institutions partially displace.

Educational policies need to move beyond simply expanding access if they are to address the U-shaped trust gradient. The strong negative AME of linear education, especially in the case of first-encounter and neighbourhood trust, suggests that intermediate levels of educational attainment tend to be linked to a more critical stance rather than straightforward, naive trust. This points to the value of curricula that place greater emphasis on civic competence, intercultural interaction and empathetic engagement. Such an approach could help mitigate this dip at lower attainment levels, while still preserving the trust-enhancing effects that appear at higher levels of education.

### 7.3 Corporate Strategy

Because trust lowers transaction and monitoring costs and supports cooperative norms within firms ( Arrow, 1972; Coleman, 1988; North, 1990), the individual-level anchors identified above have organisational counterparts, offered here as conjectures rather than tested claims. Personal income security (ISPn) is robustly associated with trust across all domains, suggesting that wage stability and comprehensive benefits may support the economic security underlying interpersonal confidence. The U-shaped education gradient implies that workforce-development efforts focused only on technical skills may leave employees in the low-trust trough at intermediate attainment, pointing to a complementary role for civic and interpersonal competencies. The consistently negative association for male respondents and the positive age gradient are likewise suggestive for inclusion and knowledge-transfer practices. We stress that these inferences extrapolate from cross-national survey associations to organisational settings the data do not observe and are therefore directional rather than prescriptive.

A summary of the key factors and their implications across trust domains is presented in Table 8.

**Table 8.** Robust factors of trust and implications (post-clustering).

Trust Domain	Key Factors	Public Policy Implications	Corporate Implications
Generalised Trust (TMP)	+Wpc, −Gini, −RelImp, +RelTol, +ISPn, −gender, +Age, U-shape education	Reduce inequality; protect income security; promote tolerant civic norms	Wage equity; diversity and inclusion programs; leadership transparency
Trust in Strangers (TFT)	+Wpc, −Gini, +RelTol, +ISPn, −gender, +Age, U-shape education	Reduce inequality; invest in higher education; promote religious pluralism	Multicultural workplace; income security; crosscultural competence training
Trust in Neighbours (TN)	−Gini, −IDn (substitution), +RelImp, +RelTol, +rural, −gender, +Age, +ISPn, U-shape education	Reduce inequality; invest in community infrastructure; neighbourhood design	Local engagement; employee community participation; inclusive workplace

### 7.4 Spillover Effects and Directions for Future Research

The domain-specific patterns documented here suggest that the determinants of trust do not translate uniformly across relational contexts. Income inequality is the closest to a universal corrosive factor. Personal income and religious tolerance operate consistently across all three domains. The institutional substitution effect operates selectively at the neighbourhood level. Education follows a U-shaped path that is domain-invariant in shape but varies in magnitude. These heterogeneous spillover patterns imply that policies designed to strengthen one form of trust may not automatically reinforce others: strengthening formal institutions may reduce neighbourhood trust even as it leaves abstract generalised trust unchanged.

Future research should address several limitations of the current analysis. Longitudinal data would allow direct examination of how changes in inequality, institutional quality, or individual income affect trust dynamics over time. More refined geographic identifiers would allow neighbourhood-level structural characteristics to be incorporated directly. Experimental designs could help isolate the causal mechanisms underlying the clustering-robust associations documented here. Finally, extending the analysis to additional trust domains — including institutional trust in governments and courts — would allow a fuller mapping of the structural conditions that shape the entire architecture of interpersonal and institutional confidence. A natural companion line of research concerns particularised trust — specifically trust in family members and people personally known — where the mechanisms governing close interpersonal relations differ substantially from those identified here. The relationship between particularised and generalised trust, and the conditions under which structural improvements in one domain generate spillovers into the other, represents a promising direction that the present study leaves open.

### Ethical considerations

This study relies exclusively on anonymised secondary data. No experiments with humans were conducted, and according to Open Research Europe guidelines, no ethical approval is required for the use of publicly available data.

## Ethics and consent

Ethical approval and consent were not required for this study.

## Data Availability

All data underlying the results presented in this article are publicly available under open licences permitting reuse. Navarro-Ortiz, J., Moya Velasco, J., Soria Ruiz-Ogarrio, J., & Estévez-Mendoza, C. (2026). Database for “Determinants of Generalized Trust: Economic, Institutional, and Personal Factors” [Data set]. Zenodo. https://zenodo.org/records/ 21063528 This project contains the following underlying data:
−

WVS_Time_Series_1981-2022_stata_v5_0.dta Data are available under the terms of the Creative Commons Attribution 4.0 International. The appendix of this article is available in our repository. https://zenodo.org/records/21063528 All datasets are freely available and distributed under open licenses permitting reuse (CC-BY or equivalent, depending on the source). These datasets can be used to replicate the analyses and findings presented in this article.

## Software availability

All statistical analyses were performed using Stata.
