# Supplementary material for: Determinants of generalized trust: Economic, Institutional, and personal factors
Source: Open Res Eur. 2026 Sep 1;6:123. Originally published 2026 Apr 17. [Version 2] doi: 10.12688/openreseurope.22835.2 (PMC13562996; doi:10.12688/openreseurope.22835.2)
Supplement: Peer review history

# Reviewer report: Reviewer response for version 2

**DOI:** 10.21956/openreseurope.26506.r80112

**Published:** 2026-10-07

**Authors:** Mahyudin Ahmad

**Affiliations:** 1 Faculty of Business and Management, Universiti Teknologi MARA - Kampus Perlis, Arau, Perlis, Malaysia

The revised manuscript clearly states its main contribution, with particular emphasis on the multi-domain comparison of trust and appropriately avoids overstating causal claims. The discussion frames the findings as associations rather than causal effects, especially concerning macro-level variables, and explicitly acknowledges the study’s limitations. The results, including the complex modeling outputs, are presented clearly with sufficient explanations, maintaining accessibility in the main text. The authors’ decision to move detailed tables to the appendices appears appropriate. Additionally, the "Theoretical Contributions" subsection effectively clarifies how this work advances understanding of trust across different domains and systematically integrates relevant literature.

 Overall, the authors have made significant and appropriate revisions in response to the detailed review. The manuscript now articulates its contributions clearly, transparently manages its limitations, and is well-presented.

**Recommendation: Accept and index as it is.**

If applicable, is the statistical analysis and its interpretation appropriate?

Yes

Is the study design appropriate and is the work technically sound?

Partly

Is the work clearly and accurately presented and does it engage with the current literature?

Yes

Are the conclusions drawn adequately supported by the results?

Yes

Are sufficient details of methods and analysis provided to allow replication by others?

Yes

Are all the source data and materials underlying the results available?

Yes

Reviewer Expertise:

Applied economics, panel data econometrics.

I confirm that I have read this submission and believe that I have an appropriate level of expertise to confirm that it is of an acceptable scientific standard.

# Reviewer report: Reviewer response for version 2

**DOI:** 10.21956/openreseurope.26506.r80113

**Published:** 2026-09-04

**Authors:** Andreas Kyriacou

**Affiliations:** 1 Università de Girona, Girona, Spain

The revised manuscript represents a substantial and constructive response to my report. The contribution is now appropriately centred on the comparison among trust domains; the theoretical framework is considerably more focused; the multilevel structure of the data is addressed through country-clustered inference, mixed-effects models, correlated random effects and linear specifications; and the causal and interpretive claims have generally been moderated. The earlier speculative interpretations have also been removed, and the treatment of marginal effects is much more concise. I therefore no longer have any major concerns about the manuscript.

If applicable, is the statistical analysis and its interpretation appropriate?

Partly

Is the study design appropriate and is the work technically sound?

Yes

Is the work clearly and accurately presented and does it engage with the current literature?

Yes

Are the conclusions drawn adequately supported by the results?

Partly

Are sufficient details of methods and analysis provided to allow replication by others?

Yes

Are all the source data and materials underlying the results available?

Yes

Reviewer Expertise:

Political Economy.

I confirm that I have read this submission and believe that I have an appropriate level of expertise to confirm that it is of an acceptable scientific standard.

# Reviewer report: Reviewer response for version 1

**DOI:** 10.21956/openreseurope.24692.r73788

**Published:** 2026-06-10

**Authors:** Mahyudin Ahmad

**Affiliations:** 1 Faculty of Business and Management, Universiti Teknologi MARA - Kampus Perlis, Arau, Perlis, Malaysia

This study reverses the traditional focus of social capital literature - which typically views interpersonal trust as an engine for economic growth - to explore the structural, institutional, and personal determinants that allow trust to emerge in the first place.

 The strength of the paper lies in its massive trust data collected from the WVS survey of 97,200 respondents, covering three specific domains of trust. This allows for a more nuanced analysis of trust determinants at both the personal and general levels, as well as an empirical framework based on binary/ordered probit models for the dichotomous levels of trust.

 However, several issues may undermine the quality of the findings, for example:

1. **Endogeneity of trust to its determinants** – Although the article combines national-level with individual-level indicators of trust, it does not completely mitigate the endogeneity issue. The estimation method appears to do nothing to resolve this problem empirically. I suggest the authors explore the IV probit estimator instead of using the standard probit estimation (the Stata command is ivprobit).
2. **Unobserved heterogeneity due to diverse sample countries** – How do the authors confirm that this bias is fully isolated and eliminated from the estimation? Although the authors acknowledge this issue when interpreting the large negative coefficients for MYS, what about the other determinants' coefficients? I am not confident that the simple binary/ordered probit can overcome this bias. Please explore possible solutions to this bias, such as:
  (i) correlated random effects (the Mundlak-Chamberlain approach, which involves including country-specific fixed effects as a function of country-specific time averages of the IVs, thereby avoiding the incidental parameters problem that would arise if a fixed parameter for country-specific effects were included in a probit regression);
  (ii) fixed effects logit; or
  (iii) a linear probability model with fixed effects.

That will be all.

If applicable, is the statistical analysis and its interpretation appropriate?

Yes

Is the study design appropriate and is the work technically sound?

Partly

Is the work clearly and accurately presented and does it engage with the current literature?

Yes

Are the conclusions drawn adequately supported by the results?

Yes

Are sufficient details of methods and analysis provided to allow replication by others?

Yes

Are all the source data and materials underlying the results available?

Yes

Reviewer Expertise:

Applied economics, panel data econometrics.

I confirm that I have read this submission and believe that I have an appropriate level of expertise to confirm that it is of an acceptable scientific standard, however I have significant reservations, as outlined above.

# Response

**Published:** 2026-08-27

**Authors:** Josep Navarro-Ortiz

**Affiliations:** Anàlisi Econòmic, Universitat de Valencia, Valencia, Valencian Community, Spain

We thank the reviewer for the precise and actionable report. Both points have been addressed: the second through full implementation of the reviewer's preferred solution, the first through a combination of new analysis, reframing, and an explicit treatment in the manuscript.

**1. Endogeneity of trust to its determinants; explore the IV probit estimator (ivprobit).** We took this suggestion seriously and considered IV estimation carefully. We concluded — and now state explicitly in the limitations discussion (Section 7) — that a credible IV strategy is not available in this setting, for a reason specific to the paper's design: the analysis examines a broad set of structural and attitudinal covariates (national income, inequality, institutions, urbanisation, schooling, religiosity, tolerance, democratic perceptions, personal income), and valid exclusion restrictions would be required for each endogenous regressor of interest. Instruments proposed in the trust literature for single variables do not extend to this multivariate setting, and ivprobit estimates based on weak or invalid instruments would, in our judgement, be less informative than transparently associational estimates. We have therefore taken three complementary measures. First, the manuscript's causal language has been systematically moderated: all empirical claims are now framed as associations, and the limitations paragraph names the endogeneity problem explicitly, including the possibility of reverse causality from trust to institutions, redistribution, and tolerance. Second, the component of endogeneity arising from unobserved heterogeneity — which overlaps with the reviewer's second point — is addressed directly by the new correlated random effects analysis described below. Third, the future-research section identifies longitudinal and experimental designs as the appropriate route to causal identification. We hope the reviewer agrees that this is the intellectually honest treatment given the data.

**2. Unobserved heterogeneity across countries: correlated random effects (Mundlak–Chamberlain), fixed-effects logit, or a linear probability model with fixed effects.** We have implemented the reviewer's option (i) — the Mundlak–Chamberlain correlated random effects approach — exactly as described in the report, and we are grateful for the precise recipe. Appendix C augments the mixed-effects probit (TMP) and ordered probit (TFT, TN) models with country-specific means of all individual-level regressors, allowing the country effect to depend on within-country covariate composition while avoiding the incidental parameters problem that direct country dummies would create. The results are informative in both directions. The joint Wald tests on the country means reject the random-effects orthogonality assumption for TMP (χ²(10) = 58.36, p < 0.001) and, marginally, for TFT (χ²(10) = 18.56, p = 0.046), but not for TN (χ²(10) = 14.21, p = 0.164) — confirming that the bias the reviewer suspected was real for two of the three outcomes and that the correction was warranted. Substantively, all individual-level conclusions survive: the U-shaped education gradient, personal income, gender, age, rural residence, and the domain-differentiated religiosity pattern retain their sign and significance on within-country variation. Income inequality remains negative and significant in all three CRE models, while other country-level coefficients attenuate as the country means absorb between-country variation; the manuscript discusses this transparently and the Results section now classifies every predictor by its robustness across specifications (Table 5), so that no coefficient — not only MYS — is interpreted beyond what the full set of estimates supports. Regarding options (ii) and (iii): fixed-effects logit and an LPM with country fixed effects would absorb all country-level regressors (national income, inequality, institutions, urbanisation, schooling), eliminating the structural half of the analysis, which is why we regard the CRE approach as the appropriate variant of the reviewer's suggestion for this design. We have nonetheless implemented the spirit of option (iii): Appendix D reports a linear probability model for TMP and OLS for TFT and TN with country-clustered standard errors, whose estimates are closely concordant with the probit results, together with wild cluster bootstrap p-values (Roodman et al., 2019) confirming that the key structural coefficients are robust to the 49-cluster count. We thank the reviewer again for two suggestions that have materially strengthened the credibility of the paper's findings.

# Reviewer report: Reviewer response for version 1

**DOI:** 10.21956/openreseurope.24692.r73790

**Published:** 2026-05-26

**Authors:** Albert Jiansong Zheng

**Affiliations:** 1 The Hong Kong Polytechnic University, Hung Hom, Hong Kong

This study investigates the factors associated with trust, including binary-format generalized trust, trust in people met for the first time, and trust in neighbors using WVS data. The topic is relevant and meaningful. However, there are several academic concerns that should be addressed.

 Please provide the full names of all abbreviations under the tables, particularly in Table 4.

 The analyses may mix different levels of variables. For example, per capita GDP and the Gini index are country-level variables. Although these variables can be directly included as independent variables, the estimations may be biased if the nested structure of the data is ignored. Multilevel models are recommended to better identify these cross-level patterns.

 Given that trust variables can be treated as continuous measures, OLS regression models may also be used to examine the robustness of the findings.

 As pooled cross-sectional data are used in this study, causal language should be applied more cautiously throughout the manuscript.

 Trust in people met for the first time appears to conceptually overlap with binary-format generalized trust. It may be more appropriate to treat binary-format generalized trust as a robustness check rather than as a separate core outcome variable.

 More literature should be cited to strengthen the discussion section. For example, the findings should be compared more systematically with previous empirical studies.

 Please add a “Theoretical Contributions” subsection in the discussion section to clarify the theoretical significance and originality of this study.

If applicable, is the statistical analysis and its interpretation appropriate?

Partly

Is the study design appropriate and is the work technically sound?

Partly

Is the work clearly and accurately presented and does it engage with the current literature?

Yes

Are the conclusions drawn adequately supported by the results?

Yes

Are sufficient details of methods and analysis provided to allow replication by others?

Yes

Are all the source data and materials underlying the results available?

Yes

Reviewer Expertise:

Cross-cultural psychology, Health psychology

I confirm that I have read this submission and believe that I have an appropriate level of expertise to confirm that it is of an acceptable scientific standard, however I have significant reservations, as outlined above.

# Response

**Published:** 2026-08-27

**Authors:** Josep Navarro-Ortiz

**Affiliations:** Anàlisi Econòmic, Universitat de Valencia, Valencia, Valencian Community, Spain

We thank the reviewer for the constructive report. All seven points have been addressed; six through changes to the manuscript and one through a reasoned clarification that we hope the reviewer will find persuasive.

**1. Full names of all abbreviations under the tables, particularly Table 4.**

 Done. The main coefficient table (Table 4) and all robustness tables (Appendices B–D) now include a "Description" column spelling out every variable in full, and table notes define the dependent variables and estimation details.

**2. Multilevel models to account for the nested structure.**

 Implemented in three layers. All primary models now use standard errors clustered at the country level (49 clusters). Appendix B reports mixed-effects (random-intercept) probit and ordered probit models with intraclass correlation coefficients and likelihood-ratio tests rejecting the pooled specification (all p < 0.001). Appendix C goes further with correlated random effects (Mundlak–Chamberlain) models that allow the country effect to correlate with the covariates. The Results section (Section 5) is now organised around how each predictor behaves across all of these specifications.

**3. OLS regression as a robustness check, treating trust as continuous.**

 Implemented exactly as suggested. Appendix D reports a linear probability model for TMP and OLS regressions treating the four-point TFT and TN scales as continuous, all with country-clustered standard errors. The concordance is very strong: every sign matches the probit/ordered probit estimates, and the LPM coefficients for TMP are nearly identical to the probit average marginal effects (e.g., 0.139 vs. 0.138 for log GDP per capita). The manuscript concludes that the findings do not depend on the link function or on the ordinality assumption.

**4. More cautious causal language for pooled cross-sectional data.**

 Done throughout. The abstract, introduction, results, and conclusions now use associational language for all empirical claims, and the limitations paragraph states explicitly that the cross-sectional design prevents causal identification and that several regressors are plausibly endogenous to trust.

**5. Treat binary generalised trust (TMP) as a robustness check rather than a core outcome, given conceptual overlap with TFT.**

 We considered this carefully, and we ask the reviewer's indulgence for retaining TMP as a core outcome — with the overlap concern now addressed explicitly in the manuscript (Section 3, conceptual distinctions). Our reasons are three. First, conceptually, TMP elicits a standing moral disposition toward an abstract humanity, whereas TFT elicits the situational extension of trust to a concrete stranger at first contact; following Uslaner (2002) and Newton (1997), these are distinct relational outcomes, and an individual may hold one without the other. Second, empirically, the data confirm they are not interchangeable: several determinants behave differently across the two outcomes — religious importance is strongly negative and significant for TMP but indeterminate for TFT; the institutional and democratic-perception patterns also diverge — which would not occur if the two items measured a single construct. Indeed, this divergence is part of the paper's central finding that trust determinants are domain-specific. Third, the binary item is the canonical measure on which virtually the entire generalised-trust literature is built; retaining it as a core outcome preserves comparability with that literature, while TFT supplies the ordinal, situational counterpart it lacks. Demoting TMP to a robustness check would, we believe, discard precisely the comparison that constitutes the paper's contribution. We hope this reasoning, now stated in the manuscript, satisfies the reviewer's concern.

**6. More literature in the discussion; more systematic comparison with previous empirical studies.**

 The discussion has been substantially expanded. A new "Theoretical Contributions" subsection (Section 7.1) engages systematically with the prior literature: it positions the domain-differentiated findings against unidimensional treatments of trust (Uslaner 2002; Hardin 2002; Nannestad 2008), develops the institutional substitution result against Rothstein and Uslaner (2005) and Rothstein and Stolle (2003), extends Uslaner's shared-fate inequality mechanism to proximate trust domains, and relates the religiosity findings to the bonding/bridging distinction (Putnam 2001; Welch et al. 2007). The Results section now also compares each robust finding with the corresponding prior empirical evidence, and the bibliography has been expanded accordingly.

**7. Add a "Theoretical Contributions" subsection.**

 Done. Section 7.1 ("Theoretical Contributions") presents four contributions: the domain-differentiated account of trust formation; a refinement of the institutional substitution argument (the displacement of proximate reliance occurs without the predicted expansion of generalised trust, a sequencing problem the literature has not articulated); the identification of distributive fairness as the only domain-invariant structural mechanism; and a clarification — made possible by the correlated random effects design — of the level at which cultural mechanisms operate, with religiosity identified as an individual-level mechanism and tolerance as a property of the societal climate. We thank the reviewer again for suggestions that have made the paper considerably stronger.

# Reviewer report: Reviewer response for version 1

**DOI:** 10.21956/openreseurope.24692.r73785

**Published:** 2026-05-15

**Authors:** Andreas Kyriacou

**Affiliations:** 1 Università de Girona, Girona, Spain

This is a competent and ambitious cross-national study on the determinants of generalized trust using World Values Survey data from 2018–2022. The paper combines macro-level variables such as GDP per capita, inequality, institutional quality, urbanization, educational development, religious freedom, migration, and life expectancy with individual-level characteristics including income, religiosity, tolerance, age, education, and democratic perceptions. One of the most interesting aspects of the paper is its distinction between different trust domains: trust in most people (TMP), trust in people met for the first time (TFT), and trust in neighbours (TN). The manuscript is generally well written, clearly structured, and motivated by an important question in the literature on social capital and trust. The use of ordered probit models and average marginal effects is also appreciated. At the same time, the paper has several important weaknesses that prevent it from making strong empirical or policy claims in its current form. The main concerns relate to identification, modelling strategy, interpretation of coefficients, theoretical positioning, and the articulation of the paper’s contribution relative to the existing literature. I would therefore recommend major revisions.

 The paper’s contribution should be more carefully defined and positioned relative to the existing literature. The authors present the study as contributing through: focusing on determinants rather than consequences of trust, integrating macro and micro variables, distinguishing multiple trust domains, and using ordered probit marginal effects. These are reasonable contributions, but the novelty is somewhat overstated. The literature on trust determinants is already very extensive, including work on inequality, institutions, education, religiosity, urbanization, income, and democratic quality. The paper does not sufficiently explain what is genuinely new about its analysis. In my view, the strongest contribution is not the identification of new determinants, but rather the comparison across different forms of trust and the demonstration that some variables operate differently depending on whether trust is abstract, situational, or localized. The distinction between “most people can be trusted,” “trust in people met for the first time,” and “trust neighbours” is probably the manuscript’s most original and useful feature. The authors should therefore reposition the paper around this contribution rather than framing it as “one of the most comprehensive cross-national examinations of generalized trust to date,” which is too strong for a largely observational and cross-sectional study.

 The theoretical framework is broad and informed by a wide literature, but it currently lacks focus and coherence. The paper attempts simultaneously to engage with social capital theory, institutional trust, inequality, democratic legitimacy, urban sociology, education, migration, religion, and welfare-state theory. As a result, the discussion sometimes acquires a “kitchen sink” character in which variables appear to have been included because they are available rather than because they derive from a clearly specified theoretical framework. In particular, Section 3.2.2 does not sufficiently justify the selection of independent variables. The authors should do more to explain why these specific variables were chosen, how they relate to the theoretical mechanisms under consideration, and why certain closely related variables are omitted. A clearer conceptual structure would help considerably. For example, the determinants could be grouped around broader mechanisms such as economic security (GDP per capita, inequality, personal income), institutional reliability (democracy, institutional quality, stability, religious freedom), and social openness or exposure (tolerance, migration, urbanization, religiosity, education). At present, many interpretations appear somewhat post hoc and are introduced only after the coefficients are observed.

 A major concern relates to the empirical design and the treatment of the multilevel structure of the data. Individuals are nested within countries, yet the paper estimates pooled probit and ordered probit models without properly modelling this hierarchy. Many key explanatory variables — GDP per capita, inequality, institutional democracy, educational development, urbanization, and life expectancy — vary only at the country level. Since the sample contains nearly 100,000 individuals, standard errors may be artificially small because respondents within the same country are treated as independent observations. The paper should at minimum cluster standard errors at the country level and, preferably, estimate multilevel or hierarchical models with random intercepts. This issue is especially important given the relatively limited number of countries. Closely related to this is the absence of country fixed effects or alternative strategies to address country-specific omitted variables. Macro-level coefficients may be capturing unobserved historical, institutional, or cultural characteristics such as colonial legacies, welfare-state traditions, Nordic exceptionalism, communist history, or legal origin. I understand that the authors wish to estimate the effect of country-level variables directly, but in that case the paper must be much more cautious in interpreting these coefficients as “determinants” rather than broad cross-country associations.

 The paper also needs to moderate its causal language more consistently. Although the introduction briefly acknowledges that trust itself may affect many of the explanatory variables included in the models, the empirical discussion repeatedly uses language such as “fosters trust,” “erodes trust,” “promotes openness,” or “supports generalized trust.” Given the observational and cross-sectional nature of the analysis, these formulations are too strong. Many explanatory variables are plausibly endogenous to trust, including institutions, inequality, education, democratic legitimacy, and tolerance. The relationship may well run in both directions. For example, generalized trust may itself shape support for redistribution, welfare institutions, and inclusive political systems. The paper would benefit from more disciplined language emphasizing associations or correlations rather than causal effects unless a stronger identification strategy is introduced.

 Several interpretations of the coefficients are also problematic. One of the most striking findings is the negative coefficient on mean years of schooling alongside the positive coefficient on the Education HDI. The authors interpret this as evidence that formal schooling may increase skepticism or critical distance while national educational development promotes generalized trust. This interpretation is not entirely convincing and appears speculative. Alternative explanations such as multicollinearity, and omitted variables. Indeed, the paper later partially retreats from its own interpretation by cautioning readers about the size of the coefficients. Similar concerns apply to the interpretation of the institutional stability variable, where the claim that stability reflects rigidity or institutional immobility appears largely post hoc. The same issue emerges in the discussion of religious freedom, where the argument that pluralism increases caution toward strangers feels insufficiently grounded in the evidence presented.

 The marginal effects section is technically careful and demonstrates a good understanding of the interpretation of nonlinear models. However, the discussion becomes excessively long and repetitive. The paper first interprets coefficients, then average marginal effects, and then level-specific marginal effects across categories. This creates substantial duplication and makes the manuscript difficult to follow in places. In particular, many of the TFT and TN marginal effects are described as being very close to zero across trust levels. The paper should address more explicitly why this occurs and what substantive interpretation should be given to these near-zero AMEs. It is currently unclear whether these patterns reflect genuinely weak substantive effects, measurement limitations, averaging across categories, or instability in the ordered probit specification. More generally, much of the detailed AME discussion should be moved to an appendix, allowing the main text to focus on the most important substantive patterns.

 Despite these criticisms, the paper has several notable strengths. The dataset is large and internationally diverse, the distinction between different trust domains is genuinely useful, and the topic is highly relevant for contemporary debates on social cohesion, polarization, institutions, inequality, migration, and democratic legitimacy. The use of marginal effects is also good practice and improves the interpretability of the ordered probit models. Overall, this is a serious and potentially publishable paper, but currently it remains more exploratory than explanatory. Its main value lies in comparing trust domains and showing that different forms of trust display heterogeneous correlates. However, the paper presently overstates causality, theoretical novelty, and the interpretability of the coefficients. I would therefore recommend major revisions. The manuscript could become suitable for publication if the authors moderate their empirical claims, address the multilevel structure more appropriately, clarify and simplify the theoretical framework, better justify the choice of independent variables, and provide a more disciplined interpretation of the results.

If applicable, is the statistical analysis and its interpretation appropriate?

Partly

Is the study design appropriate and is the work technically sound?

Yes

Is the work clearly and accurately presented and does it engage with the current literature?

Yes

Are the conclusions drawn adequately supported by the results?

Partly

Are sufficient details of methods and analysis provided to allow replication by others?

Yes

Are all the source data and materials underlying the results available?

Yes

Reviewer Expertise:

Political Economy.

I confirm that I have read this submission and believe that I have an appropriate level of expertise to confirm that it is of an acceptable scientific standard, however I have significant reservations, as outlined above.

# Response

**Published:** 2026-08-27

**Authors:** Josep Navarro-Ortiz

**Affiliations:** Anàlisi Econòmic, Universitat de Valencia, Valencia, Valencian Community, Spain

We are very grateful for this thorough and constructive report. The review shaped the revision more than any other input: version 2 repositions the paper's contribution, replaces the descriptive variable discussion with an explicit theoretical framework, and rebuilds the entire inference strategy around the multilevel structure of the data. We respond point by point.

**1. Contribution overstated; reposition around the domain comparison.**

 We agree, and we have repositioned the paper exactly as suggested. The claim of being "one of the most comprehensive cross-national examinations of generalized trust to date" has been removed. The rewritten Introduction now states in its second paragraph that the joint, domain-comparative analysis of TMP, TFT, and TN is "the paper's key empirical innovation," and the reordered contribution statement places the multi-domain comparison first. A new "Theoretical Contributions" subsection (Section 7.1) develops this positioning: its first contribution argues that theories treating trust as a unidimensional disposition systematically mischaracterise the effects of institutions and culture, which redistribute trust across relational targets rather than raising or lowering it uniformly. The abstract has been rewritten accordingly, and the paper now describes itself throughout as distinguishing robust associations from model-sensitive ones rather than as a comprehensive account of determinants.

**2. Theoretical framework lacks focus; variable selection insufficiently justified; "kitchen sink" character.**

 Version 2 contains a new Section 3 ("Theoretical Framework") that implements the reviewer's suggestion directly. The determinants are now organised around three mechanism domains — (i) economic security and distributional fairness, (ii) formal institutions and the architecture of trust, and (iii) community structure, cultural openness, and education — from which six ex-ante hypotheses are derived, each with explicit directional predictions per trust domain (summarised in Table 1 of Section 3.4). All interpretations in the Results section now test these pre-stated hypotheses rather than being introduced after the coefficients are observed. Consistent with this discipline, five country-level variables from version 1 that fall outside the framework (BTI institutional stability, the Education HDI, religious freedom, migration rate, life expectancy) have been removed from the preferred specification; Section 4.2 states both the theoretical reason and the empirical corroboration (none survives the clustering correction). Two theoretically motivated individual-level variables (gender, rural residence) have been added, the latter operationalising the community embeddedness mechanism.

**3. Multilevel structure: cluster standard errors at minimum, preferably multilevel models.**

 Fully implemented, and extended. All primary models (Table 4) are now estimated with standard errors clustered at the country level (49 clusters). In addition: (a) Appendix B reports mixed-effects probit and ordered probit models with country random intercepts, including intraclass correlation coefficients (0.125 for TMP, 0.094 for TFT, 0.075 for TN), likelihood-ratio tests against the pooled models (all p < 0.001), and verification of numerical stability across 7, 15, and 30 quadrature points; (b) Appendix C adds correlated random effects (Mundlak–Chamberlain) models that relax the random-effects orthogonality assumption; (c) Appendix D adds linear specifications and wild cluster bootstrap p-values for the key structural coefficients, addressing the reviewer's implicit concern about the cluster count. As the reviewer anticipated, the correction is consequential: several variables that appeared highly significant under unclustered standard errors do not survive, and the paper now reports this transparently as one of its central findings.

**4. Country fixed effects / caution in interpreting macro-level coefficients as "determinants."**

 We address this on both fronts. Country dummies in a probit framework would create the incidental parameters problem and absorb all country-level regressors; the CRE specification of Appendix C is the standard solution, allowing the country effect to correlate with the covariates. The joint Wald tests on the Mundlak means reject the random-effects assumption for TMP (p < 0.001) and TFT (p = 0.046), confirming that the concern was warranted; all individual-level conclusions survive the correction, while country-level coefficients are explicitly framed as cross-country associations identified under the caveats the reviewer describes (Appendix C discusses this directly). The Results section is now organised into an explicit robustness hierarchy — cross-domain anchors, domain-specific mechanisms, and model-dependent associations (Table 5) — so that no macro-level coefficient is interpreted beyond what its behaviour across specifications supports.

**5. Causal language.**

 We have conducted a systematic revision. The abstract, introduction, results, and conclusions now use associational language ("is associated with," "accompanies," "correlates with") for all empirical claims; causal vocabulary is confined to the statement of theoretical mechanisms, clearly marked as hypotheses. The limitations paragraph states explicitly that the cross-sectional design prevents causal identification, that several regressors are plausibly endogenous to trust, and that causality may run in both directions — including the reviewer's example of trust shaping support for redistribution and inclusive institutions.

**6. Problematic interpretations (MYS vs. EduHDI; institutional stability as "rigidity"; religious freedom and pluralism).**

 We agree these interpretations were speculative, and they are gone. EduHDI, religious freedom, and institutional stability have been removed from the specification (point 2 above), and their post-hoc interpretations with them. Mean years of schooling is retained for theoretical completeness but is now reported as non-robust ("model-dependent" tier), with no substantive interpretation attached: "the country-level educational stock exerts no reliable independent association with trust in these data" (Section 5). The individual-level education gradient — which is robust in every specification — carries the educational interpretation instead.

**7. Marginal effects section too long and repetitive; near-zero AMEs unexplained; move detail to an appendix.**

 Implemented. The detailed level-specific AME tables have been moved to Appendix A; the main text (Section 6) now discusses only the substantively important patterns. Near-zero AMEs are no longer narrated case by case: the manuscript explains that they correspond to coefficients that are non-significant after the clustering correction, and these variables are classified in the "model-dependent" tier of Section 5 precisely so that the reader knows they should not be interpreted. The duplication between coefficient discussion, aggregate AMEs, and level-specific AMEs has been eliminated by assigning each a distinct role (direction and robustness in Section 5; probability-level magnitude in Section 6; full detail in Appendix A). We hope the reviewer finds that version 2 delivers the more disciplined, exploratory-to-explanatory repositioning the report called for, and we thank him again for a review that materially improved the paper.
